
JOURNAL INFORMATION
==============================
NLM Title Abbreviation: Innov Aging
Journal ID: Innov Aging
NLM Journal ID: 101703706
Journal ID: innovateage

Innovation in Aging

EISSN: 2399-5300
Publisher: Oxford University Press

ARTICLE INFORMATION
==============================
PMCID: PMC10938894
PMID: 38487513
DOI: 10.1093/geroni/igad104.3806
Article ID: igad104.3806
Article version: 1
Subjects: Participant Index, AcademicSubjects/SOC02600

Participant Index

Publication date: 2023 Dec
Electronic publication date: 2023 Dec 21
Volume: 7
Issue: Suppl 1
Issue title: Program Abstracts from The GSA 2023 Annual Scientific Meeting, “Building Bridges > Catalyzing Research > Empowering All Ages”
First page: 1189
Last page: 1233
Copyright: © The Author(s) 2023. Published by Oxford University Press on behalf of The Gerontological Society of America.
Copyright year: 2023
License: This is an Open Access article distributed under the terms of the Creative Commons Attribution License (https://creativecommons.org/licenses/by/4.0/), which permits unrestricted reuse, distribution, and reproduction in any medium, provided the original work is properly cited.
License URL: https://creativecommons.org/licenses/by/4.0/

Page count: 45

==============================

### Participant Index

Arabic numbers refer to session numbers.
Duplicate numbers indicate the participant is listed as an author on multiple abstracts within a session.

A
Aamponsah, Abigail: 7540
Aarts, Sil: 1260, 7800
Aartsen, Marja J.: 1220
A'Azman, Shamirah: 4025
Abadir, Peter M. M.: 1060, 9000, 9020
Abaei, Elnaz: 2170
Abbas, Jihan: 9030
Abbott, Katherine M.: 4435
Abboud, Anissa: 3360, 4010
Abdallah, Lisa: 1080, 5200
Abdel Magid, Hoda S.: 1015
Abe, Takaaki: 7370, 9010
Abebe, Ephrem: 7080
Abedi, Ali: 7680
Abedine, Reuben: 9320
Abel, Erica A.: 9030
Abelson, James: 9020
Abelson, Jamie: 9020
Abi Chahine, Maya: 9500
Abner, Erin L.: 9020
Abraham, Alison G.: 2240, 2365
Abrahamse, Paul: 1085
Abrams, Leah: 3225, 3440, 9030
Abramson, Tobi A.: 7760
Abrantes, Ana M.: 1030
Abreu, Taiane: 7060
Abshire Saylor, Martha: 1035, 3415, 4170
Abudurezake, Abulaiti: 9000
Abujarad, Fuad: 2085
Acevedo-Fontanez, Adrianna I.: 9020
Acharya, Sandeep: 9010, 9020
Acierno, Ron: 7010
Adachi, Jonathan: 4268, 7220, 7680, 9020
Adamek, Margaret E.: 2010, 7190, 7200
Adams, Anne: 9320
Adams, Hannah: 7510
Adams, Swann Arp: 4005
Adams-Price, Carolyn E.: 1135, 2040, 3425
Adar, Sara: 7320
Addison, Odessa: 7340, 7350, 7840
Addo-Mensah, Dorothy: 4380
Adekoya, Adebusola: 1165
Adekunbi, Daniel: 9000
Adeleye, Khadijat: 9010
Adelman, Ronald: 7360
Adelnia, Fatemeh: 9010
Adeniji, Dolapo O.: 7190, 7200
Adepoju, Omolola E.: 2085
Adesanya, Ikmat: 4040
Adeyemi, Oluwaseun: 7130, 7840
Adjei, Naomi: 4045
Adkins-Jackson, Paris B.: 1345, 2225
Adler, Charles: 9030
Adonis-Rizzo, Marie T.: 4132, 7480
Aflagah, Erica: 7510
Ågård, Pernilla: 4330
Agarwal, Arunika: 7940
Agboji, Aderonke: 7160
Agha, Agha A.: 4130, 5110
Aghaali, Elham: 7660
Agmon, Maayan: 3125, 4265
Agran, Dylan: 4406, 7000
Agrawal, Yuri: 2150, 3390
Agrigoroaei, Stefan: 7960
Agrimson, Kellie: 8900
Aguila Vega, Emma: 9030
Aguilar, Iris: 7700
Aguirre, Alyssa: 5235
Aguirre, Annabella: 3155
Agyapong, Nakesha: 9030
Agyarko Poku, Thomas: 7790
Agyemang, Lillian: 7220, 9010
Ahmad, Nina: 2035
Ahmad, Suzan: 4105
Ahmad, Zeba: 9030
Ahmed, Adeeb: 3295
Ahmed, Ali: 3490
Ahmed, Neelam H.: 4025
Ahmed, Yusuf K.: 9000
Ahn, Eunhye: 7960
Ahn, Hyochol: 7460
Ahn, Jinhyun: 9030
Ahn, SangNam: 7960
Aho, Velma T E.: 1225
Ahrens, Monica: 4545, 9020
Ai, Amy L.: 2280, 7410
Ai, Meishan: 7100
Aichele, Stephen R.: 4185
Ailey, Sarah: 3445
Ailshire, Jennifer A.: 1125, 1165, 2050, 2175, 3160, 3465, 4395, 5050, 7380, 7540, 7720, 7890, 9020, 9310
Ainsworth, Laura A.: 9020
Aisagbonhi, Omonigho: 8900
Aitken, Rebecca: 1130
Ajja, Rahma: 3505, 4350
Ajrouch, Kristine J.: 1355, 2100, 3440, 4285, 9210
Ajtai, Bela: 7830
Akagi, Yuya: 4505, 9000
Akbary Moghaddam, Vaha: 9020
Akgün, Kathleen A.: 9030
Akiguchi, Ichiro: 9010
Akinbami, Yewande: 9000
Akintola, Kehinde G.: 9010, 9310
Akinwuntan, Abiodun: 9330
Akksilp, Katika: 3440
Akter, Nahida: 3020, 7160
Akushevich, Igor: 2015, 2180, 3470, 3510, 5015, 5110, 7100, 7370, 7620
Al Snih, Soham: 9030
Alahmari, Saeed: 7100
Alaie, Iman: 9030
Alain, Gabriel N.: 2260
Alam, Rifat B.: 7300
Alavi, Mohammad Hesam: 4265
Al-Azawi, Abdulwahab: 7160
Albala, Cecilia: 3465
Albers, Elizabeth A.: 2130, 3305, 7380, 7420
Albers, Hannah: 1285
Albert, Steven M.: 2190, 3530, 9020, 9030
Alberth, Andrew: 3385, 7610
Albertini, Marco: 9030
Alberts, Susan: 1195
Albright, Amy: 7910
Albright, Karen: 7300
Alcantar, Sandy: 1135
Alchin, Tucker: 7800
AlDossary, Heba: 7590
Aldrich, Vicky: 2300
Aldridge, Melissa D.: 2235, 3335
Aldwin, Carolyn M.: 1160, 1390, 7510, 7580
Aleksic, Sandra: 1125
Alexander, Gregory L.: 3175, 9020
Alexander, Ivana: 3360
Alexander, Karah: 7620
Alexander, Karen J.: 2315
Alexander, Laura: 7110
Alexander-Bady, Janae: 7840
Alfares, Hadil: 9010
Alfaro, Ana J.: 3250
Alfaro, Ana J.: 9010
AlGhatrif, Majd: 9020
Ali, Imran: 4165
Ali, Mohab: 7390
Ali, Sanna: 9030
Ali, Sumbleen: 1255
Ali, Talha: 3360, 4010
Ali Morshed, Hanan: 3170
Alkabbani, Wajd: 4280
Allala, Sai Chaithra: 4415
Allan, Alexa C.: 7560, 9020
Allard, Eric S.: 7040, 9000, 9020
Allemand, Mathias: 7060
Allen, Julie Ober: 1285, 9020
Allen, Katy: 9010
Allen, Kelli D.: 4165
Allen, Laura D.: 2160, 9000
Allen, Odell: 3560
Allen, Rebecca S.: 1365, 7390, 7910, 9210
Allen, Tammy: 9010
Allison, David: 9020
Allore, Heather: 1305, 2360, 3230, 7300, 7360, 7400, 7450, 7540, 7890, 7990
Allsopp, Richard: 2380, 5060, 7110
Almardawi, Ranyah: 7130, 9000
Almeida, David M.: 1360, 3135, 3230, 3545, 4425, 4475, 5045, 5080, 7050, 7390, 7610, 9020
Almeida, Paulo: 7320
Almidani, Louay: 1345, 2135, 9030

© The Author(s) 2023. Published by Oxford University Press on behalf of The Gerontological Society of America.
This is an Open Access article distributed under the terms of the Creative Commons Attribution License (https://creativecommons.org/licenses/by/4.0/), which permits unrestricted reuse, distribution, and reproduction in any medium, provided the original work is properly cited.

Almiron, Maby: 7380
Alston, Daniel M.: 3350
Altenburger, Peter: 7060
Altintas Akin, Ozlem: 9020
Altona, Janissa: 2370
Alvarez, Bianca: 7500
Alvarez, Elizabeth: 5085
Alvarez, Loreli: 3545, 4145, 7090
Alvarez Azanedo, Gabriela: 7110
Alvear Portaccio, Marcela: 3465
Alwan, Elizabeth: 9020
Alwerdt, Jessie: 5165, 9030
Alyssa, Camp: 3425
Alzahrani, Mallak: 5230
Amanambu, Peace: 9000
Amano, Takashi: 3310, 4210, 4266, 7690, 7990, 9000, 9030
Amaza, Hannatu: 9000
Ambike, Satyajit: 7060
Ambroise, Alexis Z.: 1070
Ambrose, Madison: 3300
Ameer, Fathima: 7370
Amgalan, Anar: 9020, 9030
Amin, Iftekhar: 3205
Amini, Reza: 7280
Aminu, Abodunrin: 4455
Amir, Omar: 7860
Amjad, Halima: 2185
An, Jaesung: 7610, 7920
An, Jeongwi: 7030, 7570
An, Ping: 9020
An, Qiuling: 9030
An, Yang: 4035
Anable Eme, Vivian: 4460
Ananthasubramaniam, Aparna: 5010
Ances, Beau: 9030
Anctil, Reid: 9030
Andel, Ross: 3050
Andel, Ross: 7020, 7420, 7990, 9000
Andersen, Julie K.: 2335
Andersen, Stacy L.: 1040, 5180, 7830
Andersen, Troy C.: 1150
Anderson, Aiden: 7100
Anderson, Andrea M.: 7630
Anderson, Brett E.: 7020
Anderson, Erik: 7680
Anderson, Keith A.: 1095, 2130, 9000, 9010
Anderson, Loretta: 1085, 5205
Anderson, Rozalyn: 5060
Anderson, Ruth: 3540, 4380
Anderson, Taharka: 9000
Anderson-Hanley, Cay: 3480
Andersson, Bjoern: 3490
Andrade, Allen: 4266, 7190
Andrade, Flavia: 3050
Andrasfay, Theresa: 3065, 7890, 9030
Andreoletti, Carrie: 1080
Andrew, Melissa: 7650
Andrew, Simon: 2315
Andrews, Ebony: 9010
Andrews, Heather: 9030
Ang, Alvin: 7100
Ang, Gregory: 4385
Angel, Jacqueline L.: 9030
Anghel, Ionut: 1220
Anilkumar Hemalatha, Sruthi: 7200, 7940
Anim-Koranteng, Comfort: 7330
Anisa Simoni, Anisa: 4300
Ankuda, Claire K.: 3575
Anosike, Chioma A.: 2125
Anquillare, Elizabeth: 7410
Ansaldo, Ana Inés: 7680
Anstey, Kaarin J.: 3570, 9000, 9110
Antequera, Felipe: 9030
Antoan, Jordann: 7360
Antonakakis,, Antigone: 9000
Antonietti, Michael: 7600
Antonio-Villa, Neftali E.: 3160, 4395
Antonoplis, Stephen: 4300
Antonsdottir, Inga M.: 4430
Antonucci, Toni C.: 1255, 2100, 4285, 4325
Apgar, Dawn: 5170
Apiribu, Felix: 7790
Appiah, Abigail Acheampomaa: 7790
Appiah-Kusi, Evans: 7700
Applebaum, Allison J.: 4030
Applebaum, Jennifer W.: 9020
Applebaum, Robert A.: 1115, 2200, 3190, 4435
Appleby, James: 3055
Arabi, Hana: 9500
Arain, Salman: 7840
Aranda, Maria P.: 7700, 7720, 9020
Aratia, Shahrose: 9330
Araujo, Lia: 3130, 7060, 7200
Arbeev, Konstantin: 2015, 7110, 7370, 7800
Arcila-Mesa, Mauricio: 7130, 7840
Ardekani, Azam: 7140
Ardelt, Monika: 1390
Ardisson Korat, Andres Victor: 1290
Arellanes, Isabella C.: 4395
Arensberg, Mary Beth: 9020
Arevalo-Flechas, Lyda: 7180, 9010
Arishenkoff, Shane: 7820
Armah, Henrietta: 7020
Armendariz, Marina: 5130
Armistead, Lori: 7150
Armstrong, Alissa: 1325
Armstrong, Cheryl: 7480
Armstrong, Grace: 4525
Armstrong, Joshua J.: 1280
Arnette, Shannon: 3300
Arnold, Michelle: 1295, 4110, 7960
Arora, Kanika: 7760
Arora, Neeraj: 9000
Arpino, Bruno: 4530
Arriola, Nora B.: 7150
Arthanat, Sajay: 9010, 9550
Artyomov, Maxim: 4065
Aruah, Simeon Chinedu: 4130
Aruscavage, Nancy: 1150, 2035, 9020
Asakura, Kyoko: 9010
Asante, Samuel: 3035, 7480
Ash, Samson: 5235, 7030
Ashar, Perisa: 9020
Ashcraft, Alyce S.: 7160
Ashe, Maureen: 3275, 7550, 9000
Ashebir, Sophia: 7910, 9000, 9010
Asher, Irit: 7720
Asher, Merav: 3125
Ashida, Sato: 2120, 3445, 3540, 7440, 7470
Ashiqueali, Sarah A.: 4475, 7110
Ashirifi, Gifty D.: 7190, 7200
Ashrafi, Sadia Anjum: 7610
Asiimwe, Stephen: 9000
Ask, Levi: 7170
Asquith-Heinz, Zayla: 2120, 3015
Assi, Sahar: 2150
Aswani, Truphosa: 3240
Atchison, Karley: 7580
Atherton, Olivia E.: 4300, 9570
Atkinson, Elizabeth: 1325
Attuquayefio, Tuki: 3570
Atul Patil, Vaishnavi: 9000
Au, Rhoda: 1040, 7100, 9010
Audley, John: 9000
August, Kristin J.: 5055
augustine, Matthew: 9000
Austad, Steven: 1060, 3575, 9020
Austin, Thomas: 7110
Au-Yeung, Wan-Tai M.: 4495
Avallone, Margaret A.: 1130, 7290
Avent, Elizabeth S.: 2220
Avigezer, Avital: 2345
Avila, Jaqueline C.: 3160, 7320
Avis, Nancy E.: 5155
Awad, Kamal: 7370
Awata, Shuichi: 7280
Aworinde, Tofunmi: 4470
Axelrad, Hila: 7290
Ayalon, Liat: 1245, 2105, 3425
Ayele, Roman A.: 7300
Ayieko, Sylvia: 7610
Ayisi-Boahene, Givenchy: 5030, 7120
Ayoob, Carol: 4215
Ayrat, Ansar: 9030
Azar, Martina: 7770
Azhar, Gohar: 1085, 4130, 7100, 7370, 7880, 9000, 9020
Azizi, Linda: 8900
Azizi, Mehrnoosh: 1200
Azuero, Andres: 4340, 7680

B
Baarbé, Julianne: 4280
Babar, Huma: 4030
Babazadeh, Romina: 7420
Babbs, Gray: 4000
Babulal, Ganesh: 9030, 9330
Baby, Eliza: 1260
Bachman, Noa: 3275
Bachner, Yaacov G.: 7920, 9030
Bachrach, Rachel L.: 7580
Backhaus, Ramona: 9010
Backman, Annica: 3180
Bacon, Suzette Mizutani: 1310
Bacsu, Juanita-Dawne: 1200, 9000
Badawi, Sarah: 7770
Baek, Jihye: 7550, 9030
Baek, Jiyeon: 9000
Baek, Sang Yi: 7850
Baek, Sehyun: 9010, 9030
Bagaajav, Ariunsanaa: 7380
Bagley, Olivia: 7800
Bahnson, Judy L.: 4355
Bai, Xue: 1130, 2230, 4020, 4520, 7040, 7090, 7250
Baier, Rosa: 4190, 4290, 7540, 7640
Baig, Farah: 3460, 7250
Baik, Dawon: 7400, 7470
Baik, Sol: 5025, 7530

Bailey, Amelia: 9030
Bailey, Dionne: 1065, 3300
Bailey, Donald E.: 3540
Bailey-Arafah, Micah: 7630
Baillargeon, Emma M.: 2155
Baim-Lance, Abigail: 1375, 7820, 9000
Bajdek, Nicole: 3480, 9000
Bakas, Tamilyn: 7850
Bakeman, Roger: 5015
Baker, Jillian: 9010
Baker, Nathan: 9000
Baker, Tamara A.: 2115
Baker, Zachary G.: 1025, 3460, 9000
Bakerjian, Deb: 3005
Bakitas, Marie: 9020
Bakk, Louanne: 7220, 7700, 9010
Baksh, Magda: 7620
Bakulski, Kelly M.: 7370
Bakx, Pieter: 7120
Balachandran, Sowmya: 2315
Balantrapu, Swathi: 9010
Balasubramanian, Aparna: 7650
Baldivieso, Valeria C.: 7620
Baldwin, Julie: 7400
Baldyga, Kathryn: 9030
Balestra, Dennys P.: 2280
Balkan, Em: 4000
Ball, Karlene K.: 7060, 7990
Ballard, Erin: 3050
Ballard, Stephanie A.: 7790
Ballinger, Tarah: 7410
Ballout, Suha: 9510
Ballreich, Jeromie: 5160
Balmert Bonner, Lauren: 4310
Balmuth, Alexa: 1175, 3090, 9000
Baloni, Priyanka: 5180, 7880
Bamgbade, Benita A.: 4134, 7830
Bamonti, Patricia M.: 4095, 7340
Ban, Si Woo: 9540
Banarjee, Chitra: 5075, 7150, 7820, 9030
Banarjee, Reema: 5060, 7880
Banaszak-Holl, Jane: 2350
Bandeen-Roche, Karen: 1110, 1370, 3240, 4485, 9010
Bandosz, Piotr: 2255
Band-Winterstein, Tova: 4520
Banerjee, Sube: 3335
Bangen, Katherine: 8900
Bangerter, Lauren: 3030, 5000, 9000, 9010
Bankole, Ayomide Okanlawon: 4380
Bankole, Ayomide Okanlawon: 9000
Banks, Jack: 7840
Banks-Wright, Hortense: 9000
Banner, Davina: 9030
Banner-Lukaris, Davina: 5105
Bannon, Sarah: 2035, 4430
Bansal, Deven M.: 5065
Banskota, Nirad: 2195
Bao, Dapeng: 1260
Barajas, Jordan: 7100
Baranyi, Gergő: 1015
Barbara, Rita: 7340, 9000
Barbee, Harry: 4000
Barbour, Elizabeth: 7970
Barclay, Ruth: 7940
Bardales, Rhonda: 7280, 9020
Bardo, Anthony R.: 5080
Bareham, Bethany K.: 4540, 9010
Barela, Lance: 7900
Barger, Kathryn: 1290
Barkley, Morgan: 9020
Barlow, Meaghan A.: 7040
Barman, Papai: 7940
Barnard, Ryan T.: 7630
Barnason, Susan A.: 9010
Barnes, Deborah E.: 1010, 2035, 3490, 4035
Barnes, Haley: 3500
Barnes, Lisa L.: 2120, 2225, 4100
Barnes, Stacy: 7310
Barnes, Virgile P.: 3365, 7260
Barnett, Brianna: 7050, 7910, 7960
Barnett, Michael D.: 7080, 7590, 7900
Barr, Paul: 9010
Barrón, Yolanda: 9010
Barragan, Noel: 7700
Barreto, Esteban A.: 4430
Barrett, Anne E.: 4230, 7060, 7070, 7570, 7590, 7600
Barrett, Blake: 7150
Barrett, Tyler: 1010
Barrie, Helen: 3260
Barry, Kathryn H.: 7340
Barry, Lisa C.: 3525, 4115, 7030
Barry-Dutro, Erin: 5025
Bartel, Maya: 7830
Bartke, Andrzej: 4475, 9020
Barve, Shirish: 9000
Barzilai, Nir: 1125
Basaran Sahin, Duygu: 3080, 4175
Bashian, Hannah M.: 9010
Basic, Ajla: 3035, 5125, 9000
Basile-Alvarez, Martin Roberto: 4395
Basinger, Erin D.: 1235
Basinska, Kornelia: 7160
Basisty, Nathan: 1325, 5060, 7880
Basrai, Zavera K.: 9530
Bass, David M.: 9020
Bastow, Skyler: 7590
Basu, Anirban: 1115
Basu, Koushambhi: 1335
Basu, Rashmita: 7760
Batchelor, Melissa: 7300
Bate, Angela: 2375
Bateman, James: 1320
Batista-Malat, Eleanor: 4375, 7720
Batsis, John A.: 4268, 7360, 7680, 9000
Battaglia, Catherine: 7300
Battar, Saraswathy S.: 3110
Battle, Brian: 9000
Bauguess, Caleb: 4350
Bauldry, Shawn: 4535, 7920
Bauman, Alexandra: 7590
Baumann, Sara: 9020
Baumbusch, Jennifer: 1335
Baumgart, Matthew: 1265, 3020, 7420
Baumgartner, Lisa M.: 7190
Baur, Joseph: 2205, 9020
Bautista, Kirsten: 9030
Bautmans, Ivan: 1125
Baxter, Pamela: 1145
Bayer, Thomas: 7620
Bayles, Gregory N.: 7700
Bayne, Alycia: 1065, 3075
Bazurto, Elizabeth: 9420
Beach, Brian: 3265, 3370, 4015
Beach, Scott: 7530
Beach, Thomas G.: 9030
Beachy, Tim: 3540
Beadle, Janelle N.: 4045
Beam, Christopher R.: 7370, 7540
Beamer, Brock: 7330
Bean, Jonathan F.: 9010
Beard, John: 1110
Bearden, Valerie: 9030
Beattie, Zach: 4100, 4495
Beatty, Jennifer F.: 9570
Beaty, Carissa: 3055
Beauchamp, Jennifer: 9010
Beauchamp, Marla: 4268, 5070, 7170, 7680, 7780
Beaudoin, Kayla D.: 5100
Beaudreau, Sherry A.: 4300
Beaulaurier, Richard L.: 9030
Beaulen, Audrey: 9010
Beck, Emorie D.: 1255
Beck, McKenzie: 7960
Beck, Monica L.: 3140
Becker, Clemens: 3550
Becker, David: 3365, 4190
Becker, Todd D.: 2020, 5015, 5025
Becker, Todd D.: 9010, 9100
Bedard, Michel: 3570, 7060
Beeber, Anna S.: 2290, 4025, 4280, 4380
Beech, Bettina M.: 3450
Beer, Jenay M.: 9320
Beetle, Christopher: 7620
Begum, Momotaz: 9010, 9550
Behal, Anil: 9020
Behar-Zusman, Victoria: 4340
Behrens, Liza: 3395, 5030, 5145
Beitelshees, Amber: 4080
Béland, François: 5220
Belanger, Emmanuelle: 7620
Belchior, Patricia: 7620
Beld, Matthew: 9020
Belfi, Amy: 4045
Belin de Chantemele, Eric: 9030
Bell, Gregg: 3005
Bell, Janice: 7280, 7750
Bell, Julia: 9000, 9020
Bell, Justice: 1325
Bell, Kristina: 9010
Bell, Mallory J.: 7600, 9010
Bell, Sue Anne: 2295
Bell, Tyler R.: 7060
Bellettiere, John: 3390
Belli, Hayley: 7230
Bellizzi, Gabriela: 7920
Bello-Chavolla, Omar Yaxmehen: 3160, 4395
Belsky, Daniel W.: 1110, 1320, 2285
Beltran, Jazmin: 7160
Beltran, Susanny: 4510, 7900, 9030
Belza, Basia: 7830, 7910, 9010
Ben Abdesslem, Amel: 7210
Ben Eliezer, Aya: 7720
Ben-David, Boaz M.: 9420
Benden, Mark E.: 7430

Bender, Alexis A.: 1375, 3285, 3565, 9030
Bender, Anna E.: 4525, 7590
Ben-Ezra, Menachem: 3275
Benjamin, Cynthia: 2035
Benjamin, Susan: 7660
Benjamini, Dan: 3495
Benjasirisan, Chitchanok: 7770, 7840, 7960
Bennett, Jeanette: 1320
Bennett, Jenil: 9010
Benoit, Ellen: 5115
Bensimon, Moshe: 1140
Benson, Clark: 7360
Benson, Jacquelyn J.: 4415
Benson, Lizbeth: 5165
Bentley, Megan: 2085
Bento, Maria José: 7320
Benton, Donna: 4420, 5120, 7700
Benzel, Heather: 9020, 9520
Beran, Krista M.: 7700
Berbakov, Maria E.: 9030
Berbaum, Michael: 4465
Berceanu, Denisa Cristina-Alina: 7960
Berg, Karina: 2300
Berg, Kristen A.: 1170
Bergeman, Cindy S.: 1070, 1075, 3245
Bergen, Gwen S.: 9010, 9100
Bergeron, Caroline D.: 1200, 7410
Bergh, Marissa S.: 7120
Bergman, Elizabeth J.: 2245, 3350
Bergman, Nathaniel: 9020
Bergren, Stephanie: 4050, 7080
Bergström Af Ekenstam, Amelie: 7040
Bergstrom, Jaclyn: 7820, 8900
Berg-Weger, Marla L.: 2250, 9000, 9030
Berhane, Kiros T.: 1110
Berish, Diane: 1300, 4315, 7160, 7620, 7830
Berkley, Amy S.: 9030
Berkman, Lisa F.: 3225
Berkowsky, Ronald W.: 5065
Berman, Jacquelin: 7760
Bernard, Hannah: 7440
Berndt, Annette: 9000
Bernhardt, Paul: 9000
Bernhardy, Christina: 7230
Berning, Michelle: 3460
Bern-Klug, Mercedes: 1100
Bernstein, Laura E.: 7450
Berridge, Clara: 1280
Berry, Katherine: 1130
Berry, Sarah: 2385
Berta, Whitney: 9550
Bertisch, Suzanne: 4355
Bertogg, Ariane: 1225
Bertola, Laiss: 3160
Besser, Lilah: 3490
Bessette, Lily G.: 9020
Best, Rachel D.: 9020
Best, Ryan: 4185, 9020, 9030
Best, Thomas M.: 5125
Bethell, Jennifer: 4515, 7650
Betley, Wendy: 7310
Bettencourt, Ricki: 9010
Beydoun, May: 9000
Beyene, Kebede: 9030
Beyer, Fiona: 4455
Bhadra, Nivedita: 5180
Bhagianadh, Divya: 1030
Bhanbhro, Sadiq: 4245
Bhandari, Neeraj: 7210
Bhansali, Rebekah: 2290
Bharucha, Haniya: 4405
Bhat, Aarti C.: 5045
Bhatnagar, Neera: 7170
Bhatta, Tirth R.: 1070, 3485, 5010, 9010
Bhattacharjya, Sutanuka: 1120
Bhattacharyya, Kallol Kumar: 7400, 7990
Bhookya, Divya: 1120
Bhowmick, Pallabi: 4020
Bhyra, Nagaveni: 9010
Bibbo, Jessica: 2060, 5195
Bibriescas, Natashia: 3545, 4145
Bieber, Heather: 7180
Bielak, Allison A.: 7080
Bielsten, Therése: 7300
Bier, Nathalie: 7620
Bierman, Alex: 7770
Biggar, Virginia: 2180
Bilalaga, Mariah Malak: 9030
Bilecen, Basak: 3530
Bilgel, Murat: 2195
Billinger, Sandra: 7330
Billings, Pamela: 7160
Binder, Carrie: 3090
Birabwa, Joy C.: 5110
Birchander, ellen: 2245
Birditt, Kira: 4230, 4235, 4425, 5035, 5045, 7390, 7630, 9000
Birkeland, Robyn W.: 3570, 7420
Birmingham, Cheryl L.: 9030
Bischoff, Brian: 9010
Bishop, Alexander: 2390, 7050, 7460, 7910, 7960
Bishop, Christine E.: 1115
Bishop, Kellee: 7590
Bishop, Nicholas J.: 2360, 9020
Bishop, Teddy: 4290
Bishop-Royse, Jessica: 3445
Biss, Renée K.: 4405
Bisset, Elise S.: 9010
Bix, Gregory: 9010
Bizon, Jennifer L.: 1030
Bjarnadottir, Ragnhildur I.: 7860
Bjork, Jonas: 2340
Bjornson, Samantha: 4025
Black, Kathy: 4150
Black, Sheila: 7060
Blackman, Laurie: 9030
Blackmon, Emma: 9030
Blackshear, Chad: 9200
Blackwell, GinaMari: 9330
Blackwell, Terri: 3475, 3500
Blain, Hubert: 3550
Blair, Beth: 3045
Blake Anuskiewicz, Blake: 9010
Blanco, Ana: 3260
Blanco-Arroyo, Nancy Angelica: 2290
Blaskewicz Boron, Julie: 4268, 7020, 7550, 7680
Blaxton, Jessica: 1075
Blazer, III, Dan: 1370
Bleijlevens, Michel H.: 3340
Blench, Karl: 9110
Block, Laura: 7360, 9020
Blondet, Ricardo: 7840
Bloom, David E.: 7940
Bloom, Rachel F.: 2075, 7300
Bloomberg, Mikaela: 2015
Blount, Amber: 7430, 9010
Blow, Fred: 7390
Blozis, Shelley A.: 2290
Bluck, Susan: 1390, 7380, 7580, 7900, 7970
Blues, Elwood: 4410
Blum, Moritz: 7820
Blumen, Helena: 3160, 3480, 9020
Bluth, Karen: 3320
Bluthenthal, Ricky: 2175
Boafo, Jonelle: 2235
Boakye, Alfred: 7290
Boardman, Charles: 7440
Boateng, Augustine CO.: 3485, 4145, 8900
Boaz, Timothy: 9020
Bobitt, Julie: 3075, 7710, 9010
Bobry, Melanie D.: 1335
Bockting, Walter: 9100
Bodenheimer, Lisa: 7290
Bodkin, Stephan: 9020
Bodner, Ehud: 3275
Boehmer, Leigh: 7500
Boer, Bram de: 2370
Boerner, Kathrin: 2245, 3130, 4435
Bogdan, Paul: 9030
Bogdan, Ryan: 3520, 4320, 9020
Boger, Jennifer: 3255, 4245
Bogoniewski, Aleks: 9000, 9010
Bohnsack, Alanna: 7660, 7830
Boivin, Christopher: 8900
Bolkan, Cory: 1365, 3250
Boltz, Marie P.: 1300, 2305, 2370, 3330, 3340, 4315, 5030, 5145, 7160, 7620, 7830
Bonaguidi, Michael: 3070
Bonaparte, Jimeace M.: 9020
Boncella, Katie: 9020
Bondi, Mark W.: 4340
Bonds Johnson, Kalisha: 3535, 4010
Bonifas, Robin: 9030, 9300
Bonner, Alice: 3175
Bonner-Cozad, Aisha: 5040
Bonomo, Melia: 9110
Boockvar, Kenneth: 7890, 9000
Booker, Emma: 7700
Booker, Staja Q.: 7670
Boon, Jeffrey T.: 3395
Boot, Walter R.: 1040, 1315, 2085, 3025, 7080, 9000, 9010, 9030
Booth, Debbie: 4355
Booth, Sarah L.: 1290
Bordes, Piper J.: 2105, 7550
Borella, Erika: 4185
Borgogna, Joanna-Lynn: 4080
Boris, Lisa M.: 2030
Borrelli, Belinda: 9030
Borrero, Lisa: 2080, 3350, 3430
Borson, Soo: 1265, 7230
Borth, Laura: 1290
Bós, Angelo Jose G.: 3140
Bosak, Emily: 7800
Boscardin, John: 2300
Bose Brill, Seuli: 3345
Bosma, Hans: 1225
Botoseneanu, Anda: 2360, 7300, 7450, 7890, 7990, 9020

Bouchard, Lauren Michele: 1250, 2215
Boucher, Nathan: 4165, 9030, 9530
Boudreau, Robert M.: 5090, 7630
Bouhrara, Mustapha: 3495
Bouldin, Erin D.: 1005, 1280, 1355, 2020, 4365, 7990, 9000
Bouldin, Erin D.: 9010, 9030
Boulton, Aaron J.: 7620
Bourassa, Kyle J.: 1305, 5080
Bouwmeester Stjernetun, Björn: 7000, 7310
Bowblis, John R.: 1115, 9010
Bowen, Holly J.: 9000
Bowen, Lauren M.: 2250
Bowen, Pamela: 9030
Bower, Peter: 4455
Bowers, Barbara: 3180
Bowlby, Douglas: 7390, 7760
Bowles, Kathryn H.: 9410
Bowling, C. Barrett: 9010
Bowman, Caitlin: 7750
Boyd, Cynthia M.: 1035, 9420
Boyle, Julia T.: 9010
Brace, Margaret: 9000, 9410
Brach, Jennifer: 1015, 2155, 9030
Bradbury, Thomas: 4400
Bradley, Dana: 2160, 3000, 3430, 7940, 9000
Bradley, Sarah E.: 7150
Bradshaw, Tancia: 1325
Bradwell, Hannah: 7190
Brady, Samantha: 7090, 9000
Brahma, Arindam: 7840
Brailsford, Jennifer: 9010
Bramajo, Octavio N.: 3425
Brandt, Anthony: 9110
Brandt, Martina: 1220
Brandt, Nicole: 2210
Brannock, M Daniel: 3075
Brasher, Melanie S.: 4375
Bratches, Reed: 9010, 9020
Brates, Danielle: 9020
Bratsberg, Bernt: 9020
Brauer, Simon G.: 2100
Braunstein, Joel B.: 9550
Bravata, Dawn M.: 7130, 7180
Bravo, Adrian: 9010
Bray, Steven: 9020
Brazier, Joan: 9000, 9030
Breckling, Meghan: 7390
Breder, Kelseanne P.: 9100
Brekke, Bailee: 3190, 3455
Bremner, Jordan: 7700
Breneman, Charity: 9000
Brennan, Grace M.: 1305
Brennan-Ing, Mark: 2055, 3565, 5115, 7230, 7810
Brenowitz, Willa D.: 2240
Brenowitz, Willa: 2240
Brent, Michael R.: 5090, 7370, 9010, 9020
Bresslet, Tchelet: 9420
Breton, Kellen: 7170
Brett, Jeanne: 7440
Brewer, Danya: 7210
Brewer, Julie: 2035, 4460
Brewer, Robin: 7680
Brewster, Glenna S.: 2180, 3290, 3535, 7220, 7440
Brewster, Pete: 2295
Bridgforth, James: 4150
Briggs, Emily K.: 7610
Bright, Briana: 7330
Bright, Heather: 9410
Brini, Alberto: 5220
Britt, Katherine Carroll: 3485, 4145
Britton, Karysa: 9020
Britton, Warren: 9020
Brocklebank, Laura: 2015
Brodaty, Henry: 4035
Broderick, Gordon: 9000
Broderick, Vianna: 7150
Brody, Abraham A.: 2070, 2235, 3345, 7230, 9000
Brody, Lilla: 7900
Brohard, Cheryl: 1280
Bromberg, Dan H.: 2055
Bromseth, Janne: 7290
Brooks, Maria M.: 5155
Brosch, Jared R.: 4210, 5105
Brosens, Dorien: 4330
Brotherhood, Kelly: 7160
Brothers, Allyson: 5030
Brotman, Rebecca M.: 4080
Brottman, Barbra: 7070
Brotto, Leticia: 7370
Brotto, Marco: 7370
Broussard, Jia: 1010
Broussard, Jordan: 9010
Broussard, Kimetha: 9020
Brouwers, Mara: 2370
Brown, Candace: 7170
Brown, Casey L.: 4495
Brown, Colette: 5045
Brown, Colleen: 7150
Brown, Diane K.: 4406, 7500
Brown, Ellen L.: 4415
Brown, J. Scott: 7140, 7900, 7950, 9010
Brown, Janine: 9020
Brown, Jessy: 7830
Brown, Jocelyn L.: 5025, 9000
Brown, Justin: 3245
Brown, Karl: 7180
Brown, Katherine: 4266, 7190
Brown, Lana M.: 7160
Brown, Laura J E.: 1130
Brown, Lauren L.: 1000, 3440
Brown, Lisa M.: 2295, 3050
Brown, Maria T.: 1180
Brown, Monique J.: 1085
Brown, Rebecca: 7650, 8900
Brown, Ricardo: 7350
Brown, Sheldon: 9000
Brown, Susan H.: 7640, 7820
Brown, Terrell: 2115
Brown, Todd T.: 3390
Brown IV, Charles H.: 1370
Brown-Hughes, Travonia B.: 7970, 9010
Browning, Wesley R.: 5140
Brubaker, Malaak: 9020
Bruce, Marino A.: 3450
Bruckenthal, Patricia A.: 5030, 7120
Bruine de Bruin, Wändi: 1090
Bruner, Erin: 9010
Bruni, Pietra T.: 4134, 7330
Bruni, Pietra T.: 7340
Brunkert, Thekla: 7160
Brunner, Eric John: 2255
Brunt, Christopher: 1115
Brunton, Laura K.: 7780
Bryan, Rachel: 9010
Bryant, Ashley Leak: 3540
Bryanton, Olive: 3255
Bryk, Kelsey: 2015, 7810
Bubu, Omonigho M.: 7460
Buch, Elana: 2120
Buchanan, Jeffrey A.: 7010, 7590
Buchman, Aron: 9020
Buchman, Emily: 1120
Buck, Harleah G.: 3345, 3395
Buckwalter, Kathleen: 5070, 9020
Bucy, Taylor I.: 1010, 3010, 4140, 5170
Budnick, Andrea: 9000
Budzynska, Katarzyna: 2225
Bueckle, Andreas D.: 4200
Buehler, Susan K.: 4540
Buenger, Rachel E.: 9020
Buerger-Cole, Haley: 3015
Bugbee, Brittany A.: 4408, 7230
Bugos, Jennifer A.: 3420
Bui, Cindy N.: 9510
Bui, Truc Thi Mai: 4385
Bulanda, Jennifer: 7950
Bulat, Tatjana: 7150
Buller-Peralta, Ingrid: 7630
Bullock, Karen: 3345, 4420, 7230
Bültmann, Ute: 4130
Buma, Lise E.: 7120, 7940
Bunker, Jennifer: 4030, 4290
Bunker, Melissa: 9000
Bünning, Farina: 9000
Burack, Orah: 7890, 9010, 9030
Burant, Christopher J.: 2005, 7030, 9020
Burdgorf, Julia G.: 2185, 5160
Burdick, David: 4132, 7480
Burek, Melissa W.: 9000, 9020
Burg, Emily: 2365
Burgei, Alicia: 9030
Burhan, Amer M.: 7080
Burke, Anne E.: 4080
Burke, David T.: 7820
Burke, Shanna: 9000
Burkewitz, Kristopher: 3355
Burkhardt, Summer: 7150
Burnes, David: 1145, 2060, 3375, 4215
Burnett, Jason: 4205, 7610, 9010
Burnette, Denise: 5115
Burnham, Dyanna: 7030
Burns, Edith A.: 4030, 4268, 7680, 9010
Burns, Jeffrey M.: 7990
Burr, Jeffrey A.: 3165, 3350, 4230, 4390, 4435, 7510, 9010, 9530
Burrow, Wesley: 7870
Burrows, Brett: 9010
Bursac, Zoran: 9420
Burton, Lydia M.: 9320
Busby-Whitehead, Jan: 7150
Bush, Julie: 7950
Bussell, Theodore: 2280
Buta, Brian J.: 1370, 5230
Butler, Ryan: 9020
Buto, Peter: 2240
Butte, Sarly: 7010
Buurman, Bianca: 7120
Buxton, Michael R.: 7390
Buxton, Orfeu: 1180, 3500, 4490

Buys, David R.: 7590, 9030
Buys, Katie C.: 9030
Bybee, Sara: 1155, 1250, 9020
Byers, Amy L.: 2280, 4115, 5000
Bygrave, Desiree C.: 7410
Bylund, Carma: 7380, 7580, 7900
Bynum, Julie: 7420
Byrappagari, Divesh: 4300, 7640
Byrd, DeAnnah R.: 7970
Byrne, Nicole: 9000
Byun, Eeeseung: 7280

C

Cabrero Castro, Jose E.: 4395, 9030
Cacchione, Pamela Z.: 7860
Caceres, Maria: 4465
Cadet, Tamara J.: 2260
Cadiz, Ashley D.: 4132, 7500, 9000
Caffrey, Christine: 1095
Cagle, John: 1035, 2020, 4470, 5005, 5015, 9010, 9100
Cahill, Ewan A.: 1245
Cahill, Kevin E.: 3370
Cahill, Sean R.: 5220
Cahuapaza-Gutierrez, Nelson Luis: 9010
Cai, Qian: 5025
Cai, Shubing: 2145
Cai, Yurun: 3510, 5085
Caiado, Camila: 7160
Cain, Cindy: 1075, 2020, 9010, 9100
Cain, Stephen M.: 7820
Caiola, Courtney: 3540
Calamia, Matthew R.: 2105, 7550
Calasanti, Toni M.: 4390
Caldera, Selena: 2395
Calderon, Russell: 3500
Caldwell, Joseph: 3010
Calfee, Kristin R.: 7990
Calisto, Rita: 7320
Calkins, Margaret: 7160
Callaghan, Bridget: 9000
Callaway-Cole, Larissa: 2390
Calloway, Shayla: 4100
Calo, William: 5145
Calvani, Riccardo: 9020
Calver, Jen: 9010
Calvo, Rocio: 4420
Camacho, David: 5115, 9010
Cameron, Judy L.: 9020
Cammer, Allison: 1200
Camp, Cameron J.: 3365
Campbell, Kenna: 9010, 9540
Campbell, Kimberly C.: 3260
Campbell, Laura: 4340
Campbell, Noll L.: 7080
Campetti, Randi: 2030
Campione, Joanne R.: 2375, 7780
Campitelli, Anthony: 2015, 7810
Campos, Fernando A.: 1195
Campos, Jennifer: 3270
Can, Reyyan: 4180
Candon, Molly: 8900
Canell, Anastasia E.: 7980, 9020
Canham, Sarah L.: 1155, 1205, 7780
Cannon, Jacqui: 9000
Cannon, Melissa L.: 4408, 7700
Cantu, Phillip: 3465, 7600, 9020, 9030, 9560
Cao, Feifan: 9020
Cao, Jiawei: 9510
Cao, Katy (Qiuchang): 1065, 2045, 3095
Cao, Yanpei: 2265
Cao, Yuanyuan: 3085, 3295
Cao, Yumeng: 9000
Capolino, Alexandria C.: 4375
Cappola, Anne R.: 4080
Cappon, Davide: 2155, 9030
Caprio, Thomas V.: 3285
Capuano, Ana W.: 2120
Caraballo, Christina: 3175
Carbone, Elena: 4185
Carden, Keisha D.: 1365
Cardenas, Cynthia: 2160
Cardenas, Valeria L.: 7260
Carder, Paula: 1210, 2105, 3220, 3320, 7550
Carey, Cyriah: 9020
Carey, James: 1195
Carlson, Bryant: 2105, 3220
Carlson, Chalise: 4095
Carlson, Michelle: 1015, 2155, 3390, 3405, 4145, 7650, 9000
Carmichael, Pierre-Hugues: 7030
Carney, Maria Torroella: 1020, 7260
Carpenter, Brian D.: 1365, 4210, 4406, 7000, 7550, 7900, 9020
Carpenter, Joan: 3395, 4005, 9010
Carpenter, Kristen: 7410
Carpenter, Nancy: 4460
Carpenter, Rebekah: 2045, 3095
Carr, David B.: 9030, 9330
Carr, Dawn C.: 1230, 2045, 3095, 7030, 7510, 9010
Carr, Deborah: 1305, 2105
Carranza, Maria: 2270
Carratu, Kevin: 7260
Carrión, Carmen: 1305
Carrico, Catherine: 7610, 7840
Carrion, Iraida V.: 2260, 2330
Carrion-Baralt, Jose R.: 7620
Carroll, Brooke: 7060
Carroll, Judith E.: 1085, 2285, 5225
Carson, Erin: 9530
Carson, Paul: 7710, 7810
Carstensen, Laura Lee: 1320, 4480, 4495, 7020, 8900
Carter, Jammie: 9520
Carter, Kara: 3210, 4050
Carter, Rachel M.: 1335
Carvalho do Nascimento, Paulo Roberto: 4268, 7680
Casanova, Giuliana: 2215, 4405
Casanova, Ramon: 7630, 9580
Case, Donna: 3570
Case, Rebekah: 7410
Caserta, Mike: 1150
Casey, James: 4495
Caskie, Grace IL.: 3250, 7590, 7980
Caspi, Avshalom: 1305, 5080, 7660
Cassatt, Sarah: 9010
Cassidy, Jessica L.: 2330, 2390, 4420, 7910, 9000, 9020
Castegren, Markus: 9030
Castillo-Azofeifa, David: 3070
Castle, Nicholas: 3165
Castro, Eunice: 7460
Castro, Saul: 7950
Cataldo, Amelia: 7410
Catt, Wade: 9020
Caudillo-Ortega, Lucia: 2290
Cauley, Jane A.: 4260, 5085, 7650
Causey, Shakiera T.: 9100
Cavalli, Stefano: 3130
Cavanaugh, Robert: 9430
Cave, Nkosi: 7620
Cawthon, Peggy M.: 2320, 3475, 3500, 4260, 5085, 5210, 7650
Cazumbá, Aryane S.: 7920
Cedars, Marcelle: 7100
Celso, Brian: 7860
Cenko, Erta: 9000
Censky, Dina: 9520
Cenzer, Irena: 4205
Cerino, Eric S.: 1360, 3230, 3545, 5080, 9000
Cerino, Lauren: 1175, 3090
Cerqueira, Margarida: 4405
Ceruso, Marco: 1315
Cervantes, Diana: 4220
Cervantes, Maria: 7180, 9000
Céspedes, Amarilis: 3415
Cha, Eun Gyo: 9540
Cha, Hyungmin: 9000, 9020
Chabala, Peg: 4315
Chacko, Thomas P.: 9000
Chai, Emily: 7860
Chai, Hye Won: 4370
Chakravarthy, Ameera A.: 7170
Chakravarty, Esha: 7200, 7360
Chakravarty, Indrani: 7200
Challa, Suman: 7480
Chamberlain, Stephanie A.: 4025
Chamberlin, Elizabeth S.: 7850
Chambers, Yolima: 1150, 3240
Chan, Alex Siu Wing: 7050
Chan, Andrew: 9000, 9020
Chan, Angelique: 2230, 3435, 4020, 4175
Chan, Athena Chung Yin: 3060, 4520
Chan, Ding-Cheng (Derrick): 4134, 7830, 7930
Chan, Helen Yue-lai: 4540
Chan, Kiera: 1375
Chan, On Fung: 3115, 4255
Chan Carusone, Soo: 4340
Chandler, Kelly D.: 4045, 7090
Chandra, Meghana: 5220
Chang, Angela: 9030
Chang, Catie: 1215
Chang, Chu-Hsiang: 9000
Chang, Chung-Chou H.: 5080

Chang, E-Shien: 1145, 2030, 2060
Chang, Jun Ha: 7510, 7620
Chang, Ling-Hui: 7780
Chang, Mei-Ching: 9030
Chang, Pei-Shiun: 9400
Chang, Yuanqing: 1245
Chang, Yuefang: 5225
Chang, Yu-Ping: 5230, 7260, 7330, 7360, 7620, 9010, 9020
Chao, Linda L.: 1040, 2035, 4035
Chao, Pei-Shu: 7950
Chao, Shiau Fang: 7030, 7960
Chao, Ying-Yu: 5230, 7360
Chapela, Sofia: 7770
Chapman, Brian: 4035
Chapman, Cara: 5200
Chapman, Hannah: 4515
Chapman, Joyce: 2355
Chapman, Steve: 7380
Chappell, Richard J.: 2240
Char, Adarsh: 3235
Charles, Doreek: 1240
Charles, Susan: 1105, 3135, 3195, 3230, 5045, 9020
Charness, Neil: 1315, 9000, 9010, 9030
Chasteen, Alison L.: 1200
Chatterjee, Samir: 7840
Chaturvedi, Ritika: 4385
Chaudhari, Diptaraj S.: 7100, 7870
Chaudhari, Nikhil: 9020, 9030
Chaudhury, Habib: 1145, 2340
Chavez, Arlette ": 2085
Chavez, Hector J.: 8900
Chavez, Margeaux: 7150
Che, Yingni: 7370
Chee, Wonshik: 7220
Cheeks, Jodi: 7860
Chehabeddine, Lara: 9500
Chek, Carmen Jia-Wen: 7970
Chellappa, Karthikeya: 9020
Chen, Allison: 3270
Chen, Bangwei: 9000
Chen, Chen: 2280
Chen, Chixiang: 3550, 4115, 9000
Chen, Cynthia: 3440, 4385
Chen, Diefei: 3335, 3510, 4265, 7870
Chen, Duan-Rung: 7260
Chen, Enna: 4495
Chen, Haiying: 4355, 5230
Chen, Hao-Min: 3400
Chen, Honglei: 2240
Chen, Jiachen: 7640, 7830, 9000
Chen, Jia-Jen: 3400
Chen, Jiayu: 9010
Chen, Jie: 7370
Chen, Jie: 7460
Chen, Jiu-Chiuan: 2320
Chen, John S.: 9000
Chen, Kao Chin: 9010
Chen, Kuan-Hua: 4495
Chen, Kuan-Hua: 4495
Chen, Kuan-Hua: 4495
Chen, Lei: 3210, 5215
Chen, Li-Mei: 9000
Chen, Lin Yee: 3390
Chen, Lingming: 1350, 2385, 3155
Chen, Lizhen: 8900
Chen, Long: 1260, 7120
Chen, Lulu: 7680
Chen, Min-Chia: 4155
Chen, Ming-Hui: 7460
Chen, Nai-Wei: 1180
Chen, Po-Hung: 7390
Chen, Qi: 7650
Chen, Qize: 2045, 3095
Chen, Ruijia: 2300
Chen, Shanquan: 1165
Chen, Shyh-Huei: 3505
Chen, Siting: 7300, 7540
Chen, Sunny: 9030
Chen, Szu-Wei: 7780
Chen, Tuo-Yu: 4490
Chen, Wei-An: 9010
Chen, Will: 9030
Chen, Xi: 1045
Chen, Xi: 1165, 2225, 2300, 3230, 3450
Chen, Xi: 5225
Chen, Xiaodong: 9000, 9020
Chen, Xiaoyuan: 7050
Chen, Xiayu Summer: 7080
Chen, Xirun: 4120
Chen, Ya-Mei: 7780
Chen, Yanjun: 2240
Chen, Yi-An: 1120
Chen, Yingtong: 7850
Chen, Yizhou: 9110
Chen, Yu-Chih: 2160, 4305
Chen, Yu-Ming: 1125, 4310, 7930
Chen, Yung-Ming: 7930
Chen, Yuntao: 2255
Chen, Yun-Zih: 7290
Chen, Yuqing: 5155
Chen, Yuxin: 4155
Chen, Yuxuan: 1305
Chen, Zhirui: 7550
Chen, Ziyuan: 9020
Cheng, Catherine J.: 4475
Cheng, Clio Yuen Man: 3515
Cheng, David: 9000
Cheng, Evien: 7370
Cheng, Grand: 9010
Cheng, Greta Jianjia: 3405
Cheng, Huai Y.: 7150, 7310
Cheng, Jie: 1350, 3155
Cheng, Ling Ling: 9000
Cheng, Mengling: 2285, 7930
Cheng, Sheung Tak: 1140, 1145
Cheng, Yan: 3490
Cheng, Yiping: 9020
Cheng, Zijing (Flora): 4140
Cheong, Josepha: 7140
Chepkorir, Joyline: 5175
Cherry, Barbara J.: 7070
Cherry, Katie E.: 2105, 7550
Chesak, Sherry: 7420
Cheshire-Allen, Maria: 1335
Cheslock, Megan: 9410
Chesney, Margaret A.: 2035, 4035
Chester, Mia Recha: 2180
Cheung, Dominique: 9000
Cheung, Ethan Siu Leung: 3305, 7230, 7270
Cheung, Kin: 7040
Cheung, Pui Yin: 9020
Cheung, Ying: 4310
Chew-Graham, Carolyn: 9010
Chi, Iris: 1185, 3005
Chi, Nai-Ching: 4134, 7620
Chi, Peggy: 9550
Chi, Vivienne: 5105
Chia, Arthur: 3435
Chia, Chee: 4490
Chiang, Hao-Ping: 9020
Chiang-Hanisko, Lenny: 5175
Chiareli, Alexandra: 3260
Chidebe, Runcie CW.: 2010, 2375, 4130, 4345, 5110, 7040, 7380, 9010
Chien, Shao-Yun: 7300, 7910, 9010, 9020
Chihuri, Stanford: 9010, 9020
Childs, Elisa M.: 7360
Childs, Ellen: 7810
Chiles, Kira: 7170
Chim, Kannie: 4408, 7700
Chin, Ching-I.: 9030
Chin, Janecca: 1340
Chin, Jessie: 7590
Chin, Kristi: 2100
Chipalo, Edson: 4510
Chipperfield, Judith G.: 9430
Chiriboga, David: 7510
Chisholm, Latarsha: 9030
Chisolm, Theresa: 1295
Chiu, Ching-Ju: 7610, 7940, 9010, 9030
Chiu, Wan-Yu: 7780
Chiu, Yi-Chen: 4155
Chiu, Yu-wen: 7410
Chladek, Jason S.: 9030
Chmelo Kemp, Elizabeth: 3505, 4350
Cho, Catherine: 9010
Cho, Chi: 7610, 7640
Cho, Christine: 1215
Cho, Emma: 9030
Cho, Eunae: 9010
Cho, Eunbyul: 9100
Cho, Eunhee: 7420, 7460, 7660, 7720, 7760
Cho, Hannah: 7980, 9000
Cho, Hyeonji: 7030, 7380
Cho, Hyun Jin: 7550, 9020
Cho, Jeff: 9030
Cho, Jinmyoung: 7400
Cho, Joonyoung: 1170, 4325
Cho, Jungwon: 7420, 7760
Cho, Kyoungmin: 5045
Cho, KyungAh: 7850
Cho, Sangmi: 7780
Cho, Soyeon: 2090
Cho, Tsai-Chin: 2280, 7320
Cho, Youmin: 7570
Cho, Youngmin: 3540, 4380
Cho, Youngmin: 7780
Chocninov, Harvey M.: 7380
Chodosh, Joshua: 1265, 7130, 7840
Choe, Ian: 4075
Choi, Bomi: 7040, 7080, 7610
Choi, Eunyoung: 9020, 9310
Choi, Gayoung: 9030, 9570
Choi, HwaJung: 1340, 7320, 9030
Choi, Hyojin: 7590
Choi, Jean: 4425
Choi, JiYeon: 7080, 7900, 7960, 9020
Choi, Jung-Yeon: 9000
Choi, Mi Sun: 7780
Choi, Mona: 7080
Choi, Moon: 1260, 7690, 9030
Choi, Namkee G.: 4375, 4460, 7270, 7610
Choi, Seongmi: 7080, 9020

Choi, Shinae L.: 1305
Choi, Soyoung: 2290
Choi, Won: 1320, 4205
Choi, Y.Joon: 7220
Choi, Yeon Jin: 1165, 7720
Choi, Yong K.: 3205, 5055, 7460
Choi, Yoonseok: 4480
Choi, Young: 9100
Choi-Allum, Lona H.: 3095, 5040, 5175
Chok, Jay: 1095
Choma, Elizabeth A.: 5100, 9410
Chon, Myoung-Gi: 9010
Chong, Natalie: 3010
Chopik, William J.: 4145, 4325, 4370
Choquette, Cassidy: 9010
Chou, Kee Lee: 1065, 7610, 7930
Chou, Yi-Chun: 4134, 7830
Choudhury, Renoa: 5075, 7150, 7820, 9030
Choukas, Nathaniel R.: 4460
Choula, Rita B.: 1155, 2395
Choun, Soyoung: 1160, 7510
Chow, Denise: 4010
Chow, Stephanie: 4266, 7190, 7820
Chowdhury, Nahian: 9020, 9030
Choy, Jacky C.P.: 5235, 7420
Christensen, Kaare: 1195, 4260, 5090, 9010, 9020
Christian, Thomas: 3230, 4195
Christiansen, Mats: 7360
Christianson, Tracy: 7700
Chrzanowski, Brandi L.: 4406, 7500
Chu, Charlene H.: 3255, 4245, 4405, 7190, 7680
Chu, Li: 1320, 4480, 8900
Chu, Winnie: 4268, 7680
Chua, Denise Qian Ling: 7760
Chui, Cheryl: 3115, 3515, 4255
Chui, Geoffrey: 1095
Chui, Michelle A.: 7080, 9030
Chukwuorji, JohnBosco: 9000
Chung, Edwin K H.: 3280
Chung, Jane: 3025, 7400
Chung, Seok Jong: 7400
Chung, Soondool: 7030, 7510, 7590, 7890
Chung, Tim: 4406, 7000, 9020
Chungkham, Holendro Singh: 3265
Chunyu Liu, Chunyu Liu: 7100
Church, Kimberly: 7860
Churchill, Linda C.: 1350, 2385, 3155
Chusyd, Daniella E.: 1060, 9020
Cichy, Kelly E.: 3545, 4425
Cidav, Tom: 5100, 7620
Cigolle, Christine: 9000
Ciolfi, Mary Lou: 3175
Cipher, Daisha M.: 3305
Ciro, Carrie: 9030
Clair, Catherine A.: 2290, 3240
Clancy, Olivia: 9010
Clapés Pemau, Raquel: 9020
Claproth, Alycia: 1285
Clare, Linda: 4070
Clark, Alexandra L.: 9210
Clark, Amy G.: 7150
Clark, David: 9010
Clark, Janet M.: 1375
Clark, Kirsty A.: 1180
Clark, Lindsay: 9020
Clarke, Anthony: 1350
Clarke, Philippa: 1170
Classen, Sherrilene: 1030
Clauser, Steve: 9000
Clay, Danielle: 7480
Clay, Olivio J.: 7020, 7060, 7090, 9000
Clayton, Jordana L.: 1150, 2035, 9020
Clayton-Jones, Mary C.: 9020
Clem, Sarah: 5005, 9010
Clevenger, Carolyn K.: 2070, 2300, 7440, 9020
Clifford, Kristen: 3055
Clifton, Jennifer: 7480
Cline, J. Mark: 7370
Cloud, Leslie: 9330
Clouston, Sean AP.: 2230, 7590
Cloyes, Kristin G.: 7470, 7540
Clutton, Jonathan: 7990
Coates, Cameron: 5070
Coates, Martha C.: 4005
Coblenz, Clara W.: 7510
Coco-Ripp, Jo Ann: 4350
Coe, Antoinette B.: 9020
Coe, Norma B.: 1115, 4365
Coen, Paul M.: 5210
Cofield, Stacey: 7360
Cogdill-Richardson, Kiana: 1390
Coghlan, Leah: 8900
Cohanim, Niki: 4420
Cohen, Alan A.: 1110
Cohen, Ann D.: 9500
Cohen, Jonathan E.: 1020, 2175
Cohen, Matthew L.: 7620
Cohen, Pinchas: 2175
Cohen, Steven A.: 4025
Cohen, Trevor: 7300
Cohn-Schwartz, Ella: 4125, 9030
Colón-Emeric, Cathleen: 7300, 7410
Colburn, Jessica: 4170
Coldiron, Allyson M.: 7080, 7590, 7900
Cole, Steve: 1215, 2325
Coleman, Carissa: 2160, 3205, 3335, 7300
Coletta, Giulia: 5100
Coley, Rebecca Yates: 3490
Collins, Stacey: 2325
Collins-Pisano, Caroline: 4445
Colon, Anaeliz: 7770
Colon-Emeric, Cathleen: 1370, 4485
Colvin, Alicia B.: 5155
Comnick, Carissa: 5225
Conard, Ryan: 7020
Condo, Angela: 1375
Condon, Shelley E.: 7460
Cone, Nicholas: 1055
Coney, Rachel: 7620
Cong, Xiaomei: 7460
Cong, Zhen: 7550
Coniff, Joshua: 5225
Connelly, Denise: 5105, 7160, 7780
Conner, Kyaien: 3560
Conniff, Joshua: 4132, 7480
Connolly, M.T.: 3375, 4215
Connor, Lisa Tabor: 1255
Conover, Laura: 7330
Conrow, Elizabeth: 1310
Conroy, David E.: 4310, 9010, 9020, 9030
Consolandi, Monica: 7070, 7600
Consortium, LifeGEIST.: 9020
Convery, Kimberly: 7230
Conway, Cathy: 1280
Cook, Allyson: 7660
Cook, Maxwell: 9020
Cook, Miranda: 9010, 9310
Cook, Penny: 3175
Cook, Robert L.: 1030
Cook Maher, Amanda: 4325
Cooke, Paul S.: 1380, 7370
Coon, David W.: 5075, 7970
Cooney, Alissa: 9010
Cooper, Charles: 7620
Cooper, Dayna: 2145
Cooper, Hannah LF.: 3565
Cooper, Kalvry A.: 7080
Cooper, Leonie A N.: 7190
Cooper, Lujira J.: 1250, 5150
Corazzini, Kirsten: 3145, 5020
Corbett, Duane: 9580
Corbett, Kyle: 4280
Corcos, Daniel: 4310
Cordell, Ashlee: 7060, 7460
Coresh, Josef: 2365, 3510, 7110
Corle, Melanie: 9020
Corley, Samantha S.: 4045
Corneau, Emily: 7160
Cornelison, Laci: 3175
Cornelius, Talea: 2035
Cornell, Portia: 3320, 7160, 9030
Cornwell, Hannah E.: 7900
Correa, Arlene: 3565, 7810
Corrigan, Anne E.: 3405
Cortés-Ortiz, Mónica: 9010
Corwin, Logan: 7210
Coschigano, Kat E.: 7830
Cosentino, Stephanie: 1040, 7830
Costa, Amy N.: 3420, 4540, 7080
Cothran, Fawn: 3030
Cotten, Shelia R.: 3025
Cotter, Valerie T.: 1075, 4170
Cotton, Cecilia J.: 4280
Cotton, Jesse J.: 9010
Cotton, Quinton D.: 1150, 2130, 3240
Cotton, Sam: 4040, 4360
Couch, Elyse: 7620, 7720
Coughlin, Joseph F.: 1175, 3090, 7090, 7910, 9000, 9010
Coulourides Kogan, Alexis M.: 3220
Couve, Amanda: 4040
Covington, Amy: 9000
Cowan, Linda J.: 7150
Cox, Brian S.: 7390, 7460
Cox, Elizabeth: 7770
Cox, Krista M.: 5190, 7680
Cox, Laura: 9000
Cox, Polly Madson: 4215
Cox Sullivan, Sheila: 7160

Coyle, Caitlin: 4150
Coyle, Caitlyn E.: 9510
Coyle, Marissa: 9000
Craggs, Jason G.: 4490
Craig, Dawn: 4455
Crampton, Alexandra: 4325
Crane, Breanna: 1015, 2155, 3405, 4145, 9000
Cranford, James: 7390
Crary, Kate: 5020
Crawford, Nicole O.: 3560, 4010
Crawford, Sybil L.: 5155
Cready, Cynthia: 5185
Crenshaw, Kiresten: 9010
Crettenand Pecorini, Béatrice: 2390
Crews, Deidra C.: 1345
Crimmins, Eileen M.: 1125, 1320, 1325, 2325, 3465, 4395, 7380, 7540, 7890
Crisci, Alex: 9010
Crittenden, Jennifer: 2020, 3075
Crocco, Elizabeth A.: 9010
Croff, Raina: 4100
Cronin, Cory E.: 7780
Cropper, Avareena: 5220
Crosby, Paris: 7510
Cross, Dori: 1010
Crossdale, Rachel: 4450
Crouch, Adele: 7420
Crouter, Scott E.: 2385, 3155
Crowder, Sylvia: 7410
Crowder, Victoria R.: 4380
Crowe, Michael: 3050, 9000
Crowley, D. Max: 7950
Cruitt, Patrick J.: 3520
Cruz Madrid, Katya Y.: 4465
Cruz-Almeida, Yenisel: 4134, 7330, 7630, 9010
Cruz-Carrillo, Yesenia: 1360
Cruz-Oliver, Dulce Maria: 7360
Csikai, Ellen L.: 7380
Cudjoe, Thomas: 4030, 4145, 5160, 9030, 9420
Cuevas, Heather E.: 7840, 7980
Cui, Jialin: 9510
Cui, Xiaoyan: 9020, 9300
Culberson, John W.: 7160
Culligan, Joanna RS.: 9000
Cummings, Greta: 4408, 7720
Cummings, Jeffrey: 9020
Cummings, Michael: 7100
Cummings, Steven R.: 3475, 3500, 5210
Cummins, Phyllis: 2375, 4130, 4345, 5110, 7040, 7380
Cundiff, Jeanne: 9210
Curbeam, Deanna: 7300
Curiel, Rosie: 9010
Curl, Angela L.: 5175
Curley, Taylor M.: 7510
Curran, Sean: 9020
Currie, Leanne: 9030
Curriero, Frank C.: 3405
Curry, Natasha: 2255
Curtis, Ashley F.: 3420, 4490, 4540, 7080
Curtis, David: 9020
Curtis, Elizabeth Heaton: 1310
Curtis, Megan E.: 9010
Curtis, Sarah: 1015
Curyto, Kimberly J.: 4290
Cutchin, Malcolm: 2340
Cutforth, Asia: 3410, 5200
Cvejkus, Ryan K.: 5090, 7830, 9020
Czaja, Sara: 1145, 1315, 2050, 2060, 3170, 3515, 4268, 7360, 7680
Czyz, Rebecca: 9010

D

Dabelko-Schoeny, Holly: 1095, 1205, 1330, 2130, 3045, 4305
Dabrowski, Barbara S.: 7610, 7840
Dacks, Penny A.: 7420
Dadgostar, Porooshat: 2145
Dadson, Deborah A.: 7990
Dag, Dominick: 7100
Dagne, Mahederemariam: 9010
Dagum, Paul: 9000, 9010, 9020
Dahal, Poshan: 7030
Dahl Aslan, Anna K.: 7130
Dahlgren, David: 2340, 7380
Dalkin, Sonia: 2375
Dallo, Florence: 3440
Dalpe, Sara: 7070
Dalton, Jarrod: 1170
D'Ambrosio, Lisa A.: 7910, 9000, 9010
D'Amore, Cassandra H.: 7170
Dampf, Hana: 4280
Dams-O'Connor, Kristen: 7650
Damush, Teresa M.: 7130, 7180
Dang, Huong: 9530
Dang, Linh: 3085
Dang, Linh: 3380
Dang, Linh: 4545, 5010, 5050
Dang, Patrick: 2085
Dang, Stuti: 1005, 9010, 9030, 9420
Daniel, George: 9020
Daniel, Kathryn M.: 2390, 3305
Daniel, Nikita R.: 4425
Daniels, Ramon: 9010
Danielson, Jodi: 2095
Danilovich, Margaret: 4310, 9020
Danilovich, Margaret: 9030, 9530
Danschin, Arlene: 7440
D'Antonio, Patricia M.: 1285, 2165, 3315, 3455
Daquin, Jane C.: 4415
da Rosa, Grace: 7500
Darby, Timothy: 1125
Darcy, Derek: 2145
Darmadi Blackberry, Irene: 2260, 9010
Darvishpoor Kakhki, Ali: 7660
Da Silva, Jaqueline: 7920
Darwish, Hala: 1355, 9000
Das, Ayushi: 4120
Das, Jayanta K.: 9020
Das, Phebe: 3340
Das, Sanchari: 3075, 7520
Das Bhattacharjee, Sreyasee: 9010
Das Gupta, Debasree: 3220, 7210
Dasanayake, Thakshila: 3020
Dasgupta, Abhirupta: 5030
Dash, Kim: 2030
Das-Munshi, Jayati: 4510
Dassel, Kara Bottiggi: 1150, 2035, 9020
Datcher, Ivree: 1010, 3435
Dauenhauer, Jason: 9010
Daugherty, Ana M.: 7510, 9010
Daum, Christine H.: 1165, 1280, 5015
Dave, Chintan: 5230, 7160
Davey, Adam: 3530, 9030
Davey-Rothwell, Melissa: 4460
David, Daniel: 2070, 2235
Davidson, Patricia M.: 7770, 7840, 7960
Davidsson, Rebecca: 7400
Davie, Laura: 5020
Davies, Abigail: 1335
Davies, Megan: 3180
Davies, Nathan: 4440, 4470
Davila, Heather: 2180, 9020, 9520
Davis, Darlene: 2295
Davis, Deborah W.: 7370
Davis, Derik L.: 7130, 9000
Davis, Elsa: 7060
Davis, Ian: 4355
Davis, Jamaine: 1380
Davis, Jennifer C.: 5100
Davis, Rebecca: 7800
Davis, Tracy EK.: 9030
Davison, Carrie Ann: 7540
Davitt, Joan: 2020, 5025, 9010, 9100
Davlyatov, Gani: 2350
Davlyatov, Ganisher: 2350, 3365, 4190
Davoudi, Anis: 2150, 3390, 7650
Davy, Philip: 2380
Daw, Warwick E.: 5090
Dawson, Bonnie D.: 7180
Dawson, Catherine MP.: 7770
Dawson, Walter: 7770, 9010
Dayama, Neeraj: 2350
Dayrit, Jessika G.: 7460
Dayyani, Faranak: 7680
De, Supriyo: 2195
de Boer, Bram: 2370
de Cabo, Rafael: 2065, 3495, 3575
De Carvalho, Carlos Roberto Ribeiro: 9010
De Carvalho, Celso Ricardo Fernandes: 9010
De Donder, Liesbeth: 1065, 4330, 7280
De Fries, Carson M.: 2090, 2330, 4195, 5200, 7230, 9010
De Godoy, Caroline G.: 9010
De Main, Atami: 5115
de Man-van Ginkel, Janneke M M.: 2280, 3340
de Mederios, Kate: 3200
De Melo, Daniella: 9020
de Munck, Isabel M.: 1370
de Oliveira Silva, Alessandro: 7150, 7340
de Rosa, Cristina: 7330
de Souto Barreto, Philipe: 2320
de Souza, Yolanda A.: 3140
Deac, Alexandra: 2055
Deal, Jennifer: 1295, 2135, 2240, 2365, 3335, 3390, 3510, 4110, 4265, 4266, 7690

Dean, Doug: 3495
Dean, Meagan: 7670
Dean, Olivia: 5175
Deason, Rebecca: 7420
Debergue, Patricia: 9020
deCardi Hladek, Melissa: 3240, 4060
DeCaro, Jason A.: 9210
DeCian, Enrica: 2105
Decker, Autumn: 1240, 1365, 3250
Decker, Hannah: 5200
DeFontes, Clara: 9020
Degen, Nora M.: 3100
Degenholtz, Howard B.: 3175, 4190, 7690, 9010, 9540
DeGennaro, Jennifer: 1130, 7290
Degraves, Brittany S.: 4515
DeGroot, Lyndsay: 7770
Deindl, Christian: 4275
DeJohn, Amber: 9000
Dekle, Kori: 7830
Delacourt, Barbara: 7680
Delgado, Daphne: 1265
Delgado, Ferdinand: 9030
Delgado-Ortiz, Laura: 3550
Delgado-Velandía, Mario: 2320
DeLiema, Marguerite: 3075, 4215
DeLiema, Marti: 3090, 3460
Delk, Michelle: 3435
Deller, Juergen: 7290
DeLongis, Anita: 3135, 3520, 7550
Deloria, Rochelle: 9420
DeLucia, Jack: 9000
Demalia, Angelo: 9030
Demir, Ibrahim: 4134, 7620
Demiris, George: 4415, 7460, 7680, 9000, 9030, 9540
DeMott, Andrew: 7710
Demuth, Ilja: 1060
Deng, Chenxin: 9000
Deng, ZhiDi: 1350
Denham, Grant: 7100
Dennis, Brandon C.: 9000
Dennison Himmelfarb, Cheryl R.: 7770, 7840, 7960
Denno, Ben: 3020
Denny, Bryan: 9110
Denson, Kathryn: 7310
Dent, Elsa: 7660
Dentchev, Alexander: 9010
Dentino, Andrew: 7130
DePasquale, Nicole: 7620
Deputy, Amanda: 4350
Der Ananian, Cheryl: 9030
Derby, Carol A.: 3500, 4490, 5155, 9020
Deshmukh, Madhyami: 7950
Desir, Marianne: 9420
Desjardins, Michael R.: 1015, 3405
Desrochers, Madeleine: 7010
DesRoches, Catherine: 9000
Devi, Reena: 2375, 7720
Devin, Michael: 7680
Devkota, Rashmi: 4280
DeVone, Frank: 7160
Devos, Hannes: 9330
Dewan, Neha: 4515
Dewji, Aleena: 4290
Dey, Amit: 5060, 7880
Dey, Judith: 2375
Dey, Nutifafa E Y.: 7610
Dezine, Marie L.: 7500
Dhakal, Usha: 9010
Dhar, Sohini: 7480
Dharangutte, Prathamesh: 7680
Di, Chongzhi: 3390
Di Bona, Vito: 7620
Di Gessa, Giorgio: 4015, 4275
Diaz, Jose A.: 4475, 5045
Diaz, Miranda H.: 3365
Diaz-Ramirez, Grisell: 2300
DiBartolo, Mary C.: 7000
DiCarlo, Nicholas: 5150
DiCerbo, Loraine: 7510
Dickerson, Anne E.: 7690, 7730
Dickerson, Bradford: 2035
Dickerson, Kimberly: 3110
Dickin, Clark: 7340
Dickinson, Stephanie: 9020
Dickson, Chelsea: 9010, 9540
Dieciuc, Michael A.: 1040
Diederich, Freya: 1115, 3530
Diefenbach, Michael: 4030
Diehl, Christin: 9000
Diehl, Cody L.: 7810
Diehl, Manfred: 2105
Diener, Christian: 9020
Diener-West, Marie: 9560
Dieppa, Emily: 4165
Dierckx, Eva: 1065, 7280
Dietrich, Mary S.: 9020
DiGirolamo, Marissa: 3195
Digman, Leanne: 9410
Dignam, Ritchell: 7360
Diji, Abigail Kusi-Amponsah: 7790
Dikhtyar, Oksana: 3190
Dikken, Jeroen: 4125
Dikmans, Bas: 4330
Dilanchian, Andrew: 1040, 9010
Dill, Janette: 7230
Dillon, Ellis C.: 1240, 3525
Dillon, Kaitlyn E.: 4340
Dillon, Kristen: 9020
DiMaria-Ghalili, Rose Ann: 3395, 4005, 5130, 9440
Ding, Huitong: 9010
Ding, Jingzhong: 4355
Ding, Jun: 5085
Ding, Kedong: 7260
Dionne, Laura: 4290, 7020
Dionne-Odom, J. Nicholas: 7680
DiScala, Sandra: 7840
Dishaw, Mary: 3480, 9000
Distefano, Giovanna: 5210
Dixit, Vishwa Deep: 2065
Dixon, Dave: 9010
Dixon, Josie: 4440
Dixon, Steven: 9020, 9200
Doan, Jared: 7820
Dobalian, Aram: 2295
Dobbs, Debra: 2000, 2245, 3165, 3345, 5170, 9030
Dodge, Shana: 7420
Doherty, Elaine: 4408, 7230
Doherty Bea, Megan: 5050
Dolamore, Michael J.: 9000
Dolan, Hanne R.: 5075
Dolansky, Mary A.: 4525, 7590
Dolbin-MacNab, Megan L.: 7950
Dolsen, Emily: 2280
Domeracki, Alexis: 9020
Dominguez, Rachael: 7590
Don, Janith: 5180
Donahue, Patrick T.: 1015, 3390, 3405, 7650, 9000
Donath, Carolin: 2370
Donato-Woodger, Simon: 7190
Donega, Stefano: 2195
Donehower, Allison K.: 4415
Donelan, Karen: 4430
Dong, Birong: 7830
Dong, Fanghong: 3485
Dong, Fanghong: 3490
Dong, Fanghong: 4134, 7330
Dong, Wuyi: 7610, 9030
Dong, Yanbin: 9010, 9500
Dong, Yu: 7330
Donkers, Hanneke: 3340
Donkor, Peter: 7790
Donnelly, John: 2295
Donnelly, Rachael: 7300
Donohoe, Maria: 3445, 3540, 7440, 7470
Donorfio, Laura KM.: 1080, 2095, 3325, 3350, 4035
Donovan, Heidi: 3030, 7530, 9010
Doolittle, Benjamin: 4145
Doose, Michelle: 3210
Doran, Kelly: 7170
Doran, Patty: 1385, 3260
Dore, Emily: 4045
Dorisca, Eugenia: 7000
Dorr, David: 2360, 7450
Dorris, Jennie L.: 7300
Dorris, Joseph: 9020
Dorsey, Kirsten: 7150
Dosa, David M.: 2295, 5170
Dougherty, Ryan J.: 2150, 3390
Douglas, Joy: 7620, 9000, 9020
Douglas, Natalie: 5030
Douglas, Vanja: 1380
Douma, Gebrich: 3340
Dowden, Lauren: 7700
Dowling, Maritza: 1070, 5225, 7100, 7330
Downer, Brian G.: 4395, 9030, 9310, 9560
Downey, Payton A.: 4268, 7680
Doyle, Cassidy: 9000
Doyle, Margaret F.: 7640, 7830, 9000
Dozier, Mary E.: 2040
Dragomir, Gerry: 3255
Drake, Jonathan D.: 9550
Drake, Judy: 9000
Drazich, Brittany F.: 2305
Drazon, Molly: 9400
Dreer, Laura E.: 7650
Driesse, Tiffany: 4268, 7680
Driscoll, Laura: 9000
Driver, Jane: 4310
Drolet, Marie-Josee: 9030
Drost, Jennifer: 7250, 7480
Droste, Andrew P.: 7820
Dryden, Eileen: 9010
D'Souza, Clive: 7750
D'Souza, Gail: 5145
Du, Pang: 5195
Du, Yan: 5055, 7120, 7660, 9030
Duan, Hongzhe: 2015, 7110, 7370, 7800
Duan, Liping: 7870
Duan, Yinfei: 4408, 4515, 7720
Duangjina, Thitinan: 4025
Duara, Ranjan: 2180, 5070

Duarte, Andres: 2180, 5070
Dubé, Catherine: 7680
Duberstein, Paul R.: 1240, 4105, 4305
Dublin, Sascha: 3490
Dubois, Sacha: 3570
DuBois, Samantha: 9030
Dubowitz, Tamara: 3405, 9010
Dubuc, Jade: 7680
Duchowny, Kate: 3225, 9010
Duck, Angela: 1070
Duff, Kevin: 7620
Duffy, Evelyn G.: 4525
Dugan, Elizabeth: 4266, 7230, 7270, 7830, 9530
Dugan, Sheila A.: 5155
Duggan, Katherine: 1360, 7550
Dujela, Carren: 7470
Dumitrescu, Ramona Gianina: 2270
Dunbar, Annie Zean: 4195
Dunbar-Jacob, Jacqueline: 9010, 9540
Duncan, Carly A.: 2180
Duncan, Francesca E.: 3215
Duncan, Jacob: 7210
Dunkle, Ruth E.: 1170, 4325
Dunlap, Janette: 7180, 9000
Dunlap, Pamela: 1015, 4145
Dunleavy, Daniel: 1030
Dunnam, Charles W.: 9000
Duong, Mylinh: 4268, 7680
Duplàa, Emmanuel: 2390
DuPont-Reyes, Melissa: 5195
Duppen, Daan: 1065, 7280
Dupre, Matthew E.: 2265, 3180, 9000, 9020
Dupree, Galilea D.: 7980
Duque, Gustavo: 1060, 2190
Duran, Danielle: 9000
Durant, Raegan: 9000
Durga, Aditi: 2070
Dury, Sarah: 1385, 4330
Duthie, Edmund H.: 7310
Duynisveld, Amber: 4515
Dwibedi, Aditya Kumar: 9010
Dwyer, Johanna T.: 9020
Dy, Sydney L.: 1035, 4170
Dye, Maya: 1120
Dymchuk, Emily: 3335, 4025
Dys, Sarah: 1210, 3320, 5030, 9010
Dyslin-Kman, Deborah: 7700
Dzomeku, Veronica: 7790

E

Ea, Emerson: 9020
Earles, Julie L.: 7510
Earnshaw, Valerie: 2055
Easley, Mikki: 2245
Easton, Scott: 1130
Eaton, Andrew D.: 4340
Eaton, Jacqueline: 1150, 1250, 2075
Eaton, William W.: 7870
Ebner, Natalie C.: 9010, 9200
Eby, David W.: 9030
Echavarria, Diane: 1035
Eckstrom, Elizabeth: 2385, 7430
Edahiro, Ayako: 7280
Eddington, Blake: 9020
Edelman, Linda S.: 4132, 4406, 5190, 7000, 7500, 9000, 9020, 9030
Edelstein, Offer E.: 1075
Edland, Steven: 7100
Edmonds, Tanner: 9010
Edwards, Chelsea: 2085
Edwin, Trine Holt: 9020
Ee, Nicole A.: 9110
Efird-Green, Lea: 3320
Egan, Mary: 9000
Egashira, Leo: 9100
Eguchi, Hisashi: 9030
Ehlman, Katie: 9000, 9020
Ehmann, Madison: 4460
Ehrlich, Joshua: 2135, 2150, 2365, 9110
Eimicke, Joseph P.: 1145
Eisenberg, Carol: 9000
Eisenberg, Chase: 9000
Eisenstein, Amy R.: 4085
Eisikovits, Zvi: 4520
Ejaz, Farida K.: 2060, 2220, 4215, 7060, 7460
Ejem, Deborah: 4060
Ek, Stina: 5205
Ekerdt, David J.: 1185, 9030
Ekman, Monica: 5040
Ekoh, Prince: 9010
Ekwebene, Onyeka: 5055
Ekwonye, Angela: 7960
El Khoudary, Samar R.: 4120, 5155
El-Abbadi, Naglaa H.: 1290
Elayoubi, Joanne: 7960
Elbejjani, Martine: 9500
Elbich, Daniel B.: 5165, 7510, 9030
Eldadah, Basil: 3040
Eldreth, Dana: 9000
Eldridge, Corinne: 9020
Eldridge, Madeleine R.: 4165
Elfenbein, Pamela: 4125
El-Hulu, Sondos: 7100
Eliazar-Macke, Nathaniel: 2170
Elkjær, Emma: 3195
Ellen, Moriah: 1175, 4255
Ellington, Lee: 1150, 2075, 7470, 7540
Elliott, Amy: 7600
Elliott, Cerise L.: 1355
Elliott, Emily M.: 2105, 7550
Elliott, Lauren: 7830
Elliott, Sheryl: 9010
Ellis, Amy: 7620, 9000, 9020
Ellis, Dylan: 5180
Ellis, Jennifer: 9400, 9440
Ellis, Julie: 7610, 7640
Ellison, James M.: 7620
Ellison Keeler, Carlyn: 9020
Ellis-Smith, Clare: 4470
Elman, Miriam R.: 7360
Elo, Irma: 7650
Elran-Barak, Roni: 7070
Elrod, Cathy: 9010
Elswick, R.K.: 9330
Eltaybani, Sameh: 7160, 7720, 9520
Elumeze, Josephine: 4268, 7680
Emery-Tiburcio, Erin E.: 4540
Emmesjö, Lina: 7130
Emmons, Stacey: 9020
Emrani, Sheina: 7100
Engdahl, Bo: 9020
Engeland, Christopher G.: 3545, 4490, 9010, 9030
Engelman, Michal: 3005, 3105, 3450
Engler-Chiurazzi, Elizabeth: 9010
English, Tammy: 1105
Engstrom, Gabriella: 7670
Enguidanos, Susan: 7260
Ennis, Nicole: 1030
Enroth, Linda: 4395, 9000
Ensrud, Kristine: 4260, 7650
Eom, Minjeong: 7810
Eost-Telling, Charlotte: 4455
Eppich, Kaleb: 1355
Epps, Fayron: 1265, 2070, 2180, 3305, 3485, 7440, 7620, 9030
Epstein, Cynthia: 2090, 9320
Epstein-Lubow, Gary: 2030
Eramudugolla, Ranmalee: 3570
Erdol, Nurgun: 7620
Erickson, Mary Ann: 2245
Erickson, Melissa L.: 3475
Ericsson, Malin: 9020, 9030
Eriksson, Erik: 2340
Eriksson, Johan G.: 9010
Erisman, Morgan: 1205
Ermer, Ashley: 1185
Ermoshkina, Polina: 3360, 7890
Ernst, Jerome: 3565, 7810
Ernst, Joy S.: 4266, 7780
Ersek, Mary: 4005, 4470
Ertle, Emilee: 7090, 7420, 9010
Erving, Christy L.: 2275
Erz, Dorothee: 9020
Escobar, Romeo: 4240
Esiaka, Darlingtina: 1355, 2010, 2110, 4130, 5055, 7520, 7960, 9210
Espejo, Luis: 7870
Espeland, Mark A.: 2320, 4355, 5060, 7630
Espinoza, Sara E.: 4355, 5055
Espinoza, Sara: 7180
Espinoza, Sara E.: 7370, 7660, 7910
Esser, Karyn: 3475, 3500, 4335, 7370
Esslemont, Dawn: 7160
Estabrooks, Carole A.: 3335, 4408, 4515, 7720
Estapé, Jorge: 2260
Estapé, Tania: 2260
Estenson, Lilly: 3220, 7750
Estlein, Roi: 7070
Etnier, Jennifer L.: 9030
Ettema, Roelof GA.: 4125, 7350
Euiler, Elizabeth: 4005
Eun, Yong: 7330
Evangelista, Lorraine S.: 4040
Evans, Catherine: 4470
Evans, Donald: 3080, 9000
Evans, Elizabeth A.: 5050, 7520
Evans, Joni K.: 4355
Evans, Lauren: 4380
Evans, Luke: 9010
Evans, Michele: 9000
Evans, Michele K.: 4130, 7560
Evans, Tracy: 7850
Evans, William J.: 4260
Evardone, Vanessa Joyce M.: 7840

Everink, Irma: 7720
Extermann, Martine: 7410
Eyler, Gabriella: 9020
Ezeamama, Amara: 7930
Ezeokonkwo, Francesca C.: 3485

F

Fabbre, Vanessa: 1250, 4195
Fabius, Chanee D.: 2185, 3415, 5130, 5160
Factora, Ronan: 7130
Fadalla, Carol: 7830
Fadel, Meghan: 3055
Fagundes, Christopher P.: 9110
Fahey, Margaret C.: 2175
Faig, Karla: 7660, 7830, 9010
Fairbanks, Sheryl A.: 1025, 7470
Fairchild, Kaci: 9010
Fairman, Ciaran M.: 2260
Falchetta, Giacomo: 2105
Falciola, Justine: 3130
Falck, Ryan S.: 5100
Falcon Borbolla, Veronica: 9010
Falenchuk, Olesya: 9550
Falvey, Jason: 3550, 7340, 9000
Falzarano, Francesca: 1315, 2050, 2075, 3170, 3385, 4010, 7090, 7300, 7420
Fan, Erica K.: 3405, 9500
Fan, Jin: 4400
Fan, Rong: 4200
Fan, Sheng-Yu: 9030
Fan, Xing: 7660
Fan, Yi: 9540
Fan, Zhiyu: 5115, 7000, 7060, 7950
Fan, Zhongqi: 1290
Fang, Mei: 3255
Fang, Mei Lan: 4245
Fang, Qiyin: 9020
Fang, Ya: 9000, 9020, 9110, 9560, 9580
Fang, Yuan: 7640, 7830
Farah, Kimberly: 2080
Farina, Mateo P.: 2050
Farina, Mateo: 2325, 3465
Farley, Jason: 1375
Farmer, Heather R.: 1070, 3080, 4055
Farnham, Trish: 9530
Faro, Jamie M.: 9030
Farrell, Shannon M.: 3480, 9000
Farrell, Timothy: 9000
Farsijani, Samaneh: 3475, 4260, 5085
Fashaw-Walters, Shekinah: 1380, 9020
Fasola, Serena: 9030
Fassi, Janelle E.: 3385, 9000
Fast, Janet: 7980
Fasth, Lauren M.: 7300
Fastring, Danielle: 1070
Fattorini, Eliana: 7070, 7600
Faul, Anna: 4040, 4360
Faul, Jessica: 2320, 2325, 3225
Faulkner, Sandra: 4390
Fauth, Elizabeth B.: 7400, 7940, 7990, 9030
Fazal, Amaanali: 2020
Fazeli, Pariya L.: 4340
Fazio, Elena: 2150
Fazio, Sam: 3205, 7420, 7540, 9020, 9300
Feder, Shelli A.: 9030
Federwitz, Chad F.: 3485
Feinhals, Gretchen: 3305
Feinn, Rich: 9300
Feitosa, Mary F.: 4260, 5090, 9010, 9020
Felderhoff, Brandi J.: 7310
Feldman, Boris: 7820
Feldman, Corinne: 3220
Feldman, Sue S.: 3365, 4190
Feliachi, Zohra: 8900
Feliciano, Leilani: 4268, 7680
Felix, Laura L.: 5145
Felt, John M.: 5165
Feltmate, Patrick: 9010
Felts, Adam: 1175, 3090
Fendrich, Michael: 1390
Feng, Du: 4134, 7330
Feng, Hui: 7640, 9300, 9320
Feng, Liang: 9020
Feng, Yali: 7010
Feng, Zhe: 1390
Fennell, Charles: 4100
Fennell, Gillian: 1165, 7380, 7460
Fenstermacher, Erika A.: 7030, 7570
Fenton, Laura: 4275
Ferdows, Nasim: 9000, 9030
Ferguson, Erin L.: 2240
Ferguson, Giselle A.: 4045
Ferguson, Lee Ann S.: 9010
Ferguson, Melissa M.: 4370
Fermin-Martinez, Carlos A.: 3160, 4395
Fernández-Chirino, Luisa: 4395
Fernandes, Deanna C.: 3500, 7140
Fernandez Cajavilca, Moroni D.: 1150, 2075
Fernandez-Chirino, Luisa: 3160
Ferrari, Michel J.: 1390
Ferraro, Kenneth F.: 3230, 4535, 9010
Ferreira, Erika A.: 7340
Ferreira Leitao Azevedo, Renato: 1260, 9540
Ferreri, Stefanie: 7150
Ferri, Cleusa Pinheiro: 3160
Ferrucci, Luigi: 1295, 2155, 2195, 2240, 2285, 3510, 4035, 4265, 4490, 5085, 9020, 9300
Fetzko, Joanne E.: 2315
Fiala, Jacob A.: 2180, 5070
Fick, Donna: 4315, 4525, 9010, 9540
Fiehn, Oliver: 7110, 7880
Fielding, Roger: 1290
Fields, Beth: 2070, 2170, 3030, 5000, 7800, 9030
Fields, Maizonne: 7090
Fields, Noelle L.: 2390, 3305, 9010, 9030
Figeys, Mathieu: 9000, 9020
Figueiredo, Daniela: 7300
Filec, Sarah: 2355, 9030
Filewich, Andrew: 7700
Filiatrault, Johanne: 7620
Fillingim, Roger B.: 4134, 7330, 7630, 9010
Finch, Caleb: 9030
Finer, Elizabeth: 7340
Fingerman, Karen L.: 4145, 5045, 7070, 9000
Finkel, Deborah: 3470, 7300
Finkel, Deborah: 9020, 9030
Finkelstein, Ruth: 7230
Finley, Erin: 5055
Finley, Margaret A.: 4005
Finn, Jacob A.: 7420
Finn, Laura A.: 9030
Finnerty, Kaylyn: 7120
Finnie, Dawne: 9000, 9010
Finsel, Julia S.: 7290
Fiori, Katherine L.: 1235, 4230
Fiscella, Andrew J.: 7020
Fisch, Kathleen: 7100, 8900
Fischer, David: 8900
Fischer, Stacy: 7410
Fishbein, Ken: 2195
Fisher, Anna: 2295, 3175
Fisher, Kathryn: 5085, 5220, 7840
Fisher, Lauren: 4134, 7620
Fisk, Calley: 5050
Fiske, Amy: 7030, 7570
Fitch, John E.: 7680
Fitzgerald, Kyle: 9000
Fitzpatrick, Annette L.: 7830
Fix, Gemmae M.: 1375
Flanagan, Kelly: 9010
Flannagan, Carol A.: 7080
Flaster, Aaron: 9580
Fleming, Allison: 5165
Fletcher, Jason: 1130
Fletcher, Jason: 7230
Flett, Gordon L.: 3155
Fleysher, Roman: 1125
Flood, Sarah: 2050, 3060
Flores, Bertha E.: 7180
Flores, Elena: 1270
Flores, Emily: 7930
Flores-Cohaila, Javier: 9010, 9030
Floyd, Alexander: 5015
Floyd, Elizabeth: 9030
Flurry, Ethan: 7510
Fogg, Louis: 3445
Foley, Sophia: 9010, 9310
Follis, Shawna: 3210
Fonseca, Carolina: 4060
Fontanese, Matthew S.: 7080, 7900
Foradori, Megan A.: 4525
Ford, Alicia: 9000
Ford, Cassandra: 2125
Ford, J. Kevin: 9000
Ford, Tiffany N.: 2225
Forder, Michael: 3300
Forman, Daniel E.: 3475, 5210
Forster, Anne: 7720
Fortinsky, Richard H.: 2300, 4140, 7030, 7430
Fosnight, Susan: 4406, 7250, 7480, 7500
Foster, Liam: 4450
Foster, Thomas C.: 7630
Fountain, William: 9010
Fowe, Ibukun E.: 1040
Fowler, Mackenzie E.: 2260
Fowler, Michael: 4268, 7680
Fowler, Nicole R.: 4210, 5105, 5115, 9020
Fowler, Samantha A.: 9010
Fowler-Davis, Sally: 4245
Fox, Aimee: 4010
Foxworth, Judy: 4350
Fröberg, Andreas: 7170
Frager, Victoria A.: 1010
Frakes, Nathan: 9000
Framil, C Victoria: 4415
Francis, Erica: 5145
Francis, Sarah L.: 7590
Frangiosa, Theresa: 9000, 9010
Frank, Colleen: 1090
Frank, Jennifer S.: 4340
Frank, Mabel: 1065
Franke, Emily: 9010, 9540

Fransquet, Peter D.: 7370
Fransquet, Peter D.: 7370
Franz, Berkeley: 7780
Franzosa, Emily: 1020, 2145, 2235, 3415, 4165, 9010
Frase, Robert T.: 1050, 7920
Fraser, Sarah: 1200
Frazier, Charlotte: 2080
Frazier, Ian: 9010
Frazier, Jefferson: 9110
Frazier, Robbin H.: 1025, 2180, 7380
Frechman, Erica: 3395, 9030
Freddolino, Paul Peter: 2085, 4020
Frederick, Miranda: 3445
Fredericksen, Jessica: 7760
Fredriksen-Goldsen, Karen: 2310, 4000, 4195, 4390, 9010, 9440
Free, Julia: 7730
Freedman, Jonathan D.: 2225
Freedman, Vicki A.: 1340, 2150, 3390
Freeman, Shannon: 2340, 5105, 7160, 7300, 9020, 9030
Freeman, Valerie: 9030
Freemdan, Vicki: 1340
Frei, Anja: 3550
Freitas, Fabio Cavalcanti: 9020
French, Eric: 2255
French, Molly: 3045
Frenier, Christopher: 3010, 4355
Frey, Katelyn T.: 7520
Freytes, I. Magaly: 2170
Friberg-Felsted, Katarina: 7480
Fridriksson, Julius: 8900
Fried, Linda P.: 1110
Fried, Terri R.: 3360, 4010
Friedman, Ari B.: 3530, 7860, 8900, 9030
Friedman, Daniela B.: 2260
Friedman, Esther M.: 1340, 2100, 7530
Friedmann, Erika: 4040
Friel, Ciaran: 4268, 7680
Friis, Marlene: 7550
Fritschi, Cynthia: 4025
Fritz, Joëlle V.: 9020
Froberg, Ruthann: 2030
Fromm, Dylan: 9000
Frost, Alanna: 9030
Frost, Melissa: 7590
Fruhauf, Christine A.: 3060, 3385, 4010, 4400
Frungieri, Monica: 9020
Fry, Liam: 4290
Fu, Carolina: 9010
Fu, Cheng: 3115
Fu, Rong: 7230
Fu, Xiaoyu: 4085
Fu, Ying-Kai: 4134, 7620
Fuentealba, Matias: 1380
Fugate Woods, Nancy A.: 5155
Fugate-Whitlock, Elizabeth: 2245, 3350
Fujihira, Kyoko: 7550, 7760
Fujita, Koji: 7550, 7580, 7760
Fujiwara, Natsumi: 4505, 9000
Fujiwara, Yoshinori: 7550, 7580, 7760
Fukuda, David H.: 7150, 9010, 9020
Fukui, Chie: 7160
Fukui, Chie: 9520
Fuller, Heather: 1235, 3380, 5035, 7710, 7810, 9030
Fuller, Kristi: 7690
Fuller, Patrice: 4100
Fuller-Rowell, Thomas E.: 9000
Fuller-Thomson, Elysia G.: 1350
Fuller-Thomson, Esme R.: 1350
Fulmer, Terry: 2060, 4132, 7500
Fung, Helene H.: 3100, 3400, 4480, 7050, 7060, 9020, 9030
Fung, Kathy: 5230, 7160
Fung, Nicole Long Ki: 3100
Fung, Victor: 2390
Funghetto, Silvana Schwerz: 7130, 7150, 7340
Funk, Cory: 5180
Furber, John D.: 9030
Furht, Borko: 5225
Furman, David: 1380
Furmanek, Stephen: 9000
Fusaris, Ethan: 3565, 7810

G

G Monteiro, Joao Filipe: 9010
Gaba, Anu: 7600
Gaballa, Fairouz: 7420
Gabbay, Mark: 9000, 9570
Gabois, Emily: 4290, 7620, 9000, 9030
Gabrielsson-Järhult, Felicia: 7180
Gad, Dustin: 7420
Gadd, Danni A.: 4134, 7620
Gadkari, Gauri: 1210, 9030
Gaeta, Raphael: 2220
Gagen, Travis M.: 7780
Gagnon, Camille: 9520
Gagnon, David R.: 9010
Gagnon, Sylvain: 7060
Gahche, Jaime J.: 9020
Gahn, Katie: 7570
Gai, Junjie: 7760
Gaillard, Trudy: 7810, 9010
Gajraj, Asha: 9010
Galasko, Douglas: 8900
Gale, Kathy S.: 7910
Galea, Sandro: 2105, 7550
Galik, Elizabeth: 2305, 3330, 3340, 7170, 9020
Galladay, Katelyn A.: 3075
Gallagher, Amelia M.: 3140
Gallagher, Elizabeth A.: 3130
Gallagher Watkin, Laura: 5135
Gallagther-Thompson, Dolores: 7030
Gallegos, Adrianna C.: 7410
Gallibois, Molly: 7830
Gallo, Joseph: 3415, 4460
Galucia, Natalie: 9000
Galvin, Angéline: 5090
Galvin, James: 3490
Gamaldo, Alyssa A.: 2275, 3245, 4055, 7560, 7970
Gamaldo, Alyssa A.: 9020
Gamble, Jean-Michael: 4280
Gamble, Laura: 4070
Gammonley, Denise: 1100
Gan, Daniel R Y.: 5030
Ganann, Rebecca: 5085, 5220, 7840
Ganbat, Uyanga: 7820
Ganci, Aaron: 7080
Gandy, Maribeth: 7990
Gandy, Tyler: 7960
Gange, Stephen J.: 3390
Gangnon, Ronald: 2230
Ganguli, Mary: 5080
Gangupantula, Pratima: 9010
Gans, Kim: 4030
Gao, Jie: 7680
Gao, Shiying: 7120
Gao, Sibo: 7070
Gao, Xianqi: 7660
Gao, Xiaojun: 7420
Gao, Yubo: 2180
Gaona, Carlos A.: 4100
Garabedian, Pamela: 9000
Garber, Johnathon D.: 7150
Garces, Edwin: 9030
Garces Torres, Ana Sophia: 9010
Garcia, Catherine: 1000, 3050
Garcia, Emmanuel: 2020
Garcia, Joshua M.: 1165, 3050
Garcia, Linda: 3260
Garcia, Mary Ellen: 8900
Garcia, Odalis G.: 7550
Garcia, Patricia: 2180, 5070
Garcia, Sandra: 9010, 9030, 9420
Garcia, Y. Evie: 7400
Garcia Morales, Emmanuel E.: 9010
Garcia Rogers, Juliet: 1325
Garcia Walker, Veronica: 2290
Garcia-Andres, Adelaido: 4240
Garcia-Aymerich, Judith: 3550
Gareri, Michele: 7250
Garg, Parveen K.: 3405
Garland, Paula: 7390
Garner, Jennifer: 2080
Garrett, Timothy: 9030
Garrison, Betsy: 7070
Garrison, Brianna: 9010, 9030
Garrison, Jennifer: 3215
Gary-Webb, Tiffany L.: 3405
Gasek, Nathan S.: 9030
Gassoumis, Zach: 2030, 2220
Gaster, Barak: 7830
Gately, Megan: 2085, 7850, 9020
Gates, Jessica: 9420
Gatz, Margaret: 2320, 9030
Gaugler, Joseph E.: 1050, 1150, 1265, 2130, 2190, 3020, 3235, 3240, 3300, 3305, 3460, 3570, 4170, 4290, 4415, 7420, 9000, 9010
Gauss, C. Heath: 7160
Gaussoin, Sarah A.: 7830
Gautam, Ramraj: 1080, 5200
Gaveras, Eleni: 4195
Gay, Emma L.: 3475, 5090
Gay, Jane: 7440
Gazarov, Emely: 1030
Gaziano, J. Michael: 4310
GB, Mia: 7870
Ge, Ning: 7350, 7830
Ge, Shaoqing: 7910
Ge, Shaoqing: 9010
Ge, Song: 3490
Gebara, Marie Anne: 5175

Geddes, Maiya: 7100
Gee, Nancy R.: 4040
Gefen, Tamar: 7700
Geiger, Olivia: 4025
Geisinger, Maria L.: 1075
Geisser, Sophia R.: 2110, 7540
Gelburd, Robin: 4525
Geldhof, John: 7580
Geldmacher, David: 9020
Gélinas, Isabelle: 7690
Gell, Nancy: 7990
Gellert, Paul: 9400
Gelmon, Sherril: 7770
Gendernalik, Jesssica: 7840
Gendron, Tracey: 3025, 3285, 3425
Genelyte, Indre: 4450
Geng, Jing: 4390
Genge, Courtney J.: 9020
George, Claudene: 3480
George, Joanna: 9000
George, Maureen: 9100
George, Mollie J.: 7550
George, Steven: 7410
Georgescu, Michael: 9000
Geraets, Anouk FJ.: 1225
Gerald, Melissa S.: 3525
Gergis, Mary: 4406, 7500
Germine, Laura Thi: 5165
Gerstorf, Denis: 1060, 3275, 4225, 4235, 7550, 9000, 9010
Gess, Peter: 7550
Getz, Marjorie A.: 7480
Ghare, Smita: 9000
Ghazali, Nadia: 1115
Ghazi-Saidi, Ladan: 2080
Ghezzi, Elisa: 1045
Ghiasi, Akbar: 2350
Ghimire, Saruna: 3380, 5220
Ghimire, Saurav: 7880
Ghisletta, Paolo: 4185
Ghose, Urmimala: 9010
Ghosh, Bhaswati: 4475, 9030
Ghosh, Nayanika G.: 2385
Ghosh, Ritam: 9030
Ghosh-Dastidar, Bonnie: 3405
Giambrone, Alessandro: 3160
Giandrea, Michael D.: 3370
Giannotto, Emily L.: 7510, 9020
Giannouli, Eleftheria: 3115
Gibbons, Ian: 9010, 9020
Gibbons, Sean M.: 9020
Gibson, Allison: 3305, 5020, 7060
Gibson, Carolyn: 2280
Gibson, Ethlyn: 9010
Gibson, Regina V.: 4130
Gichu, Muthoni: 9110
Giebel, Clarissa: 9000, 9570
Gieniusz, Marzena: 4030
Gietzen, Luke J.: 7010
Gifford, Allen L.: 1375
Gifford, David: 9000
Giffuni, Jamie: 7840
Gigi, Amateau: 3425
Giles, Sadie: 4390
Gilfix, Talia: 9030
Gill, Jessica: 4310
Gill, Navjot: 7780
Gill, Thomas: 1165, 3230, 3450
Gillam, Juliet: 4470
Gilleard, Chris J.: 4245, 7060
Gillespie, Dylan: 7420
Gilliam, Meredith: 7300
Gilligan, Megan: 1050, 2075, 4275, 7920
Gills, Joshua L.: 2015, 7810
Gillsjö (Gillsjo), Catharina: 7000, 7130, 7310
Gilmore-Bykovskyi, Andrea: 7360
Gilson, Aaron M.: 9030
Gimm, Gilbert: 1335, 9030
Gin, June L.: 2295
Gionet, Sherry: 7660
Giordani, Bruno: 7510, 9020
Giordano, Stephanie: 3010, 7810
Giovannetti, Erin: 1035, 4170
Girdwood, Tyra: 4380
Girling, Laura: 7540, 9320, 9500
Giroux, Dominique: 9000
Gitlin, Laura N.: 1035, 2130, 3240, 4005, 5130, 7550, 9000, 9400
Giunta, Nancy: 5150
Gladyshev, Vadim N.: 4065, 7370
Glashutter, Margit: 7120
Gleason, Carey E.: 5000, 7100
Gleason, Kelly T.: 2185
Gleason, Shayna: 4150, 5110
Gleissner, Laura: 2390
Glenn, Jordan M.: 2015, 7810
Glesby, Marshall J.: 5115
Glicksman, Allen: 3530, 4285, 9030
Gloger, Elana M.: 9020
Glorioso, Danielle K.: 7610
Glowinski, Sarah: 1190
Glusman, Gustavo: 7110
Glymour, M Maria: 2240, 2300
Glynn, Nancy W.: 1295, 3390, 3475, 3500, 5090, 5210, 7650, 9030
Gmelin, Theresa: 1295
Gobaud, Ariana: 2225
Goderis, Geert: 7120
Godina, Sara L.: 3405
Gofine, Miriam: 7640
Goh, Dion Hoe Lian: 3295
Goldberg, Allon: 7150
Goldberg, Anne J.: 7550
Goldberg, Jaime: 3170, 7050
Goldberg, Renada M.: 7230
Golden, Adam: 7680, 7870
Golden, Blair: 1190
Golden, Dale M.: 9010, 9020
Golden, Justine: 4260
Goldgof, Dmitry: 7100
Goldman, Alyssa: 1350, 5050
Goldman, Dana P.: 4355, 4385
Goldman, Noreen: 3065
Goldman, Stuart: 7800
Goldstein, Nathan E.: 4266, 7190
Goldsworthy, Mary: 9020
Goler, Timothy D.: 1070
Golonka, Ona: 4100
Golstein, Nathan: 1375
Gomes, Ana: 2065
Gomes da Rocha, Carla: 3130
Gomes Goncalves, Natalia: 3160, 7320
Gomez, Carlos A.: 9420
Gomez, Jennifer: 3090
Gondo, Yasuyuki: 3555, 9000
Gonyea, Judith: 7670
Gonzales, Ernest: 4085
Gonzales, Mitzi: 4100, 5055, 7660
Gonzalez, Alexander: 5120
Gonzalez, John: 4240
Gonzalez, Richard: 3105
Gonzalez Castillo, Jorge: 9030
Gonzalez-Freire, Marta: 2195
Good, Mary: 9020
Goodale, Sarah E.: 1215
Goode, Adam: 7410
Goodman, Claire: 2375
Goodman, Octavia: 8900
Goodpaster, Bret H.: 3475, 5210
Goodwin, Rebecca: 4365
Gooldin, Sigal: 9030
Gorbunova, Galina A.: 5015, 5110
Gorbunova, Vera: 4065
Gordon, Barbara: 4040, 5190
Gordon, Julie A.: 9020
Gordon, Kate: 3235, 9030
Gordon, Michelle: 3505
Gordon-Wexler, Mikayla: 3415
Gore, Janelle E.: 2070, 2180, 3485
Gore, John: 9010
Gorla, Vandita: 9020
Gorman, Shannon: 7180
Gorospe, Myriam: 5060, 7880
Gorzelle, Gregg: 9400, 9550
Gosselin, Patrick: 7030
Gothard, Sarah: 4100, 4495, 7440
Gottesman, Eve M.: 7000
Gottesman, Rebecca F.: 3510
Gouaux, Ben: 4340
Gouelle, Arnaud: 9330
Gould, Christine E.: 3250, 4095
Goulet, Joseph L.: 4300
Gouse, Hetta: 4340
Gouskova, Natalia: 2155
Gouveia E Silva, Erika Christina: 9010, 9020
Gowen, Emma: 1130
Gozalo, Pedro: 7160
Grabowski, David: 1115
Gracner, Tadeja: 4385
Grady, Alexandra: 7450
Grady, James: 4140
Graessel, Elmar: 2370
Graf, Allyson S.: 2080
Graf, Peter: 4480
Graf, Sylvie: 3100
Graff, Maud: 3340
Graham, Carrie L.: 5025, 7250
Graham, David: 5075, 7910
Graham, Eileen K.: 1255, 3520, 4300
Graham, Kirsten L.: 1365
Graham, Laura A.: 5230, 7160
Graham, Liz: 7720
Graham, Lyndsey N.: 3195
Graham, Robert J.: 3045
Graham-Engeland, Jennifer E.: 3500, 3545, 4490, 9010, 9030

Grant, Jeremy G.: 9210
Grant, Shakira J.: 2355
Graupner, Jeffrey P.: 9010
Gravenstein, Stefan: 7620, 9010
Graves, Kaitlyn: 7420
Gravitt, Patti E.: 4080
Gray, Jeff: 4415
Gray, Laura: 2375
Gray, Michelle: 2015, 7810
Gray, Nicholas: 1315
Gray, Vicki: 9000
Greaney, Mary L.: 4025
Greco, Frank: 9020
Greeley, Brian: 7160
Green, Cara L.: 7880
Green, Ginny: 8900
Green, Kerry M.: 4408, 7230
Green, Makenna R.: 4406, 7480
Greenberg, Jonathan: 4460
Greenberg, Phyllis A.: 2095, 4406, 7000
Greenberg, Sherry A.: 4005
Greenblatt Kimron, Lee: 3275
Greendale, Gail A.: 4120
Greenfield, Emily A.: 1330, 2315, 3045, 4150, 4195
Greenwood-Hickman, Mikael Anne: 1010, 3490
Greer, Valerie Chang: 4132, 7500
Gregg, Jeffrey J.: 3250, 4095
Gregg, Jordan: 5060
Gregory, Sarah: 7630
Gregson, Geoffrey: 9000
Greiner, Chieko: 9010
Grelle, Kaitlin: 7420
Grenier, Amanda: 4450, 7190
Grenier, Sébastien: 7030
Grewal, Ashmita J.: 5145
Grewal, Karl: 1200
Griffin, Joan: 9000, 9010
Griffin, Meridith: 5100
Griffin, Rebekah: 7080
Griffith, Christopher X.: 4268, 7680
Griffith, Lauren E.: 4268, 4280, 5070, 5220, 7170, 7660, 7680
Griffiths, Alys: 7720
Griffiths, Jane: 4110
Griffiths, Patricia: 7990
Grill, Joshua: 4420
Grimm, Kevin J.: 7610
Grimmett, Hailey: 2390
Grippa, Rosa: 5105
Griswold, Allison: 9030
Griswold, Michael: 5085, 7340, 9200
Grittner, Alison: 1205
Grocke, Michelle: 5075
Grodstein, Francine: 2120
Groen, Wim: 2370
Gronlund, Carina: 9010
Gross, Alden: 2150, 2240, 2365, 3390, 3510, 5175, 9110
Gross, Danae C.: 4268, 7680
Grossberg, George: 9020
Groulx, Mark: 2340
Groves, Angela: 7800
Groves, Erik: 9020
Growney, Claire M.: 1105, 4480, 4495, 8900
Gruber-Baldini, Ann L.: 3550, 4260
Grudzien, Adrienne: 9000
Gruenewald, Tara L.: 2320
Grullon, Jason Rafael: 9020
Gruneir, Andrea: 4280
Gruss, Valerie: 4025
Gu, Danan: 7080, 7590
Gu, Xiangli: 7730
Gu, Xiuzhu: 7290
Guan, Hua: 9020
Guan, Weihua: 5100
Guastaferro, Kate: 9010
Guerbaai, Raphaëlle-Ashley: 7160
Guerrier, Tami: 4350
Guess, Aundaray: 1250
Guggenheimer, Joshua: 8900
Gugliucci, Marilyn R.: 2095, 2215
Guida, Vincent: 7500
Guillemot, Jonathan: 4266, 4406, 7000, 7190, 7250, 7290
Guimaraes, Rafael: 7610
Guimond, Josée: 1280
Guinn, Victoria LA.: 3050
Guitar, Nicole A.: 7160
Gum, Amber M.: 3560, 4010, 9020
Gunderson, Rachel: 9030
Guo, Chao: 9000, 9010, 9030, 9210
Guo, Crystal: 7850
Guo, Man: 3210, 4050
Guo, Nicholas: 8900
Guo, Yingqqi: 3115
Gupta, Shivani: 7210
Guralnik, Jack: 4260
Gurlu, Merve: 4005, 4040, 9020
Guruge, Sepali: 9010
Gurven, Michael D.: 9030
Gur-Yaish, Nurit: 7050
Guseva, Elena: 7080
Gusmano, Michael K.: 3165
Gustafson, Deborah: 3565
Gutierrez, Angela: 1115, 7780
Gutierrez, Mariela: 9030
Gutierrez, Sirena: 7600
Gutierrez-Baca, Wendy: 9010, 9030
Gutman, Roee: 4290
Guyonnet, Sophie: 2320
Guzman, Rebeca: 7680

**H**

Haaland, Perry: 7360
Haapanen, Markus: 9010
Haardörfer, Regine: 3565
Haas, Meghan: 7360
Haas, Steven A.: 2360
Haase, Claudia M.: 1105
Haase, Joan: 9400
Håberg, Asta K.: 9020
Haché, Josée: 7830
Hachen, Naomi: 3480, 9000
Hackel, Emily: 9020
Hacker, Eileen: 9440
Hackett, Sara E.: 2030
Hacking, Coen: 1260, 7800
Haddad, Jeffrey: 7060
Haddad, Yara L.: 9010, 9100
Hadlandsmyth, Katherine E.: 2180
Hafeez, Mohib: 9020
Hafner, Camryn: 2095
Hagedorn, Aaron: 9000
Haggar, Katherine: 1055
Haggler, Jayden Austin: 7100
Hahn, Sarah J.: 2075
Hahne, Jessica: 9020
Haigh, Sylvia V.: 4290
Hailer-O'Keefe, Laurie: 3285
Hairi, Noran Naqiah: 4266, 7190
Hajcak, Greg: 7030
Hajdar, Melisa: 1235, 7710
Hakanson, Megan: 5025
Hakim, Talib: 7900
Häkkinen, Emilia: 4330
Hakun, Jonathan G.: 5165, 7510, 9030
Halasz, Ildiko: 9010
Halaszynski, Jaime J.: 2030
Haldar, Srabani: 9030
Hale, Timothy M.: 1260
Hale, Willie: 7420
Haley, William E.: 2245, 3345, 7420, 7510, 7960
Hall, Alex: 4455
Hall, Anna: 7610
Hall, Charles: 9020
Hall, Daniel: 2180
Hall, Katherine: 7840
Hall, Kelli S.: 1110
Hall, Kendall: 7810
Hall, LaToya N.: 3090
Hall, Lawrence: 7100
Hall, Martica: 5155
Hallen, Sarah: 7150
Haller, Landon J.: 7180
Hallgren, Jenny: 7000, 7130, 7310
Halonen, Pauliina: 9000
Halpin, Sean N.: 2260
Halter, Colt: 7510
Halvorsen, Cal J.: 3095, 3290, 4210, 4255
Hamada, Fumiko: 3135
Hamama Raz, Yaira: 3275
Hamel, Alexander: 9030
Hamer, Mark: 2015
Hamers, Jan PH.: 1260, 7720, 9010
Hamilton, Alice: 7950
Hamilton, Jill: 3485
Hamler, Tyrone C.: 2050, 3005, 7410
Hamm, Jeremy: 1360, 7550, 7990, 9000
Hammel, Edward W.: 9000
Hammel, Iriana: 9030
Hammel, Joy: 7360
Hammers, Dustin: 4210, 5105, 7100
Hammond, Allison: 7390
Hammouri, Ammar: 7610, 7640
Hampton, Kischa: 1265
Hamrick, Mark: 9010, 9030
Han, Chengming: 4305
Han, Duke: 3090, 4275
Han, Gaeun: 7510, 7940
Han, Gang: 7430, 7760
Han, Gyounghae: 3130
Han, Ling: 2360, 9030
Han, Sae Hwang: 4255, 7020
Hanaoka, Hideaki: 7780
Hancock, Cynthia R.: 3350
Hancock, David W.: 2060
Hancock, Laura M.: 2240
Hand, Michelle D.: 3310, 7230
Handberg, Eileen: 9580
Handelsman-Nielsen, Mika: 7360
Hanes, Douglas W.: 5110, 7590
Hanlon, Alexandra L.: 4545, 9020

Hanna, Kerry: 9000
Hanna, Sophie: 7510
Hanna, Valecia D.: 1035, 4170
Hannah, Camille J.: 5215
Hannan, Marian T.: 2385
Hanratty, Barbara: 4455, 4540, 7160, 9010
Hansen, Abigail: 3570
Hansen, Jared: 9420
Hansmann, Kellia J.: 2230
Hanson, Laura C.: 4005, 7300
Hanssen, Denise: 9000
Hansson, Asa: 7380
Happ, Mary Beth: 3345
Harada, Ann SM.: 4355
Harada, Ko: 7860
Hardern, Sonia: 3270
Harding, Alyson B.: 1015
Hardison, Holland: 7150
Harlock, Sarah: 7830
Harlow, Sioban D.: 5155
Harman, Sherman M.: 7100
Harnett, Tove: 5110, 7190
Harnish, Kathryn: 4215
Harper, Colin: 9010
Harrell, Rodney: 2315
Harries, Priscilla: 7690
Harrington, Charlene: 9030
Harrington, Erin: 7510, 9010
Harrington, Erin E.: 9010
Harrington, Karra D.: 5165, 7510
Harris, Frances: 7990
Harris, Jolie: 4250
Harris, Maurita T.: 7680
Harris, Rebekah: 9010
Harris, RianSimone: 4400
Harris, Tina Marie: 7360
Harris-Love, Michael O.: 9020
Harrison, Sean: 9030, 9540
Harrison, Tracie Culp: 2290, 4155, 7370
Harrison, Zinnia: 4195
Harrison Bush, Aryn L.: 7330
Harris-Speight, Jasmine: 7590
Hart, Abigail: 7550
Hart, Foxx: 9020
Hart, Sara: 7470, 7540
Hartford, Emily G.: 4510
Hartmann, Christine W.: 3365, 3415, 4290, 7540
Hartnell, Lisa M.: 2195
Hartzog, Meggan: 7540
Harvath, Terri A.: 2215
Harvey, Kaitlyn: 3310
Harvey, Marie: 4105
Harvey, Philip: 3515
Harvey, S. Marie: 1240, 4105
Hasan, Mehedi: 9000
Hasche, Leslie: 4195, 5200
Hasmanova Marhankova, Jaroslava: 9030
Hass, Zachary: 4215, 7790
Hassenstab, Jason: 3245
Hassett, Afton: 7680
Hasson, Seana: 1065
Hastings, Susan: 7150
Hathaway, Wendy: 1005
Hathaway, Zachary: 3395
Hattakitjamroen, Varitnan: 9580
Hattori, Robin: 1255
Haunch, Kirsty Jayne: 2375, 7720
Hausman, Hannah: 8900
Haverhals, Leah M.: 1160, 2295, 4408, 7160, 7720, 9310
Havers, Fiona P.: 4130
Haviland, Kelly: 7410
Hawes, Courtney: 9000, 9030
Hawes, Frances M.: 3295, 9030
Hawkley, Louise: 4205
Hawley, Chelsea: 2085, 7770
Hawley, Sarah: 1085
Hayajneh, Audai: 7330
Hayashida, Cullen T.: 7280
Hayden, Kathleen M.: 4355
Hayes, Shannon: 5100
Hayes, Timothy: 9000
Haynes, Richard: 7060, 7190
Hayslip, Bert: 3060, 4400, 7530
Hazany, Farnaz: 4420
Hazelett, Susan: 7250
He, Daqing: 5235
He, Jiamin: 7140, 7330
He, Lingxiao: 2320, 9000, 9020, 9580
He, Mengying: 4190
He, Qianyu: 2255
He, Ranran: 1050
He, Yuelin: 3225
Head, Elizabeth: 1215
Hearn, Gesine: 7610
Heath, Diana: 1305
Heaton Curtis, Elizabeth: 1310
Hebert, James R.: 2260
Heckman, Rosalind: 7020
Hedeker, Donald: 9030
Heeb, Rachel: 7780
Heemskerk, Amber: 9010, 9200
Heffernan, Kevin S.: 7970
Heffner, Kathi L.: 1335, 4460, 5185
Heideman, Kristin: 9020
Heid-Grubman, Jeanne: 9000
Heijmans, Bas: 2285
Heiland, Frank: 3080
Heilbrunn, Emily S.: 5145
Heimler, Stephanie R.: 7370
Hein, Maria: 2160, 3335, 5015, 7300
Heinrich, Allison: 4350
Heinz, Melinda: 2390, 7560
Heisel, Marnin J.: 3155
Heisz, Jennifer: 7180, 7340
Helbostad, Jorunn L.: 3550
Helene Choquet, Tom Hoffmann: 2240
Helm, Noah: 9330
Helmly, Victoria: 4115
Hemmings, Nina: 2255
Hemmy, Laura S.: 3460
Hemphill, John: 7830
Henderson, Christine: 7160
Henderson-Betkus, Mary: 9030
Hendriks, Ard: 7800
Hendry, Phyllis: 9010
Henke, Maria: 4435
Henkens, Kène: 4130, 9030
Henne, Mike: 3355
Henneberry, Kathleen: 4035
Hennessy, Catherine: 7190
Henry, Maureen: 1020
Hensley, Robert B.: 7460
Heo, Seongkum: 7850
Hepburn, Kenneth: 1010, 2070, 2130, 2300, 3235, 3435, 3535, 7420, 7440
Hepple, Russell T.: 3475, 5210
Herbstman, Julie B.: 1110
Herd, Pamela: 1000
Herman, Allison: 5060
Hermelbracht, James: 3350
Hermens, Lotte: 3340
Hernandez, Adriana: 4150, 9000
Hernandez, Alyssa: 3525
Hernandez, Bianca: 4132, 7480
Hernandez, Christopher D.: 4132, 7500, 9020
Hernandez, Fernanado: 7100
Hernandez, Guisela: 9010
Hernandez, Janice F.: 7980
Hernandez, Mario A.: 9010
Hernandez, Veronica: 9030
Hernandez Chilatra, Jessica A.: 5140
Hernandez Lepe, Fernando: 1185, 7950
Hernandez-Bigos, Kizzy: 4010
Hernandez-Lujan, Deisy: 9310
Hernandez-Tejada, Melba A.: 5140, 7010
Herr, Keela: 4134, 7620
Herrell, Cheyenne T.: 9010, 9020
Herrick, Tamara: 7150
Herrmann, Francois R.: 3130
Herrmann, Nathan: 7080
Hersey, Catherine: 4195
Hertel, Mollie: 5220
Hertzog, Christopher K.: 7510
Herzog, Monica Anderson: 4415
Heselton, Hyeon Jung: 7020
Heslin, Samita: 9020
Hess, Thomas M.: 3100
Hetherington-Rauth, Megan: 4260
Hettiarachchi, Pasan: 7170
Heun-Johnson, Hanke: 4385
Hewston, Patricia: 5100
Heyn, Patricia C.: 9010
Hiatt, Shirin O.: 3535
Hickey, Amber: 7400
Hickman, Susan: 9000, 9400
Hicks, Gregory: 7410
Hicks Patrick, Julie: 1365, 3165, 7450, 7760, 7910, 8900
Hida, Mitsumasa: 9030
Higa, Moritake: 2380
Higdon, Kristin: 7370
Higgins, Melinda: 2070, 2300, 7440
Higgins Tejera, César: 2225
Higgins-Chen, Albert: 9010
Higgs, Paul: 4245, 7060
Hilgeman, Michelle M.: 3365, 4290
Hill, Andrea M.: 3285
Hill, Caesar: 9000
Hill, Jordan R.: 7080
Hill, Kaliya: 4350
Hill, Nikki L.: 4425, 7830
Hill, Patrick L.: 3135, 3245, 3520, 7060, 9020, 9570
Hill, William D.: 9020, 9200
Hill-Ashford, Yolanda: 7680
Hill-Jarrett, Tanisha G.: 2225

Hillman, Charles: 7100
Hillstedt, Ebba: 9030
Hilsabeck, Robin C.: 5235
Himes, Dani: 2105, 3220
Hinrichs, Timo: 3115
Hirsch, Jana A.: 1015
Hirsch, Jen: 1280, 2330
Hirsch, Mark: 4350
Hirschman, Karen: 4134, 4545, 7620, 9010, 9020, 9030
Hirst, Sandra: 1145, 9030
Hirvela, Kari: 1190
Hladek, Melissa: 5175, 5230
Ho, Alvin: 3280, 9010
Ho, Hung Chak: 3115
Ho, Rainbow Tin Hung: 7610
Ho, Ringo Moon-Ho: 3085, 3295, 3470
Ho, Tai-Hung: 9030
Ho, Tuong-Vi: 1165
Hoàng, Isabella: 9000
Hoang, Van: 9530
Hobbs, Matthew: 1015
Hobday, John V.: 3235
Hoben, Matthias: 3335, 4025, 4280
Hochberg, Marc: 7330
Hockett, Samuel: 7570
Hodes, Richard: 3040, 3185
Hodge, David R.: 9030, 9300
Hodge, Oliver: 2260
Hodges, James: 4420
Hodgson, Nancy: 3485, 4005, 4134, 7330, 8900, 9030, 9540
Hoeffer, Charles: 9010
Hoffins, Emily L.: 9030
Hoffman, Geoffrey: 1340
Hoffman, Jeanne: 7280
Hoffman, Jessica A.: 3350
Hoffman, Rashelle: 3535
Hoffman, Yaakov: 3275
Hoffmann, Coles: 4035
Hogan, David B.: 4280, 7660
Hogans, Beth B.: 7800
Högnäs, Robin S.: 3265
Hogue, Angela: 4525
Hogue, Mary H.: 9010
Hohaus, Chiara: 7070
Holden, Richard J.: 7080
Holditch, Sarah C.: 7390
Holland, Alexandra: 7760, 9310
Holland, Peter: 7870
Hollandsworth Reed, Jenn: 7770
Holley, Kynleigh: 4350
Holley, Lyn M.: 3325
Hollingshaus, Michael: 4365
Hollingsworth, Holly: 7780
Hollins, Nancy: 7950
Hollis-Sawyer, Lisa: 1080, 3250
Hollister, Brooke: 9020
Holllingsworth, Alexis: 7900
Hollman, Nicholas: 9030
Holmes, Ashleigh: 7330, 9020
Holmes, Chad E.: 2295
Holmes, Rachel: 2015, 7370, 7800
Holmes, Sarah D.: 2210, 5020, 9020
Holt, Heidi: 3055
Holt, Janet: 2125
Holtgrave, Peter: 3055
Holton, John: 9000
Holtzer, Roee: 2155
Homan, Kristin: 7050
Homan, Melanie: 8900
Hon, Michelle: 9030
Honda, Ayumi: 7940
Honda, Sumihisa: 7940
Honfo, Sewanou Hermann: 1110
Hong, Andy: 7250
Hong, Dahye: 9010
Hong, Seunghye: 4025
Hong, Y. Alicia: 7750
Hong, Yinglu: 1260
Honig, Lawrence S.: 5090
Hood, Leroy: 5180
Hood, Michelle: 5155
Hoogendijk, Emiel O.: 7660, 9010
Hoogland, Aasha I.: 7410
Hook, Mary L.: 1190
Hooker, Julia: 4460
Hooker, Steven P.: 3390
Hooten, Madeline: 7100
Hoover, Andrew: 9010
Hooyman, Andrew: 9030
Hooyman, Nancy R.: 1280, 4135
Hopp, Faith P.: 1005, 4266, 7780
Hopp, Sarah: 1325
Hopper, Ingrid: 7300
Hopper, Shawna: 7060
Hoppmann, Christiane: 3275, 4235, 4480, 7550, 9000
Hoque, Masudul: 7620
Horgas, Ann L.: 7670
Horhota, Michelle: 1200
Horii, Mariko: 8900
Horn, Andrea B.: 4225
Horner, Sharon: 7980
Horning, Melanie A.: 7850
Horowitz, Chloe S.: 5080
Horstman, Molly J.: 7400, 7850
Hort, Jakub: 7420
Horta, Marilyn: 9010
Horwitz, Shelley: 5030, 7120
Hoselton, Jill H.: 1205
Hosking, Diane: 3570
Hossain, Mohammad Sajjad: 5020, 7060
Hossain, Sharmin: 4265
Hossain, Sheikh R.: 9000
Hosseini, Charissa: 9010
Hosseini Ghomi, Reza: 7300
Hoth, Karin: 7990
Hou, Anthony: 2220
Hou, Lisha: 7830
Hou, Su-I.: 2345, 3400
Hou, Xiaohui: 1165
Hough, Monica Strauss: 4415
Houghton, Frank: 7560
Hounsell, Cindy: 2025
Houser, Ari: 2395
Houston, Denise K.: 3505, 4355, 7630, 9010
Hovland, Cynthia A.: 4406, 7500
Howard, Elizabeth P.: 3095, 7160
Howard, Margret P.: 7890, 9010, 9030
Howard, Timothy D.: 5060
Howard, Virginia: 9000
Howe, Judy: 7000
Howe, Melissa: 1230
Howell, Britteny M.: 1365, 2245, 5200, 9010, 9020
Howell, Teresa F.: 7180, 9000
Howlett, Susan E.: 1060, 9010
Hoy-Ellis, Charles P.: 2310
Hoyumpa, Garielle: 7610
Hreha, Kimberly: 9030
Hsieh, Jessica: 7010, 9030
Hsieh, Ning: 4230
Hsieh, Yi Chin: 9030
Hsu, Amy P.: 7830
Hsu, Clarissa: 3490, 7810
Hsu, Erh-Chi: 4408, 7070, 7700
Hsu, Hui-chuan: 7990
Hsu, Kai-Wen: 4490
Hsu, Wan-Hsiang: 3365
Hsu, Wen-Chuin: 4155
Hsueh, Ming-Chun: 7930
Hu, Bo: 1130
Hu, Elise Z.: 4465
Hu, Jie: 7610
Hu, Jinyu: 9020
Hu, Lynn: 7760
Hu, Qing: 4155
Hu, Rita X.: 1255, 2100, 3290, 4285, 4325
Hu, Tiantian: 9010
Hu, Tsuey-Hwa: 9010
Hu, Xiyuan: 9030
Hu, Xujing: 9000
Hu, Yi-Ling: 7170
Hu, Yuanyuan: 7280, 7750
Hu, Yue: 9020
Hua, Cassandra L.: 1210, 3320
Hua, Yindong: 7680
Huang, Alison R.: 1295, 2135, 2240, 3335
Huang, Amy: 7710
Huang, Beicheng: 9010
Huang, Bin: 7300, 7830
Huang, Chiung-Kuei: 7390
Huang, Di: 9540
Huang, Hao: 9010
Huang, Hsien-Hao: 9030
Huang, Hsui-Li: 4155
Huang, Jiahui: 9030
Huang, Jian: 7370
Huang, Jing: 3500, 4310, 5175, 7070
Huang, Kian A.: 7890
Huang, Molly: 8900
Huang, Qiao: 9000, 9020
Huang, Sherri: 7870
Huang, wei-Jia: 4134, 7830
Huang, Xiuxiu: 7100, 7830
Huang, Yuan: 7210
Huang, Ziwei: 5180
Hubbard, Erin: 2280
Huber, Lesa: 4020
Huber, Zilla M.: 4225
Hubert, Terrence: 2295
Hucek, Ally: 1085
Huckfeldt, Peter: 3010, 4355
Hudson, Amy E.: 9000
Hueluer, Gizem: 4225
Huffman, Kim: 9000
Hugenschmidt, Christina: 5230
Hughes, Adriana: 7620
Hughes, Griffin: 7210
Hughes, Jaime M.: 3505, 4350
Hughes, Kelly L.: 3365
Hughes, M. Courtney: 4540, 7090
Hughes, MacKenzie L.: 1255, 7510, 7550
Hughes, Michael: 7590
Hughes, Michelle: 7480

Hughes, Susan: 7710
Hughes, Tiffany F.: 5080
Hughes, Tim M.: 7630
Hughto, Jaclyn MW.: 4000
Hui, Ferdinand: 2380
Hui, Jenny: 4340
Huisingh-Scheetz, Megan: 9030
Huisman, Martijn: 4485, 7360
Huisman - De Waal, Getty: 3340
Hulsebus, Lindsey: 7260
Hulsman, Erin: 7590
Humber, Marika B.: 4095
Humbert, Andrew T.: 9580
Hummel, Scott L.: 7210
Hunder, Allison: 9020
Hung, Anthony: 9030
Hung, Lillian: 2340, 3270, 3570, 9000, 9010, 9020
Hung, William W.: 2085
Hung, William: 2145, 7820, 9010
Hunt, Riley: 1375
Hunter, Alistair: 4285
Hunter, Melissa L.: 2375
Hunter, Paulette: 4470
Huntington-Symons, Makeba: 4010
Huo, Meng: 1050, 3425
Huo, Zhiguang: 7630
Huppert, Theodore J.: 2155
Hurd, Julie A.: 7910
Hurley, Jennifer: 4335
Hurwitz, Isabel: 9000
Huseth-Zosel, Andrea: 1235, 7710, 7810, 9030
Hussain, Naaz A.: 1035
Husser, Erica K.: 4315, 4525
Hutson, Jennifer: 8900
Hutto, Lashawn: 1070, 7330
Hutton Johnson, Stacy: 3535
Huynh, Duong: 7300
Hwang, Doyeon: 9020
Hwang, E. Shelley: 1370
Hwang, In Jeong: 4390
Hwang, Jing-Shiang: 9010
Hwang, Misun: 7570
Hwang, Phillip: 9010
Hwang, Sinwoo: 7460
Hwang, Ula: 2085
Hwang, Woosang: 4085
Hwang, Yeji: 7540
Hydén, Lars-Christer: 4190
Hyde, Cori D.: 7590
Hyde, Joan J.: 9030
Hyde, Martin: 5050, 7520
Hyun, Jinshil: 9020
Hyun, Kate: 7730

I

Iaboni, Andrea: 7080
Iacob, Eli: 1150, 2035, 4365, 9020
Iaconi, Alba: 4408, 7720
Ibáñez-Carrasco, Francisco: 4340
Ibrahim, Safa: 7540
Ice, Erin: 7470
Idler, Ellen L.: 4045
Igarashi, Ayumi: 7160, 9520
Igarashi, Heidi: 7580
Ignacio, Daniel: 7070
Ihara, Emily S.: 1335, 3310, 7230, 7750, 7760
Iijima, Katsuya: 3310, 9030
Ikeuchi, Tomoko: 7280
Iktilat, Khalil: 3125
Ilardo, Joan L.: 4155
Iliff, Jeffrey: 9000, 9010, 9020
Iloputaife, Ikechukwu: 9030
Im, Eun-Ok: 7220
Im, Yunju: 7510
Imai, Ryota: 9030
Imai, Shin-ichiro: 2205
Imaoka, Masakazu: 9030
Imms, Phoebe: 9020, 9030
Imuta, Hiromi: 7580
Inagaki, Asa: 9520
Inagaki, Hiroki: 7280
Infurna, Frank J.: 7610, 7950, 9010
Ingle, M. Pilar: 3410
Ingvalson, Stephanie N.: 2130, 3570
Inker, Jenny: 3300, 3425
Inloes, Jennifer: 2295
Inoue, Megumi: 1335, 3310, 7700, 7750
Insel, Kathleen C.: 1260
Intrator, Orna: 1005, 2145
Intrator, Orna: 9010, 9030
Inventor, Ben: 3445
Ioanitoaia-Chaudhry, Iulia: 4075
Ioannidis, George: 4268, 5100, 7680
Ipakchi, Narda: 2200
Iqbal, Anum: 4455
Irimia, Andrei: 9020, 9030
Irwin, Madison: 7670
Isaacowitz, Derek: 1105, 3195
Isaacs, Tanya: 9030
Isales, Carlos M.: 7370
Ishak, Emily: 2365
Islaya, Cesz: 7460
Ismael, Saifudeen: 9010
Ismail, Zahinoor: 7080
Israel, Sarah: 2040
Istvanek, Kathryn: 2170
Itabashi, Masako: 9030
Ito, Hiro: 9020
Ito, Kae: 7280
Iveniuk, James: 1230, 9010
Iwarsson, Susanne: 2340, 7380
Ix, Joachim H.: 9500
Izzetoglu, Meltem: 2155

J

Jääskeläinen, Tuija: 9000
Jablonski, Rita A.: 1075, 3545, 4145
Jaboob, Saida: 4025
Jacelon, Cynthia: 9010, 9030
Jackson, Amy L.: 4005, 9010
Jackson, Casey: 3395
Jackson, Chandra: 7870
Jackson, Eva MJ.: 3300
Jackson, Heide: 3005
Jackson, Kathleen J.: 5055
Jackson, Kathryn L.: 1255
Jackson, Lexie: 3015
Jackson, Piper: 9030
Jackson, Simone: 7670
Jackson, W. Clay: 9020
Jacob, Peter: 3570
Jacobs, David R.: 9010
Jacobs, Jeremy M.: 2015, 7300
Jacobs, Karen: 4125
Jacobs, M. Lindsey: 7390, 9010
Jacobs, Michael A.: 2180
Jacobs, Moyosoreoluwa O.: 7970, 9020
Jacobsen, Erin: 5080
Jacobson, Mireille: 3220, 4435, 5110
Jacques, Paul: 1290
Jaeger, Byron: 4134, 7830
Jaen, Carlos: 5055, 7660
Jaffee, Michael: 9010, 9020
Jaffri, Abbis: 7020
Jahn, Georg: 7070, 7590
Jaijairam, Emily: 9030
Jain, Shalini: 4475, 7100, 7870
James, Bryan D.: 2120, 2365, 3245
James, Dara: 5080
James, Dara: 7100
James, K. S.: 4385
Janevic, Mary R.: 7680
Jang, Aeji: 9100
Jang, Heejung: 1360
Jang, Jae Young: 9020
Jang, Jieun: 9520, 9540
Jang, Jinwoo: 5225
Jang, Jiyoon: 7420, 7720
Jang, Yuri: 2090, 7420, 7510, 7960, 9000, 9020
Jani, Niti: 8900
Janke, Megan: 7610
Jansen, Carl-Philipp: 3550
Jansen, Jacobus FA.: 1225
Jansen, Taylor: 4266, 7230, 7270, 7830
Janssen, Daniel: 9520
Janssen, Leah M.: 3190, 4435
Jao, Ying-Ling: 3020, 7160, 7620
Jarrín, Olga F.: 1240, 4105, 7800, 9000
Jarrett, Pamela: 7660, 7830
Jarrott, Shannon E.: 2140, 3385, 4085, 7590
Jason, Kendra: 7290
Javier, Noelle Marie: 4266, 7190
Jayakody, Oshadi: 3160
Jaykrishna, Poornima: 9020
Jeamjitvibool, Thanakrit: 4025
Jeanblanc, Alexandra: 2005, 3360, 7030, 9020
Jee, Sue: 9030
Jee, Youngmin: 9030
Jefferson, Rebeca C.: 9000
Jehu, Deborah: 9010, 9030, 9500
Jen, Sarah: 4195
Jenkins, August: 5130
Jennings, Lee: 4060, 9020
Jensen, Francine Bench: 3410
Jeon, Heesu J.: 9400
Jeon, Jehoon: 9010
Jeon, Sangha: 3135
Jeong, Chung Hyeon: 9010, 9020
Jeong, Haelim: 5125
Jeong, Innhee: 9000
Jeong, Jihyeong: 9010
Jeong, Kyu-Hyoung: 7030
Jeste, Dilip V.: 1390, 3565
Jester, Dylan J.: 7610
Ji, Linying: 1180, 3500, 4490
Ji, Wenyan: 9020
Jia, Maoni: 9110
Jia, Yuane: 4210, 7990

Jiang, Da: 4480, 4540, 7610, 7950
Jiang, Depeng: 7940
Jiang, Kening: 2135, 2240, 3335, 4265
Jiang, Lin: 4240, 9030, 9300
Jiang, Nisi: 4475
Jiang, Quanbao: 7530
Jiang, Xiangxiang: 9000
Jiang, Yan: 9550
Jiang, Yanping: 2090, 3035, 4050, 7080, 7600, 9020
Jiang, Yun: 1260, 7570
Jiang, Zhe: 4415
Jim, Heather: 7410
Jimenez, Daniel: 4340
Jimenez, Daniel R.: 9000
Jimenez, Sarah Y.: 5185
Jimenez Romero, Angélica: 9000
Jin, Bora: 7190
jin, Haiyu: 4090
Jin, Jessica: 7300, 7910, 9010, 9020
Jin, Shiow J.: 9010
Jindal, Shivani K.: 2145, 7860
Jing, Bocheng: 5230, 7160
Jiwani, Rozmin: 7180, 7910
Jobaidul, Boni: 7730
Joffe, Hadine: 5155
Johansson, Boo: 7660
John, Deepti Anna: 4540, 9010
Johnson, Abree: 3550
Johnson, Alisa J.: 4134, 7330
Johnson, Alisha H.: 7140
Johnson, Benjamin B.: 3250, 7590
Johnson, Christopher: 4155
Johnson, CJ L.: 4408, 7700
Johnson, Elma: 1150, 3240, 3300, 7380
Johnson, Eric: 5135
Johnson, Florence U.: 9020
Johnson, Ian M.: 3410
Johnson, Ian: 3410
Johnson, Ian M.: 5150
Johnson, James: 7110
Johnson, John C.: 7860
Johnson, Julene: 1150, 2075, 9020
Johnson, Justin: 5195
Johnson, Karen C.: 4355
Johnson, Kim G.: 9550
Johnson, Kimberly S.: 4165
Johnson, Kimson E.: 9030
Johnson, Melanie L.: 8900
Johnson, Michelle J.: 7860
Johnson, Min Kyoung: 2090, 9320
Johnson, Roxann H.: 4155
Johnson, Sterling C.: 2240
Johnson, Yuuko M.: 9030
Johnson Nicholson, Meneka C.: 9020
Johnson-Koenke, Rachel A.: 9020, 9030
Johnston, Carrie: 5115
Johnston, Laura: 9000
Joiner, Raquel J.: 1070, 4400
Jones, Alexandria: 7020
Jones, Bobby L.: 3500
Jones, Brittany R.: 7230
Jones, Christine D.: 7160
Jones, Elizabeth J.: 9420
Jones, Floyd A.: 4100
Jones, Garrett: 9000
Jones, Harlan P.: 3450
Jones, Holly: 7850
Jones, Judith: 4300, 7640
Jones, Leanne: 3070
Jones, Liz: 2375
Jones, Louise: 7160
Jones, Megan D.: 2015, 7810
Jones, Patricia: 3040, 1380
Jones, Sarah E.: 1315, 4415
Jones, Valerie: 4268, 7680
Jönson, Håkan: 5110, 7190
Joo, Jinhui: 4460
Joo, Susanna: 4370, 7610
Jopp, Daniela S.: 3130, 7080
Jopson, Andrew D.: 5160
Jordan, Tera R.: 7830
Joshi, Purnashree: 5075
Joshi, Suryakanth: 9010
Ju, Eunae: 4025
Juckett, Lisa: 9010
Judd, Suzanne: 9000
Judge, Katherine: 9020
Judon, Kimberly M.: 9000
Juengst, Shannon B.: 7650, 7760
Jump, Alayna: 7830
Jun, Jin: 7090
June, Andrea: 1080
Jung, Hailey H.: 2310
Jung, Haneul: 9020
Jung, Hye-Young: 5215
Jung, Jae Yeon: 7200
Jung, Sun Ok: 7160, 7800
Jung, Sunwoo: 9030
Jung, Wonkyung: 7150, 7280
Jung, Woo Seok J.: 9020
Jungermann, Joanna: 9030
Juon, Hee-Soon: 9440
Juratovac, Evanne: 9020
Juris, Jill: 3060, 4085
Jurkowski, Elaine T.: 7000
Justice, Jamie: 3290, 7370, 7630, 9010
Juszczyk, Ewelena: 4475
Jutkowitz, Eric: 2130, 3010, 3305, 4140
Jylhä, Marja: 9000
Jylhävä, Juulia: 9010

**K**

K. Nair, Anil: 9000
Kaasalainen, Sharon: 4440, 4470
Kaba, Diarratou: 4425
Kabaya, Sonoko: 9010
Kabayama, Mai: 4505, 9000
Kabeto, Mohammed U.: 1085
Kadam, Ashay: 7210
Kado, Deborah: 5085
Kaga, Hideyoshi: 9000
Kagai, Violet: 2010
Kahana, Eva: 3360, 3485, 7030
Kahana, Shoshana: 2270
Kahsay, Eskira: 3080
Kahya, Melike: 2155
Kaiksow, Farah: 4250
Kaitlin Casaletto, Shea Andrews: 2240
Kajitani, Shinya: 4385
Kakagawa, Kazuma: 5060
Kakehi, Saori: 9000
Kalaw, Karel: 4406, 7000
Kales, Helen: 7160
Kalesnikava, Viktoryia: 4545
Kallestrup, Peter: 3515
Kallmi, Selmi: 7340
Kalousova, Lucie: 4510
Kamath, Vidyulata: 2240
Kamide, Kei: 9000
Kamijo, Mio: 3310
Kan, Denise: 5125
Kan, Winnie: 3270
Kanagawa, Hotinpo Sky: 9420
Kananen, Laura: 9000, 9010
Kanani, Pushti: 7870
Kanaya, Reiko: 9000
Kane, Alice E.: 9020, 9030
Kane, Esther: 7420
Kane, Kevin: 2385
Kaner, Eileen: 9010
Kang, Bada: 7720, 7830, 9000, 9010
Kang, Chun-Ya Belinda: 7000
Kang, Edward: 9020
Kang, Ezer: 3440
Kang, Hun: 9020
Kang, Hyewon: 7070
Kang, Hyun: 3310, 4195
Kang, Jee eun: 1180
Kang, Mingon: 4075
Kang, Miseon: 7040, 7080, 7610
Kang, Suk-Young: 7760
Kang, Sunny: 7720
Kang, Yejin: 9310
Kang, Youjeong: 5055
Kanofsky, Chayna: 7550
Kantamneni, Leela: 4525
Kantarci, Kejal: 7100
Kanter, Jeremy: 1235
Kao, Hsueh-Fen S.: 5185
Kapahi, Pankaj: 4335, 9030
Kapogiannis, Dimitrios: 4260
Kapoor, Karina: 7900
Kappel, Rachel: 1065
Kapteyn, Arie: 3160
Karadimas, Thomas: 2325, 9330
Karagiannis, Tanya: 5090, 7370
Karakida, Maki: 9030
Karakose, Selin: 4055
Karam, Rita: 4345, 7040, 7380
Karasik, Rona J.: 2095, 4406, 7000
Kåreholt, Ingemar: 3470, 7040, 7180, 7300
Karel, Michele J.: 4095, 5135
Karibian, Bianca: 7800
Karikari, Grace: 3035, 7480
Karjadi, Cody: 7100, 9010
Karlamangla, Arun: 4120
Karlawish, Jason: 7650
Karlin, Nancy J.: 2125
Karliner, Leah: 3490
Karlsson, Ida K.: 9020
Karmacharya, Isha: 3045, 3190, 5220
karnam, Raghavi: 9010
Karney, Benjamin: 4400
Karpyn, Allison: 9020
Karraker, Amelia W.: 4205
Kartoz, Connie R.: 9000
Karvonen-Gutierrez, Carrie A.: 5155, 9010
Kasahara, Tomoko: 7370, 9010
Kasenetz, Whitney E.: 9010
Kaskie, Brian: 1030, 2175, 4435
Katagiri, Keiko: 3280, 7290, 7610
Katie, White: 3045
Katsev, Danielle: 4005
Katsumata, Asako: 7790
Katyarmal, Swati: 9000

Katz, Benjamin: 7020, 7100, 7830, 7990
Katz, Mindy: 3480, 5165, 9010, 9020
Katzel, Leslie I.: 4130
Kaufman, Brystana G.: 1020
Kaufman, Jennifer E.: 3565, 7810
Kaufmann, Christopher N.: 3500, 4490
Kaur, Simran: 3270
Kaur, Tripat: 4345
Kaushal, Navin: 5125
Kavalieratos, Dio: 1010, 3435
Kavanaugh, Melinda S.: 3020, 7910
Kavanaugh, Rachel: 7310
Kavcic, Voyko: 7510, 9020
Kawachi, Ichiro: 5130
Kawamori, Ryuzo: 9000
Kawasaki, Teruaki: 9010
Kay, Lilli: 7360
Kaya, Alaattin: 7370
Kayatta, Sophia: 7460
Kaye, Jeffrey A.: 2360, 3115, 3535, 4100, 4495, 7300, 7540, 7620, 7680
Kaysen, Anne: 9020
Kayser, Jay R.: 4325, 5235, 7030, 9010
Kazan, Joseph: 5175
Ke, Yemiao: 9320
Keane, Nicole: 7810
Keating, Norah C.: 7980
Keefe, Bronwyn: 4125, 7480
Keefe, Francis: 7410
Keefe, Janice M.: 2170, 4515
Keenan, Teresa: 5175
Keenoy, Jaclyn E.: 1185
Keesee, Francesca: 7760
Keinan, Amina: 7380
Keister, Melanie: 9020
Kelekar, Uma: 3220, 7210, 7830, 9010
Kelfve, Susanne: 4190, 4395, 4450
Keller, Ashley: 9020
Keller, Hallie E.: 7180, 9000
Keller, Heather: 7300
Keller-Ross, Manda L.: 9410
Kelley, Amy: 3040, 3185, 3415
Kelley, Casey J.: 7300
Kelley, Jessica: 2050, 2190, 7410
Kelley, Lynette: 9010
Kelley, Susan J.: 3060, 4400
Kelly, Debra: 9030
Kelly, Ronald: 7160
Kelz, Rachel: 7860
Kemeny, Betsy E.: 5065, 9410
Kemp, Blakelee: 7960
Kemp, Candace: 3285, 7290
Kendzor, Darla: 9030
Kennedy, Courtney: 4268, 5100, 7220, 7680
Kennedy, David: 2100
Kennedy, Katherine A.: 3320, 4290, 5215, 7160, 7770
Kennedy, Quinn: 1040
Kennedy, Richard: 9030
Kennedy, Teri: 1330, 5170, 7010
Kenten, Charlotte: 4470
Kenyon, DenYelle: 7600
Keogh, Alison: 3550
Kergoat, Marie-Jeanne: 9000
Kerr, Eleanor M.: 1000
Kerr, Kirk W.: 9020
Kerssens, Chantal: 7990
Kersten, Alan W.: 7510
Kervin, Lucy: 1335
Kerzner, Rachel: 7380
Kessler, Dorothy: 9000
Kew, Chung Lin (Novelle): 7760
Key, Kent: 1270
Keyes, Laura M.: 2315, 4150
Keys, Fay: 1005
Keys, Matthew T.: 4260
Kezios, Katrina: 9500
Khalaila, Rabia: 7480
Khalidi, Sarah: 4340
Khambaty, Tasneem: 4130
Khan, Karim M.: 5100
Khan, Maham: 5195
Khan, Mushira M.: 3035, 3440, 4285, 9000
Khan, Nibraas: 9030
Khan, Shehroz: 7190, 7680, 9000, 9330
Khan, Zain: 1110
Khanh Toan, Tran: 2050
Khanna, Saumya: 5220
Khattar, Jayati: 7660
Khavjou, Olga: 2375
Kheirbek, Raya E.: 4115, 4160, 4250
Khera, Alka: 7760
Khillan, Rohini: 5175
Kho, Minjung: 2320
Khong W. H., Andy: 3295
Khor, Ern Chern: 9030
Kianpoor, Kiarash: 7120
Kibildis, Samuel W.: 9030
Kidd, Lori: 7250
Kido, Michiko: 4505, 9000
Kieffer, Elana F.: 2315
Kiely, Kim M.: 3570
Kiely, Kim: 9000
Kikuchi, Kazunori: 7280
Kilaberia, Tina R.: 5170, 7280, 7750
Killian, Tim S.: 7070
Killingsworth, Derek C.: 7590, 7900
Kilpatrick, Sidonie: 5155
Kim, Andy: 7510
Kim, Benjamin: 9010
Kim, Boah: 2230
Kim, Boeun: 1345, 2225
Kim, Bohyun: 7610
Kim, BoRin: 4266, 7550, 7690, 9010, 9020, 9030
Kim, Chang Oh: 7660
Kim, Changhwan: 9020
Kim, Choong: 7980
Kim, Dae H.: 9410
Kim, Dae Hyun: 3565, 9010, 9520, 9540
Kim, Dahee L.: 1390
Kim, Eric S.: 2140, 9000
Kim, Eunbea: 3170, 3440
Kim, Eunhye: 7220
Kim, Eun-Kyeong: 3115
Kim, Eunyoung: 7400
Kim, Giyeon: 3245, 7510, 7560, 7610, 7830, 7940, 7960
Kim, Gwang Suk: 7320, 7660
Kim, Ha Neul: 1280, 2085, 9010
Kim, Ha Yul: 9540
Kim, Hansol: 7530
Kim, Heejung: 7080, 7220
Kim, Hosung: 4265
Kim, Hyein: 7220
Kim, Hyeji: 9030
Kim, Hyejin: 3445
Kim, Hyejin: 7900, 7960
Kim, Hyosin: 1240, 4105, 7800, 9000
Kim, Hyoun Kyoung: 7610
Kim, Hyunji: 7500
Kim, Hyun-Jun: 2310, 4195
Kim, Jae: 9010
Kim, Jaehyun: 7910
Kim, Jeehoon: 7610
Kim, Jennifer (Jeehyun): 7830, 9000, 9010
Kim, Jeungkun: 7760, 9030
Kim, Jinhak: 9020
Kim, Jinho: 7330
Kim, JinShil: 7850
Kim, Jinyoung: 4134, 4265, 7330
Kim, Jiyeon: 7230
Kim, Jongwoong: 9010
Kim, Joohyun: 3130
Kim, Ju-Hyun: 7590
Kim, Julie: 4025
Kim, Jung Eun: 7160, 7800
Kim, Jung Ki: 2325, 4395
Kim, Jung Lyun: 9030
Kim, Jungsug: 7330
Kim, Junhyoung: 7910, 7990
Kim, Junhyoung: 9010, 9030
Kim, Kwang-il: 9000
Kim, Kyeongmo: 7250
Kim, Kyungmin: 1050, 3130, 3165, 3425, 7030, 7380, 7510, 7520, 9000, 9010
Kim, Laurie: 4075
Kim, Layoung: 7320
Kim, Man Su: 7880
Kim, Meeryoung: 7070
Kim, Miji: 7630, 9020
Kim, Min Hee: 2300
Kim, Min Jung: 7420, 7720
Kim, Minju: 7550
Kim, Miri: 7030
Kim, Miyong T.: 7420, 7510
Kim, Nahyun: 7030, 7380
Kim, Nahyun: 7610
Kim, Narae: 5110
Kim, Nayeon: 7300
Kim, Nicholas: 9020, 9030
Kim, Patricia: 2235
Kim, Pearl: 4075
Kim, Rockli: 7320
Kim, Samsuk: 3155
Kim, Seon: 7230
Kim, Seonghoon: 7960
Kim, Seonjin: 9010
Kim, Seoyoun: 4210, 4255
Kim, Sharon H.: 7510
Kim, Sohyun: 3020, 9030
Kim, Steffi M.: 2120, 3015
Kim, Sue: 7660
Kim, Sunghee: 7030
Kim, Wonjun: 9000
Kim, Yeoju: 7830
Kim, Yesol: 7080
Kim, Yijung K.: 3130
Kim, Yongseop: 7910, 9010, 9030
Kim, Yoon Chung: 5020, 9030
Kim, Youjin: 7030
Kim, Youn Kyoung (Lily): 9020
Kim, Young Sun: 9540
Kim, Yulri: 3360
Kim, Yun Joong: 7400
Kimball, Patricia F.: 3375, 4215

Kim-Knauss, Yaeji: 3100, 7900
Kimpel, Christine C.: 9020
Kimzey, Michelle: 2080, 4220
Kincaid, Reilly: 1255
Kindratt, Tiffany B.: 3440
King, Angelique: 3250
King, Barbara: 1190
King, Deborah A.: 3490
King, Jessica: 4165
King, Natalie: 2375
Kinkade, Emily: 1235, 5035, 7710
Kinney, Anita: 7410
Kinney, Jennifer M.: 4435, 7140, 7540
Kinoshita, Ayae: 9010
Kiosses, Dimitris: 3520
Kip, Stephanie: 3560
Kircher, Julie A.: 9020
Kirchner, Jessica: 2375
Kiriazova, Tetiana: 2055
Kirk, Allan D.: 1370
Kirkham, Heather: 9020
Kirkwood, Renata: 4268, 7680
Kissell, Paul: 1150, 3240
Kitaigorodsky, Marcela: 4340
Kitt-Lewis, Erin: 9010, 9540
Kitzman, Dalane W.: 3505
Kiyoshi-Teo, Hiroko: 5205
Klaiber, Patrick: 3135, 3520, 3545, 4320
Klee, Matthias: 1225
Kleijwegt, Hannah: 3415
Kleissner, Verena: 7590
Klenk, Jochen: 3550
Klepacz, Laura M.: 9000
Kleszynski, Keith: 2215, 9020
Kline, Chelsea R.: 1265, 7420
Klinedinst, Jennifer: 2210
Klinedinst, Tara C.: 9030
Klingman, Karen J.: 2110
Klopack, Eric T.: 1325, 2325
Kloss, Katie: 1030
Knapp, David: 3160
Knebl, Janice: 4220, 4360
Knecht-Fredo, Jenny L.: 4525
Knepple Carney, Amy: 7390
Knight, Erik: 9030
Knighton, Amy: 7830
Knill, Samantha: 7830
Knol, Linda L.: 7620, 9000, 9020
Knopp, Kasey: 7170
Knox, Melissa L.: 9020
Knox, Patrick: 7410
Ko, Ahra: 9030
Ko, Eunjung: 7090
Ko, Fred: 7000
Ko, Fred: 7150, 7820, 7860
Ko, Myungjin: 9030
Ko, Soohyeon: 7320
Ko, Su-Hyuk: 8900
Ko, Sung Hyun: 2375, 7770
Kobayashi, Erika: 4225
Kobayashi, Lindsay: 1085, 1170, 2280, 3290, 7320, 7940
Kobsar, Dylan: 9020
Kobylarz, Fred: 4105, 9000
Koch, Mary Kate: 7380, 7580, 7900
Koehler, Sebastian: 1225
Koehly, Laura: 7070
Koetaka, Hanayo: 9000
Koeuth, Sokha: 2130
Koffer, Rachel E.: 3135
Koffski, Maya J.: 3300, 3570
Koh, Young Bin: 9010
Kohanski, Ronald A.: 3040
Kohl, Raphael: 9400
Kohlbrenner, Briana: 5200
Kohler, Hans-Peter: 9020
Kohli, Nidhi: 5170
Kohon, Jacklyn N.: 2105, 3220
Koirala, Binu: 7770, 7840, 7960
Koizumi, Naoru: 7700
Kok, Almar: 4485, 7360
Kolade, Opeyemi R.: 1175, 4255
Kolbila, Lilian: 7790
Koletar, Susan L.: 3390
Kolind, Shannon: 3495
Koller, Jeanne M.: 7450
Kolpakov Nikitin, Stanislav: 5015, 7100
Komalasari, Renata: 7830, 9000, 9010, 9020
Kondrikov, Dmitry: 9200
Kondrikova, Galina: 9200
Konetzka, R.Tamara: 1115, 4140
Kong, Dexia: 4540, 7940, 7950, 9020
Kong, Eun-Hi: 7300, 9020
Kong, Jooyoung: 1130, 3170, 7050
Kong, Lan: 5145
Kong, Xiangjing: 2265, 7330
Kono, Sei: 9030
Kononowech, Jennifer: 7160
Koon, Lyndsie: 9030
Kopera-Frye, Karen F.: 4268, 7680, 7980
Korc-Grodzicki, Beatriz: 7410
Koren, Chaya: 3170
Korfage, Ida J.: 4440
Korfhage, Justin: 1060
Korinek, Kim: 2050
Kornadt, Anna E.: 3100, 4180
Koronowski, Kevin: 9000
Kosar, Cyrus: 5215
Koschate, Jessica: 7830
Koskinen, Seppo: 9000
Kossowska-Kuhn, Dorota: 1315, 9000, 9030
Koster, Annemarie: 1225
Kostyniuk, Lidia: 9030
Koszalinski, Rebecca S.: 2180
Kotarba, Joseph A.: 4040
Kotecho, Messay: 2010
Koternamski, April: 7640
Kotwal, Ashwin: 4205, 7610, 9020
Koubasi, Farnaz: 9000, 9020
Koumoutzis, Athena: 4425
Kouroukis, Alexa: 4268, 7220, 7680
Kovacs, Laszlo: 9030
Kowalchik, Kalei H.: 5030
Kowdley, Gopal: 7860
Kozlov, Elissa: 4305
Krämer, Michael: 9010
Krafty, Robert: 5175
Kramer, Arthur: 7100
Kramer, Jenna: 4345, 7040, 7380
Kramer, Josea: 3250, 4095
Kramer, Philip: 3475, 5210
Kramsky, Jayna: 9010
Kraschnewski, Jennifer: 5030, 5145
Krase, Kathryn: 9030
Kratzer, Andre: 2370
Kraus, William: 9000
Kravchenko, Julia: 3510, 5015, 5110, 7620
Kravitz, Howard M.: 5155
Kravljaca, Nikolina: 7040, 7520
Kreitzer, Natalie: 7850
Krendl, Anne: 9300
Kress, Taylor: 9030
Krieg, Carsten: 9200
Kringos, Dionne S.: 9010
Kristen, Jacobs: 3425
Kritchevsky, Stephen B.: 2155, 3475, 5210, 5230, 7630
Kritz-Silverstein, Donna: 9010
Kroeck, Mallory: 7890, 7990
Krok-Schoen, Jessica L.: 2260
Kropp, Denise J.: 7250, 7480
Kruahong, Suratsawadee: 9020
Krueger, Rejko: 1225, 9020
Krukowski, Rebecca: 9020
Krupp, Karl: 9010, 9020
Krygowska-Nowak, Natalia: 4450
Krystina, Lewis: 9000
Kuball, Tim: 7070, 7590
Kubasova, Ksenia: 4266, 7190
Kubo, Takanari: 9030
Kucharska-Newton, Anna: 5085, 7340
Kuchel, George A.: 4485
Kuczmarski, Marie Fanelli: 9000
Kueht, Michael: 7860
Kuffel, Randall L.: 2280
Kuhlmey, Adelheid: 9000, 9400
Kuipers, Allison L.: 5090, 9020
Kulati, Ellam: 1220
Kulatunga, Senara: 9010
Kulkarni, Snehal S.: 7820
Kulminski, Alexander: 4260
Kum, Cleopatra: 7850
Kumar, Raj: 7650
Kumar, Sidharth: 7680
Kummerlowe, Megan: 7330
Kuna, Samuel T.: 7180
Kung, Claryn S J.: 4015
Kunik, Mark E.: 1280, 7850
Kunkel, Suzanne: 3045
Kunonga, Tafadzwa Patience: 4455
Kunz Lomelin, Alan: 3305, 4420
Kuo, Fang-Lin: 7910
Kuo, Jasmin: 9030
Kuo, Pei-Lun: 2195
Kuo, Tony: 7700
Kuo, Tsuann: 2345
Kuo, Yong-Fang: 4395, 9010
Kurth, Jordan D.: 9010, 9020
Kurth, Maria: 1160, 7090, 7510, 7580
Kusmaul, Nancy: 1020, 1100, 2020, 3150, 5020, 9000, 9010, 9100
Kuspinar, Ayse: 5070

Kusters, Cynthia D.: 5225, 7370
Kuzmik, Ashley: 1300, 2305
Kvale, Elizabeth: 3345
Kwak, Jung: 3345, 4000
Kwan, Thomas: 7390
Kwas, Stephen: 9020
Kwok, Alex Pak-Ki: 9030, 9510
Kwok, Amy: 9400
Kwok, Jojo Yan Yan: 7610
Kwok, Stanley: 7120
Kwok, Timothy: 1145
Kwon, Eunsun: 9010
Kwon, Jenny: 7010, 7590, 9010, 9030
Kwon, Sohyeon: 3390
Kwon, Soonhyung: 4530, 5115, 7920, 9020
Kylén, Maya: 2340, 7380

L
LaBlanc, Raeann: 9010
Labyak, Corinne: 7870
Lach, Helen W.: 7150
Lachman, Margie E.: 1360, 7380, 7510, 7970
Lachs, Mark S.: 1145, 2060
Lackmeyer, Abbe: 3045
LaCroix, Andrea Z.: 3390
Lacy, Briana: 9310
Ladowski, Kristi: 9020
Ladwig, Matthew A.: 9010, 9020
Laffer, Alexandra: 9020
Laffitte Nodarse, Chavier: 9010
Lafontant, Kworweinski: 9010, 9020
Lafountain, Olivia: 4195
Lagacé, Martine: 1200
Lagoo-Deenadayalan, Sandhya: 1370
LaHue, Sara: 1380
Lahvic, Jamie: 2270
Lai, Daniel W.L.: 1145, 7040, 7520
Lai, Francisco: 2280
Lai, Patrick HL.: 3095
Lai, Philip: 3530
Lai, Ting-Fu: 7930
Lai, Wen-Hua "Zoey": 1180
Lakatta, Edward: 9020
Lakshminarayan, Kamakshi: 3460
Lalji, Kahir: 2020, 5215, 7730
Lam, Gigi: 9030, 9510
Lam, Justin W.: 7750
Lam, Kenneth: 7180
Lamb, Raza H.: 3020
Lambert, Frederick: 9010
Lambert, Olivia J.: 4425
Lambrou, Nickolas: 3015
Lamerato, Lois: 2225
LaMonte, Michael J.: 3390
Lampe, Nik M.: 4000
Lampkin, Cheryl: 5040
Lampraki, Charikleia: 7080
Lancaster, Caroline: 3075
Lanctot, Krista: 7080
Landes, Scott: 1160
Landi, Francesco: 9020
Landman, Bennett A.: 2195
Landreville, Philippe: 7030
Lane, Amber: 7160
Lane, Annalise: 4545, 5050
Lane, Sandi J.: 9530
Lang, Barbara: 9010, 9020
Lang, Frieder R.: 3100, 7900
Lang, Justin: 5070
Lang, Samantha: 9020
Langa, Kenneth: 2280, 7320, 7650, 9110
Lange Maia, Brittney S.: 2120
Langefeld, Carl: 5060
Langendam, Miranda: 9010
Langsetmo, Lisa: 5085
Langston, Emily M.: 9000
Langston, Ryan: 9010
Langton, Lynn: 3075
Lanzi, Alyssa M.: 7620
Lao, Jenna: 7590
Lao, Jiahui: 9000
Lapidus, Jodi: 7110
Larkin, Jennie: 3040, 9030
Larkina, Marina: 9010, 9030
LaRoche, Dain P.: 5075, 9010, 9550
Larsen, Pernille S.: 4260
Larson, Janet: 7510
Laskow, Thomas: 1375
Lassell, Rebecca KF.: 7230, 9000
Latham, Nancy: 3480, 9000
Latham-Mintus, Kenzie: 4115, 4250
Lathren, Christine: 3320
Latimer, Abigail: 9000
Latkin, Carl: 9420
Latshaw, Emily: 9030
Lau, Bobo Hi-po: 3555, 9030, 9510
Lau, Gary K.K.: 7330, 7520
Lau, Terence K.: 4515, 7330
Lauber, Mckenzie S.: 7760
Lauderdale, Jana: 9020
Laumann, Edward: 1230
Laura, Bradbury: 1150
Laures-Gore, Jacqueline: 7500
Laurienti, Paul J.: 5230
Lavender, Kathryn: 7380
Lawrence, Jourdyn A.: 5130
Lawrence, Karen A.: 9020
Lawrence, Sacheen: 3015
Laws, Holly: 7420
Lay, Jennifer C.: 3275, 4480
Layman, Shannon: 1335
Layne, Diana: 9000
Laynor, Gregory: 7640
Laz, Mike: 2355
Le, Giang: 9530
Le, Qun: 1350, 2385, 3155
Leach, Carrie: 1270, 5030
Leahy, Suzanne: 9020
LeBrasseur, Nathan: 1325
Lech, Sonia: 9400
Leckie, Carolyn: 4268, 7220, 7680
Lecours, Alexandra: 9020
Lecvy, Nadine: 4460
Ledermann, Thomas: 4055
Lee, Ahyoung: 7590, 7890
Lee, Chae Man: 1240, 4140
Lee, Chae Man: 4266, 7230
Lee, Chae Man: 7830
Lee, Chi-Ko: 2230, 4020, 7040
Lee, Christopher S.: 3535
Lee, Chung-Sheng: 4134, 7830, 7930
Lee, Edwin H.M.: 7330
Lee, Eunju: 7520
Lee, Gina: 3440, 3555, 7610
Lee, Haena: 1230, 1320, 9030, 9570
Lee, Han Na: 9020
Lee, Hee Yun: 3005, 7380
Lee, Heejung: 7810
Lee, Hye Soo: 1120, 7680
Lee, Hyo Jung: 7080, 7960
Lee, Hyun Ju: 9540
Lee, Ilhak: 7900, 7960
Lee, Jae Jun: 7320, 7760
Lee, Jeannie K.: 1260
Lee, Jeenkyoung: 7550
Lee, Jennifer: 1290
Lee, Jennifer A.: 2035
Lee, Jenny: 9030
Lee, Jeong Eun: 1055, 2075, 3170, 3360, 3440, 3445, 4390, 7610
Lee, Ji Hyun: 1170, 4325
Lee, Ji Yeon: 7160, 7420, 7660
Lee, Jihui: 4420
Lee, Jihye: 4370
Lee, Jina: 4020, 4500
Lee, Jinkook: 9110
Lee, John Chi Kin: 7950
Lee, Jooah: 9010
Lee, Jooseon: 7810
Lee, Joowon: 7910
Lee, Joseph H.: 9020
Lee, JuHee: 7400
Lee, Juhyeong: 7510, 7940
Lee, Jung Wan: 9540
Lee, Jung-Ah: 4025, 7850
Lee, Jungjoo: 7990, 9030
Lee, Jungup: 7080
Lee, Kathy: 2330, 4420, 7730, 7910, 9010
Lee, Kayoung: 9020
Lee, Knoo: 1310
Lee, Kyu Ri: 5070
Lee, Kyung Hee: 7160, 7660, 7720, 9320
Lee, Kyungmi: 7680, 9030
Lee, Lewina O.: 3155
Lee, Lewis H.: 3005, 5125
Lee, Matthew R.: 5005, 7390
Lee, Milae: 7940
Lee, Moon: 1345
Lee, Nur 'Atiqah: 3435
Lee, Othelia: 3345
Lee, Sei: 3490
Lee, Seolah: 7080, 7960
Lee, Seonhwa: 4400
Lee, Seoyoon J.: 7030, 7380
Lee, SeungCheor: 7380
Lee, Soohee: 9020
Lee, Soo-Kyoung: 9030
Lee, Soomi: 3135, 3500, 4490, 5045, 7410, 7890, 7990
Lee, Sun Ah: 3135, 5045
Lee, Sunghoon I.: 9030
Lee, Sunmin: 2275
Lee, Susan: 1120
Lee, Tou S.: 3450
Lee, Vincent W.P.: 7040, 7520
Lee, Wei: 9030
Lee, Wei-Chen: 7310
Lee, Won: 7900, 7960
Lee, Yeonjung: 2375, 7770, 9010, 9030, 9100, 9420
Lee, Yeonjung Lee: 2230
Lee, Yeon-Shim: 7600
Lee, Yeonwoo: 9030
Lee, Yeseul: 7610
Lee, Youjung: 1005, 2005, 3060
Lee, Yura: 3035, 7610, 7640
Leedahl, Skye N.: 1350, 3385, 4085, 4375
Lees Haggerty, Kristin: 2030
Lefebvre, Emily L.: 7550
Leger, Kate: 5045, 7040

Leggett, Amanda N.: 1340, 1355, 7090
Leggett, Amanda N.: 9030
Lehman, Erik: 5145
Lehmberg, Lisa J.: 2390
Lehnbom, Elin C.: 9030
Lehning, Amanda J.: 9010
Lehpamer, Nicole: 5125, 7190
Lehr, Eric: 7170
Lei, Xiao Ying: 7420, 9030
Leis, Aleda M.: 5155
Leiser, Scott F.: 7880
Leishman, Eppie: 9000
Leist, Anja K.: 1225
Leite, Mateus Medeiros: 7340
Lekhak, Nirmala: 3485, 5010, 9010
Lekwauwa, Ruby: 4145
Leland, Natalie: 7360
LeLaurin, Jennifer H.: 2170
Lemieux, Catherine: 9020
Lendon, Jessica: 1095
Lengacher, Cecile A.: 3420
Lentz, Trevor: 7410
Leon Guerrero, Cierra: 4495
Leong, I tek: 2090, 9320
Lepore, Michael: 3180, 5020, 7300, 9020, 9030
Lerch, Ondrej: 7420
Lercher, Kirk: 7650
Lerner, Susan: 7820
Lervisit, Vina: 7730
Leshchyk, Anastasia: 7370
Leskinen, Tuija: 7170
Leslie, Kathleen: 7190
Lesperance, Larry: 9000
Lessenich, Stephan: 9030
Letendre, Scott: 4340
Letts, Elyse: 1280
Leung, Angela Y. M.: 4132, 4515, 7330, 7480, 7760
Leung, Dara Kiu Yi: 7760
Leung, Doris Y. P.: 4515
Leung, Isaac Sze Him: 7150
Leung, Lok Man: 9010
Leung, Verona Ji Ying: 7060
Leutwyler, Heather C.: 2280
Levan, Darius T.: 1305
Levasseur, Melanie: 1200, 7620, 7660
Levchenko, Yuliana: 9100
Leveille, Suzanne: 9000
Levenson, Robert W.: 1305, 4495, 9320
Leverentz, Andrea: 9000
Levin, Bonnie: 4340
Levin, Bruce L.: 4010
Levine, Adele: 2145
Levine, Deborah: 1085
Levine Coley, Rebekah: 3095
Levkoff, Sue E.: 2330, 9010
Levy, Becca R.: 3325
Levy, Cari: 1160, 4408, 7160, 7300, 7720
Lewin-Epstein, Noah: 9030
Lewinson, Terri: 5030
Lewis, Beth A.: 5100
Lewis, Brian: 9020
Lewis, Charity: 4110, 7960
Lewis, Constance: 4408, 7230
Lewis, Cora E(: 9010
Lewis, Hannah M.: 7010
Lewis, Jordan P.: 1140, 1270, 2120, 3015, 3200
Lewis, Nathan A.: 9000
Lewis, Penny J.: 4110
Lewis, Stevie: 9010, 9030
Lewis, Stuart: 3375, 4215
Lewis-Thames, Marquita W.: 2355
Lewy, Hailey: 7210
Leykum, Luci: 9010, 9030
Li, Chien-Ching: 3440, 7410, 7420, 9030
Li, Chihua: 2285, 3225, 9030
Li, Chih-Ying: 4395
Li, Chin-Shang: 7620
Li, Claudia: 5115
Li, Cun: 9000
Li, Daisy: 3280
Li, Daneng: 7410
Li, Guohua: 9010, 9020
Li, Jia: 7520
Li, Jiejin: 2145
Li, Jinfeng: 7070
Li, Jingxi: 7770
Li, Juan: 2265, 7330
LI, Junxin: 3500, 4310, 7070, 9300, 9320
Li, Ke: 4050
Li, Lun: 2230
Li, Lydia: 4325
Li, Martina: 3525
Li, Meiyi: 1230
Li, Mengchi: 3500, 7070, 9320
Li, Mengze: 5090, 7370
Li, Minghui: 9000
Li, Polly WC.: 5225, 7300
Li, Tao: 9000
Li, Wenjun: 1350, 2385, 3155, 5075, 7430
Li, Xia: 4240
Li, Xiaoang: 7870
Li, Xiaoli: 3380
Li, Xiaoling: 2205
Li, Xiaoqian: 4185
Li, Xinjun: 9000
Li, Xuelian: 4535
Li, Yanling: 7510
Li, Yawen: 1095
Li, Yaya: 4505, 9000
Li, Yiang: 1230, 4205
Li, Yichao: 7780
Li, Yixia: 2280, 4115
Li, Yongmei: 5230, 7160
Li, Yue: 4140
Li, Yuekang: 9020
Li, Yuelin: 7660
Li, Yuxiao: 7350
Li, Zhigang: 1030
Lian, James: 9000
Liang, Su-Ying: 3525
Liang, Xiaohui: 4268, 7680
Liang, Yuanyuan: 9000
Liao, Hsiao-Wen: 1320, 7070
Liao, Jing: 2255
Liao, Joshua: 7830
Liao, Pei-Chun: 7910
Liao, Shu: 9020
Liao, Shuyao: 9010
Liao, Yo-Jen K.: 3020, 7160, 7620
Liao, Yung: 7930
Liao, Zih-Yong: 7910
Libon, David J.: 7100
Lichtenberg, Peter A.: 3090, 7930, 9020
Liddle, Jennifer: 4540, 7160, 9010
Lieber, Sarah B.: 4268, 7680
Liebman, Michael: 3530, 9030
Liebreich, Robert: 7760
Liebzeit, Daniel J.: 4025
Liegey-Dougall, Angela: 7730
Liehr, Patricia: 5175
Lifshitz, Rinat: 7720, 7920
Light, Michael A.: 3410
Lighthall, Nichole R.: 7080, 7430, 9010
Ligon, Mary: 9000
Lilly, Flavius R W.: 3310
Lim, Aaron: 4275
Lim, Arum: 7770, 7840, 7960
Lim, Crystal: 1070
Lim, Diane: 4265
Lim, Emily: 4230
Lim, Jie Xin: 3085
Lim, Junghee: 7330
Lim, Junyub: 7610
Lim, Sungwon: 7150
Lim, Young Mi: 7330
Lima, Luciano Ramos: 7130
Lim-Soh, Jeremy: 3435
Lin, Feng: 9200
Lin, Frank R.: 2135, 2240, 2365, 4265
Lin, Gloria: 3280, 9010
Lin, Haiqun: 1240, 4105, 7800, 9000
Lin, Honghuang: 9010
Lin, Hongmei: 7060
Lin, Hsien-Ho: 9010
Lin, Hui-Yi: 7390, 7410
Lin, I-Fen: 1340
Lin, Jielu: 7070
Lin, Kun-Pei: 4134, 7830, 7930
Lin, Lisha: 7630
Lin, Rose: 5185
Lin, Shih-Yin: 2070, 3205, 7230, 7460
Lin, Shiow (Rosa): 5090
Lin, Shujing: 9580
Lin, Taiping: 7660
Lin, Tian: 7610, 9010, 9200
Lin, Wei-Ting: 7390
Lin, Xin Yao: 7610
Lin, Yan: 2250
Lin, Yi: 7870
Lin, Zhixuan: 3400
Lin, Zhiyong: 3105
Lin, Zhuoer: 1165, 2225, 2300, 3230, 3450
Lincoln, Karen D.: 2275, 3005
Lind, Majse: 7970
Lindauer, Allison: 4100, 4495, 7440, 7700
Lindberg, Brian W.: 2025, 2165, 2297, 3315, 3455, 4160
Lindell, Kathleen: 9000
Lindfors, Petra: 9030
Lindgren, Finn: 9020
Lindholm, Cassie: 9000
Lindquist, Lee A.: 7440, 7460, 7520, 9030
Lindsay, Rebecca: 7680
Lingler, Jennifer H.: 4210, 9020
Liou, Chih-ling: 4406, 7000, 7380
Liou, Shyhnan: 3100, 9030
Liougas, Madalena P.: 4515
Lipat, Ania: 9010
Lippe, Megan: 3005
Lipsitz, Lewis A.: 2155, 2190, 2320, 9030
Lipton, Michael L.: 1125
Lipton, Richard: 9010, 9020
Lister, Adrian: 9010
Lister, Jennifer J.: 7330
Liston, Dorion: 1040
Little, Deborah M.: 7010
Little, Natalie: 7150

Litzelman, Kristin: 7590
Liu, Bochu: 9000
Liu, Chang: 1130, 4020, 4520, 7090
Liu, Chiung-ju: 4380, 7170
Liu, Christine: 9010
Liu, Ethan J.: 9000, 9030, 9530
Liu, Fangyu: 1370, 5085, 7110
Liu, Hui (Cathy): 3105, 4230, 4370
Liu, I-Ting: 9000
Liu, Jie: 9210
Liu, Jingwen: 3515
Liu, Jinyu: 3305, 4050
Liu, Kathy Y.: 1215
Liu, Kehan: 4310
Liu, Lan: 9000, 9210
Liu, Li: 9000
Liu, Li-Chuan: 7780
Liu, Li-Fan: 2345, 3400, 9010
Liu, Lili: 1165, 1280, 5015
Liu, Minhui: 4310
Liu, Molei: 1110
Liu, Mona: 7540
Liu, Pi-Ju: 4215, 7790
Liu, Ruotong Mona: 3005
Liu, Siqi: 9000
Liu, Tianyin: 7760, 7920, 9420
Liu, Weichu: 4535
Liu, Wen: 3020, 5015, 5070, 9020
Liu, Wenlong: 3510
Liu, Xiaojuan: 4515, 5230, 7160
Liu, Xiaoyue: 7840, 7960
Liu, Xinran: 9020
Liu, Yan: 9540
Liu, Yanchen: 7620
Liu, Yaqian: 4132, 7480
Liu, Yin: 4370, 7400, 7940, 7990
Liu, Ying: 9200
Liu, Yixiu: 7940
Liu, Yuezhong: 3295, 3470
Liu, Yujun: 4540, 7090
Liu, Yunda: 9030
Liu, Yuqi: 3115
Liu, Yuyang: 2255
Liu, Zhijian: 2265, 7330
Liu, Zhilin: 9000
Liu-Ambrose, Teresa: 5100
Livingston, Gill: 4515
Livingston, Marin J.: 5200
Livingston, Rachel: 9000
Lloyd, Althea: 4095
Lo, Maggie Wai-Shan: 9420
Lo, Margaret C.: 1030
Lo, On-Yee: 2155, 7170
Lo, Wei-Cheng: 9010
Lo, Yu-Tai: 9030
Lobanov-Rostovsky, Sophia: 2255
Lobo, Judith D.: 4340
Locascio, Joseph: 4460
Loccisano, Elisabeth: 9010
Lo-Ciganic, Wei-Hsuan: 3500
Locke, Jenna R.: 4525
Löckenhoff, Corinna E.: 1090, 7510
Locklear, Clarence E.: 7460
Loerzel, Victoria: 7080, 7150, 7430
Loewenstein, David: 9010
Lofton, Jason: 9000
Logan, Ayaba: 9000
Logue, Cailie I.: 7830
Logue Cook, Rachel N.: 7640, 7820
Lohmar, Sabine: 7030, 7570
Lombard, David B.: 1060
Lomeli, Sindy: 4375, 7750
London, Andrew S.: 1160, 7970
Long, Monica: 9010
Long-Foley, Kristie: 7270
Longobardi, Isaac: 3175
Longoria, Denise: 4240
Longstreth, William T.: 9500
Lopez, Acacia: 7950
Lopez, Alexa: 7540
Lopez, Bruna: 9010
López, Christian: 7590
Lopez, Escarlata: 7320
Lopez, Janice: 7620
Lopez, Maria J.: 1240, 4105, 9000
Lopez, Oscar L.: 4080, 9500
Lopez, Ruth Palan: 4525
López Anuarbe, Mónika: 1115
LoPilato, Danielle M.: 1350
Lopresti, Brian J.: 9500
Lor, Maichou: 3450
Lord, Justin: 2350, 3365, 4190
Lord, Marie-Michele: 9030
Lorelli, Lisa: 4525
Lorenz, Rebecca A.: 7830
Lou, Vivian Weiqun: 1145, 3515
Lou, Yifan: 3305, 4050, 7260
Louras, Peter: 9010
Loureiro, Joao M.: 9020
Louwagie, Katie W.: 3305, 3570, 7380, 7420
Lovasi, Gina S.: 3405
Lovato, James: 4134, 7830
Love, Jill W.: 9030
Lovegrove, Peter J.: 2220
Lovett, Hephzibah: 3245
Low, Hilary: 7160
Lowe, Enya: 3220
Lowenkron, Jeff: 9000, 9010
Lowensen, Kelly: 1375
Lowers, Jane: 1010, 3435
Lowndes, Ashley: 5105
Lox, Dennis M.: 9000
Loyd, Christine M.: 9000
Lozinski, Tristen: 3520
Lu, Feng-Ping: 4134, 7830
Lu, Hongzhou: 7140, 7330
Lu, Jiehua: 4155
Lu, Kevin: 9000
Lu, Minjie: 4480, 7050
Lu, Peiyi: 4540, 7940, 9020, 9030, 9500
Lu, Shiyu: 3115, 3515, 4255
Lu, Yi: 1090
Lu, Yidan: 2150
Lu, Yueqi: 9000
Lu, Yvonne: 9400, 9440
Lu, Zhaohui: 1095
Luanda, Natália: 7340
Lubben, James: 2135
Lubetsky, Sara: 7850
Luborsky, Mark R.: 4300
Lucas, Alexandre: 2320
Lucero, Robert J.: 7860
Luchetti, Martina: 3245, 4035, 4055, 7990
Luciano, Michelle: 4134, 7620
Lucius-Milliman, Darby: 1315
Ludwig, Lindsay: 4350
Luebke, Matthew: 1145, 2295
Lui, Fan Yin-Fong: 9030, 9510
Lui, Li-Yung: 4260
Luk, Calvin W.: 7040
Luk-Jones, Susanna: 5190
Lum, Hillary: 9000, 9020
Lum, Terry: 3115, 3515, 4255, 7330, 7760, 7920, 9420
Lumey, Lambert: 2285
Lund, Jennifer L.: 7300
Lunetta, Kathryn L.: 7640, 7830, 9000
Luo, Hao: 2280, 3490, 3510, 7330
Luo, Jing: 1255
Luo, Jun: 7230
Luo, Minxia: 3115
Luo, Weixiang: 9020, 9520, 9540
Luo, Yan: 4310
Luo, Ye: 7960
Luo, Yuqian: 4310
Luong, Gloria: 5035
Lüscher, Janina: 4235
Luskey, Valerie E.: 9010
Lustgarten, Michael: 5180
Luth, Elizabeth: 2110, 7520, 7540
Lutsey, Pamela L.: 3510
Lutz, Gabriel: 4115, 4250
Lutz, Julie: 4300
Luu, Britney: 8900
Lv, Gang: 9000
Lv, Shao-bo: 7030
Ly, Timothy K.: 9210
Lydon, Elizabeth A.: 1315, 4415
Lynch, David H.: 4268, 7360, 7680
Lynch, Katie: 5005
Lynch, Scott M.: 3180
Lynn, Freda: 3445
Lyon, Debra: 9030
Lyons, Christopher: 3525
Lyons, Karen S.: 3095, 3535, 4235, 7470, 7540
Lytle, Ashley: 1080
Lyu, Jiahui: 7950
Lyu, Linyu: 7660

**M**

Ma, Carol Hok Ka: 2345, 7760
Ma, Chenjuan: 7810
Ma, Jing: 7270
Ma, Jinhui: 5070
Ma, Kris Pui Kwan: 9000
Ma, Qiyini: 7560
Ma, Xinlin: 9000
Ma, Yang: 9020
Ma, Yao: 7830
Ma, Yiming: 4050, 7600
Ma, Yingzhuo: 4535
Ma, Yu: 9010
Macali, Jennifer: 7480
Macaluso, Suzie: 7730
MacArthur, Michael R.: 9020
MacDonald, Catherine: 3300
MacDonald, Elizabeth: 4095
MacDonald, Stuart: 7420, 7470
Mace, Ryan A.: 4460
Macias Moreno, Isis S.: 4240
Maciejewski, Matthew: 7150
Mack, Laramie: 9000
Mack, Laurin J.: 4540

Mackenstadt, Darby: 2040, 3425
Mackenzie, Corey S.: 7590, 9430
Mackiewicz, Marissa: 9010
Mackin, R. Scott: 2240
MacKinnon, David P.: 9030
Mackowicz-Torres, Meredith: 9020
MacLellan, Cameron: 7660
MacLeod, Heather A.: 9000
MacNeil, Amanda M.: 9020
MacNeil, Andie: 3375
MacNeil, Maggie: 5085
MacNeil Vroomen, Janet: 7120, 7420, 9010
Macomber, Catherine: 2085, 4020
MacPherson, Andrew: 2165
MacPherson, Margaret: 9030
Madden, Kenneth: 3275, 5100, 7550, 7820, 9000
Maddock, Carol: 9420
Madhivanan, Purnima: 9010, 9020
Madiloggovit-Lower, Jirakate: 5225
Madrid, Vanessa: 7840
Madrigal, Caroline: 3365
Maeda, Harumi: 5065
Maeda, Nobuhiro: 3310
Magana, Kimberly: 9000
Magaziner, Jay: 3550
Mage, Susanna M.: 4420, 5120, 7700
Magee, Catherine: 7760
Maggiore, Sophia: 7380, 7580, 7900
Magid, Kate H.: 1160, 7160
Magnusson Hanson, Linda: 9030
Mah, Jasmine: 2170
Mahanna, Allexis: 5200
Maharani, Asri: 4455
Maher, Brion S.: 7870
Mahmood, Asos: 7960
Mahmood, Atiya: 1205
Mahmoud, Kafayat: 1185, 5055
Mahmoudi, Elham: 2300
Mahnken, Jonathan: 7990
Mahon, Elizabeth: 7970
Mahoney, Jeannette R.: 3480
Maier, Julie: 7720
Maina, Lucy: 2010
Maitra, Medha: 8900
Maitra, Medha Romee: 8900
Majid, Tabassum: 9000
Mak, Wingyun: 7890, 9010, 9030
Makarem, Nour: 1110
Makaroun, Lena K.: 2030
Makelarski, Jennifer: 7230
Makhmutov, Rustem: 1175
Maki, Pauline: 5225
Makineni, Rajesh: 2145
Malatyali, Ayse: 5100, 7620
Malcolm Barney, Javier: 7130
Maley, Kathryn: 7700
Malhotra, Rahul: 1055, 3435, 4025, 4175, 7090
Malik, Mishel: 7910
Malinsky, Daniel: 1110
Malle, Bertram F.: 5105
Mallender, William D.: 7080
Mallory-Hill, Shauna: 4515
Malm, Johannes: 7300
Malone, Susan K.: 1320
Mamo, Sara: 9010
Man, Kenneth: 2280
Man - van Ginkel, Janneke de: 3340
Manalel, Jasmine: 2055, 3565, 7810
Mancilla, Isabel: 7820, 8900
Manders, Imagine: 7390
Mandrell, Melissa: 5020
Manheim, Chelsea E.: 1160, 4408, 7720, 9310
Manini, Todd: 9000, 9580
Manis, Derek R.: 1210
Mankowski, Robert: 7870
Manly, Jennifer J.: 2225, 3405
Mann, Catherine M.: 7260
Mann, Christine: 7830
Mann, Jim: 3255, 3270
Mannheim, Ittay: 7590, 9210
Manning, Kevin J.: 2240
Manning, Lydia K.: 5170
Manning, Mark: 7960
Mannion, Greg: 7190
Manojlovich, Milisa: 7570
Manor, Brad: 1260, 2155, 3145, 7170
Mansion, Natalie: 3025
Manson, JoAnn E.: 7100
Mansoor, Marrium: 7100
Mansuy, Lauren: 4380
Manvelian, Atina: 9000
Mao, Hui-Fen: 7780
Mao, Weiyu: 1045, 2265
Mao, Ziling: 3475, 4260
Mar, Amanda B.: 7620
Maraganore, Demetrius: 9010
Maras, Jessica: 7040
Marchini, Leonardo: 3540
Marcille, Frankie Ann: 9010
Marcinek, David J.: 5210
Marcoe, Jennifer L.: 7620
Mardini, Mamoun: 9580
Marfeo, Elizabeth: 7850
Margolick, Joseph B.: 3390
Margot-Cattin, Isabel: 7690
Margrett, Jennifer: 7510
Maria, Lapid: 7330, 9000, 9010
Marier, Patrik: 7730
Marijnissen, Radboud: 9000
Marini, Christina M.: 1235
Marino, Francesca R.: 2150, 3335, 3390
Marino, Victoria R.: 3155
Markides, Kyriakos S.: 1165, 3050, 7600, 9560
Markle-Reid, Maureen: 5220, 7840
Markov, Yaroslav: 9010
Markwardt, Sheila: 9020
Marnfeldt, Kelly A.: 7790
Maroto-Rodriguez, Javier: 2320
Marottoli, Richard: 2085, 7540
Marquez, David X.: 1230, 4465
Marriott, Deanna J.: 9000
Marroig, Alejandra: 7660, 7830
Marron, Megan M.: 7630, 9330
Marrs, Sarah: 3425, 9030
Mars, Lori: 2220
Marshall, Gillian: 1230, 3450
Marsiske, Michael: 2180, 5070
Marston, Hannah R. R.: 9420
Martens, Nancy L.: 7510
Martin, Damali: 9030
Martin, Diane: 9010
Martin, Jessica G.: 7130
Martin, Lauren: 9410
Martin, Mike: 3115, 3130, 4225
Martin, Peter: 2170, 2380, 3555, 7070, 7460, 7930, 7950, 9020
Martinelli, Paolo: 7080
Martinez, Beanna: 7540
Martinez, Brandy S.: 1070
Martinez, Erica: 9030
Martinez, Julia Margaret: 2220
Martinez, Meghan: 3525
Martinez, Suzanna: 9020
Martinez Amezcua, Pablo: 2150
Martinez Tyson, Dinorah: 4010
Martinez-Amezcua, Pablo: 2150
Martin-Matthews, Anne: 3285
Martinos, Fadi: 1030
Martins-Klein, Bruna: 9020
Martire, Lynn M.: 4235, 9010
Martyr, Anthony: 4070
Marwaha, Rochisha Singh: 7480
Marx, Katherine A.: 2130, 7550
Marxen, Lucas J.: 2315
Mary Thoma, Jingxuan Wang: 2240
Marziliano, Allison: 4030
Masaki, Harue: 7620
Masaki, Kamal H.: 5060
Mason, Hannah: 7420
Mason, Justin: 7170
Masood, Zainab: 2325
Masri, Hadi: 7500
Massa, Franklin: 7660, 7830
Massar, Rachel: 7640
Massey, John R.: 9000
Massihzadegan, Setarreh: 9510
Massimo, Lauren: 4134, 4145, 7420, 7620
Masson-Trottier, Michèle: 7680
Mast, Benjamin T.: 3205, 7090, 7420, 9010
Mastel-Smith, Beth: 2080
Masternak, Michal: 4475, 7110, 7870, 9020
Masters, Julie L.: 7000, 7550
Masys, Anthony: 4010
Matatall, Elise K.: 3410
Mather, Harriet: 5160
Mather, Mara: 1215, 7510
Mathias, Rasika: 7110
Matos-Moreno, Amilcar: 3050, 4500
Matsumoto, Daisuke: 7330
Matsumoto, Yoshiko: 5065
Matsunaga, Hiroko: 7550, 7580, 7760
Matta-Singh, Tara D.: 3360, 4010
Mattek, Nora: 7620
Matteson, Tyler J.: 4480
Matthews, Fiona E.: 4070, 4455, 9110

Matthews, Karen: 4120
Matthews, Kathleen: 4095
Mattingly, Stephen: 7280
Matulis, Henrique: 4405
Matuszek, Gregory: 1290
Matz, Christina: 3095
Mau, Theresa: 3475, 5210
Mauldin, Rebecca L.: 7280, 9000
Maulod, Ad: 2230, 3435, 4175
Mausbach, Brent T.: 7610, 8900
Maxfield, Molly: 5080
Maxwell, Cathy A.: 3395, 9010, 9020, 9030
Maxwell, Colleen J.: 4280
Maxwell, Hillary: 3570
Maxwell-Crudup, Kasual: 4350
May, Jennifer T.: 9020
May, Patrick: 1225
Mayahara, Masako: 3345, 3445
Mayer, Jamie: 4406, 7480
Mayfield, Kellie: 9010, 9310
Mayhew, Alexandra: 5070, 5220
Maynard, Matthew C.: 2085, 7850
Mazer, Raquel: 7360
Maziarz, Lauren: 9000, 9020
Mbango, Catherine: 7310
McAlister, Jennifer: 7310
McAndrews, Carolyn: 2230
McAtee, Robin E.: 5190, 7390, 7540, 7680, 9030
McAvay, Gail J.: 1305, 2360, 7360
McCall, Stephen: 9020
McCarthy, Catherine: 9020
McCarthy, Ellen: 3565, 9010, 9540
McCarthy, Megan: 2075, 4010, 7300
McCarthy, Michael J.: 7400
Mccauley, Kathleen: 4545, 9010
McClaskey, Brady N.: 1360
McClellan, Angela: 1165, 1280
McClintock, Martha: 1230
McClure, Leslie A.: 4005
McConnell, Eleanor: 9110
McConnell, William R.: 1055
McCormack, Krista: 9410
McCoy Smith, Ellen: 4440
McCrae, Christina: 3420, 4490
McCray, Rachel: 7460
McCreedy, Ellen: 2035, 4190, 4290, 7020, 7540, 7640
McCulloch, Heather: 5040
McCullough, Megan B.: 2085
McCullough, Megan: 4290, 7540
McCurry, Susan M.: 5135
McDaniel, Kennedy: 1375, 9420
McDarby, Meghan: 7900
McDermid, Robert: 7120, 7160
McDermott, Katherine: 4460
McDonald, Claire: 9010
McDonald, Lynn: 1145
McDonald, Margaret V.: 2235
McDonald, Shelley: 1370
McDowell, Cynthia: 7470
McDuffie, Danielle L.: 4320
McFeeley, Brittany M.: 7510
McGee, Jocelyn S.: 3300, 9410
McGee-Lawrence, Meghan: 9030
McGibbon, Chris: 7830
McGilton, Katherine: 4515, 7650
McGinley, Jacqueline M.: 1005
McGinty, Patrick: 4268, 7680
McGovern, Justine: 3120
McGovney, Kevin: 4490
McGrail, Kim: 4280
McGrath, Ryan: 1360, 7990
McGraw, Meghan: 4315
McGuire, Cathleen: 9010
McGuire, Lisa: 1265, 2070, 3020, 3055, 5015, 5070, 9010
McGuire, Maura: 4170
McHugh, John: 1010, 9020
McHugh, Molly: 4545, 7980, 9010
McIntosh, Roger: 4340
McIntyre, Alissa C.: 7910
McIntyre, Deborah: 9020
McKay, Mary Jo: 3560
McKay, Michelle A.: 9000
McKay, Tara: 1180, 4000
McKee, Michael: 9000
McKelvey, Miechelle: 2080
Mckendrick, Karen: 3335
McKenzie, Colin: 4385
McKenzie, Glenise: 7440, 7470, 7540
McKibbin, Christine: 7610, 7840
McKiddy, Sarah: 7830
McKinley, Joseph: 7620
McLaughlin, Sara J.: 4435, 9010
McLean, Elisabeth: 7830
McLennan, Lauren: 1280
McLin, Maia: 7950
McMahon, Siobhan: 5100, 5205, 9410
McManus, David: 9030
McMillan, Barbara: 2020
McMillan, Corey: 7650
McMillan, Sincere S.: 7410
McMullen, Elyse: 4510
McMullen, Tara L.: 3175
McNally, James: 4175, 7380
McNamara, Kelsey C.: 9000, 9020
McNeil, Heather: 9020
McNeil, John: 7300
McNerney, Windy: 9010
McNicoll, Lynn: 9010
McPartlin, Adam: 7180
McPherson, Rachel: 3330
McPhillips, Miranda Varrasse: 3500
McQuarrie, Angelica: 5100
McReynolds, Melanie: 2205
Mcvey, Ava: 7080
Meadows, Jill: 9020
Meads, Morgan: 8900
Mecca, Marcia C.: 4060
Medders, Laura: 2300
Medina, Luis D.: 3050
Medina, Valerie: 7990
Medlin, Austin: 2070
Medrano, Theresa: 1380, 7370
Meeador, Rhoda: 2030
Meears, Rachel: 2145
Meehan, Amy: 9000, 9030
Meeks, Suzanne: 9010
Mehdiyar, Manijeh: 7360
Mehegan, Laura: 2035, 5040, 5175
Mehling, Wolf: 2035
Mehlsen, Mimi: 3195
Mehmood, Yasir: 7690
Mehrabi, Fereshteh: 5220
Mehta, Neil: 9030
Mehta, Priyanka S.: 3250, 4095
Mehta, Rhea: 7330
Mei, Lauren: 7510
Meier, Helen CS.: 2325, 7960, 9330
Meier, Naomi: 7390
Meier, Tabea: 4225
Meinertz, Naomi R.: 2075, 3445
Meinow, Bettina: 4190
Meiry, Lian: 4125
Meister, Melanie: 7330
Mejía-Arango, Silvia: 7540
Mejía-Guevara, Iván: 7970
Mejia, Shannon: 5195, 7030, 7590
Mekonnen, Teferi: 9020
Melady, Don: 5185
Melekis, Kelly A.: 3200, 3410
Melillo, Karen Devereaux: 1080, 5200
Melis, Rene JF.: 1370, 4485
Melissa, Macri: 9030
Melkonian, Matilda: 9010
Mell, Johnathan: 7440
Melo Resendes, Natasha: 9030
Meltzer, Gabriella: 3050
Mena, Emily: 2370
Mendelson, Gad: 7720
Mendez Campos, Barbara: 4420, 9010
Méndez-Chacón, Ericka: 9010
Mendhe, Dinesh: 4050, 7080
Mendoza, Lisandra: 2180, 5070
Mendoza, Nancy: 2005
Meneilly, Graydon: 7820
Meng, Hongdao: 3345, 5170, 7990, 9010
Meng, Lingsong: 7630
Menne, Dr. Heather: 9010
Menne, Heather L.: 7280, 9030
Mentor, Rhoodjinie: 8900
Meraz, Rebecca: 9410
Merdjanoff, Alexis: 5005
Merianos, Ashley L.: 7410
Merickel, Jennifer: 7510, 7620
Merkel, Emily: 3460
Merrilees, Jennifer: 1305, 4495
Merrill, Ray M.: 9010, 9020
Merss, Kristin: 9020
Merten, Natascha: 2240, 4120
Mertz, Laurel: 5125, 9020
Messier, Steve P.: 3505
Metzelthin, Silke: 3340, 7120, 7940
Meyer, Kayla: 4134, 7830
Meyer, Kylie: 2160, 5120, 9030
Meyer, Oanh L.: 3050
Meyers, David MW.: 4000
Meyers, Gabrielle: 7680
Meynadasy, Melissa J.: 7030
Mezuk, Briana: 3080, 3085, 4545, 5010, 5050, 9020

Mi, Hong: 4240, 4400, 7270, 7560, 7780
Mia, Mullen: 3425
Miao, Hongyu: 7460
Michael, Cherish: 4230, 7060, 7570, 7590
Michael, Yvonne L.: 1015
Mickus, Maureen A.: 5235
Middleton, Laura E.: 7300
Miech, Edward: 9000
Mielke, Michelle M.: 9200
Migliaccio, John N.: 4410
Migneault, Deborah: 1240, 4140
Migovich, Miroslava: 9030
Migrino, Lauren: 9420
Miguel-Cruz, Antonio: 1165, 5015, 9000, 9020
Mihailovic, Aleksandra: 2135
Mikels, Joseph A.: 1105
Mikkelsen, Mai B.: 3195
Mikkola, Tuija M.: 9010
Milani, Sadaf A.: 3295, 7600, 9560
Milberg, William: 9010
Milcik, Nicholas: 9010
Miles, Allyson: 3305
Milidonis, Mary: 4268, 7680
Milius, Rose: 7400
Miljkovic, Iva: 7830, 9020
Millar, Courtney: 9030
Millar, Roberto: 9000
Millenbah, Ashley: 1025, 7380
Miller, Brandi C.: 7100, 7870
Miller, Dante: 5010
Miller, Debra A.: 3015
Miller, Edward A.: 2245, 3165, 3350, 4390, 4435
Miller, Elaine L.: 7850
Miller, Henry: 1325
Miller, Julie: 1175, 3090, 3095, 5040
Miller, Katherine: 1175, 4365
Miller, Lindsay M.: 9500
Miller, Lyndsey M.: 3535, 4495
Miller, Melicent: 7620
Miller, Michael: 3505, 5230, 9010
Miller, Nancy D.: 9010
Miller, Rhonda: 2135
Miller, Richard: 7880
Miller, Ryan: 4080
Miller, Sara: 7390
Miller, Steven: 7140
Miller, Vivian J.: 9000, 9020
Miller-Winder, Amber: 7460, 7520
Mills, Sarah: 3345
Mills, Whitney L.: 3365, 4290, 7210
Milman, Sofiya: 1125
Milnamow, Mary: 7700, 9010
Mimbs, Hope: 4230, 7060, 7570, 7590
Min, Eun Jeong: 7160, 7800
Min, Joohong: 3130
Min, Jungwon: 1215
Mindlis, Irina: 3470
Minet, Hailey: 7050, 7960
Minster, Ryan: 5090
Minton, Alyssa R.: 1105
Mintz, Alan: 5050
Minyo, Morgan J.: 9020
Mion, Lorraine: 9010, 9030
Mira, Rolla Saud: 9030
Mirabella, Jack E.: 5220
Mirza, Raza: 7010, 9030
Mishra, Sidharth Prasad: 4475, 7100, 7870
Misonow, Julia: 2370
Mitchell, Anne B.: 4005
Mitchell, Calli O.: 9030
Mitchell, Colter: 2320, 2325
Mitchell, Emma: 7360
Mitchell, Jamie: 1270
Mitchell, Lori: 2170
Mitchell, Sarah J.: 9020
Mitchell, Susan: 2035, 7640
Mitha, Samrah N.: 7500
Mithani, Sara: 7910, 9000
Mitra, Shimontini: 5220
Mittal, Anuva: 3220
Mittelman, Mary: 2090, 9320
Mitzner, Tracy L.: 1120
Miyagawa, Shoko: 9030
Miyamae, Fumiko: 7280
Miyawaki, Christina E.: 1165, 1280, 2020, 3050, 4365
Mize, Mary Chase: 7070
Mizuochi, Masaaki: 7890
Mizutani Bacon, Suzette: 1310
Mlinac, Michelle E.: 9010
Mo, Cen: 4310
Moïse, Valerie: 1285
Moaddel, Ruin: 7880, 9020
Mochel, Amy: 4290
Mock, Marilyn: 1130, 7290
Mode, Nicolle: 9000
Modica, Cheryl: 9000
Moen, Maria: 3175
Moen, Phyllis: 2050, 3085
Moerk, Hannah R.: 9020
Moffit, Reagan E.: 3475
Moffitt, Terrie E.: 1305, 5080
Moghekar, Abhay R.: 2195, 4035
Mogle, Jacqueline: 1300, 1360, 2275, 3245, 4055, 4425, 7080, 7160, 9010, 9020
Mois, George: 1315, 4415
Molina, Anthony: 5210, 7370, 7820, 8900
Molinari, Alexa: 2130
Molinari, Victor A.: 5170, 7990
Molinar-Lopez, Veronica: 7830
Moll, Allison C.: 7510
Molli, Sam: 7070
Molnar, Lisa J.: 9030
Molony, Sheila L.: 9300
Molton, Ivan R.: 9580
Monane, Mark: 9550
Mondick, Lindsay: 1065
Mongelli, Jessica: 4030
Monin, Joan: 1305, 2055, 3360, 3535, 4010, 7420
Monroe, Stephanie: 1265, 2180
Monroe-Lord, Lillie: 7140, 7350
Monserud, Maria: 3105
Montayre, Jed: 4132, 7480
Montepare, Joann M.: 1330, 2080, 2250, 3150, 3325, 4160
Montero-Odasso, Manuel: 4120
Montesino Goicolea, Soamy: 7630
Montez, Shelby: 9030
Monti, Stefano: 5090, 5180, 7370
Montoro-Rodriguez, Julian: 7030, 7530
Montoya, Erika: 9570
Moo, Lauren R.: 2085, 3415, 7850, 9010, 9020
Moon, Chooza: 7990
Moon, Heehyul: 7530
Moon, Jukrin: 9030
Moone, Rajean: 4000, 5025, 7250, 9020
Mooney, Aimee: 7440
Moore, Ami: 7390
Moore, Ann Zenobia: 2195, 9020
Moore, Caroline: 5085
Moore, David J.: 3565, 4340
Moore, Jade: 7160
Moore, Jennifer: 5190, 7680
Moore, Justin: 3505
Moore, Logan: 7370
Moore, Miranda: 9030
Moore, Nadia: 9000
Moore, Sebastiana O.: 7410
Moored, Kyle D.: 1015, 2155, 3405, 4145, 9000
Moorman, Sara: 1000, 3095, 3105
Mor, Sharon Ost: 3275
Mor, Vincent: 2035, 4290, 7020
Moraras, Kate: 9000
Morato Stival, Marina: 7150
Morato Stival Lima, Marina: 7340
Moreines, Laura T.: 2235
Moreno-Agostino, Dario: 4510
Morgan, Ashley: 7180, 7340
Morgan, Brianna: 1380, 3290, 7840
Morgan, Davie: 9410
Morgan, Deborah: 9420
Morgan, Debra: 7650
Morgan, Jennifer: 3285
Morgan, Jennifer: 5190
Morgan, Jennifer: 7290, 7500
Morgan, Kerri: 7780
Morgan, Matthew R.: 5115
Morgan, Robert O.: 4290, 7540
Morgan-Daniel, Jane: 7170
Morgen, Keith: 3570, 7390
Morhardt, Darby J.: 7700, 9030
Mori, Koji: 9030
Moriello, Gabriele: 7950, 9000
Morikawa, Minami: 9000
Moritz, Robert: 7110, 7880
Morris, Brian J.: 5060
Morris, Emily P.: 1170, 9210
Morris, John: 7160
Morris, Nichole: 3460
Morris, Stephanie: 1390
Morrison, Cassandra: 7100
Morrow-Howell, Nancy: 2000, 3310, 4085, 4406, 7000, 9000
Morton, Cynthia: 3175
Morton, Cynthia: 7810
Morton, Patricia: 7960
Moser, Debra K.: 9000
Moshfeghi, Sonia: 5225
Moskow, Marian: 1335
Mosley, Thomas H.: 9200
Mosqueda, Laura: 1145, 2220, 4275
Moss, Karen O.: 2070, 3345, 4255, 5030

Mostafavi, Armin: 9010
Motel-Klingebiel, Andreas: 4450
Mouchaers, Ines: 7120
Moulton, Marjorie: 3255
Mousavi, Hossein: 9010
Mouton, Charles P.: 9010
Mouton, Peter: 7100
Mowat, Freya: 4120
Moxley, Jerad: 2050, 2060, 4268, 7680
Moy, Marilyn: 4095
Moya, Mario: 7130
Moye, Jennifer: 4095
Mozid, Nusrat E.: 9010
Mpofu, Elias: 7280, 7420, 9000
Mroczek, Daniel: 1255, 3230, 4300
Mroz, Emily L.: 3360, 4010, 9030
Mu, Aruhan: 3450, 7050
Mu, Christina X.: 3455, 3500, 7410, 7890
Muñoz, Elizabeth: 3080, 4185, 4425
Muchenje, Marvelous: 4340
Mudar, Raksha A.: 1260, 1315, 4415, 9020
Mudrazija, Stipica: 1185
Mudrazija, Stipica: 1265, 4325
Mudrazija, Stipica: 7950, 9020
Muhammad, T.: 3085, 7830
Mujahid, Nadia: 9010
Mujtaba, Muhammad: 7860
Mukherjee, Dahlia: 3135
Mukherjee, Namrata: 3410
Mukherjee, Nandini: 7370
Mulcahy, John: 3010, 4140
Mullachery, Balakrishnan: 7840
Mullavey, Judy: 5105
Mullen, Rebecca: 4205
Müller-Riemenschneider, Falk: 4385
Mullin, Donncha S.: 4134, 7620
Mullin, Patricia: 4150
Mullins, Megan: 1085
Mulvey, Michael: 3260
Mulvihill, Marie: 4525
Mumford, Elizabeth: 3075
Mumma, Kara T.: 1120, 7990
Mundy, Lindsey: 9000
Muniz-Terrera, Graciela: 1225, 3520, 4134, 4455, 5220, 7620, 7630, 7660, 7780, 7830
Munoz, Mariateresa H.: 4415
Munoz, Richard A.: 9420
Munoz Soto, Gianinna: 9000
Munroe, Leah A.: 9580
Munroe, Melanie: 1390
Munson, Taylor: 9010
Munsterman, Ellen: 7860, 7980
Muntefering, Chloe: 5000
Mur, Jure: 1225
Murabito, Joanne M.: 5090, 7640, 7830, 9000, 9030
Muraira, Joseph J.: 7590, 7900
Muralidhar, Kiranmayee: 9010, 9020
Muramatsu, Naoko: 4465
Murawski, Alaine: 7440, 7460, 7520
Murayama, Hiroshi: 7280, 7550, 7610, 7760
Murman, Daniel: 7510
Murphey, James M.: 5030
Murphy, Dallas J.: 7590, 9430
Murphy, James: 2100
Murphy, Rachel: 3275, 7550
Murphy, Rachel: 9520
Murphy, Sara: 4220, 4360
Murphy, Susan L.: 7680
Murphy, Terrence: 7120
Murray, Anna: 3215
Murray, Anne: 7300, 7370
Murray, Briana: 9020
Murray, Emily T.: 1015
Murray, James F.: 4210, 5105, 7620
Murthy, Venkatesh L.: 7630, 9300, 9330
Muruganand, Ashwath: 7230
Muruthi, James: 2010, 5130
Muruthi, Ndungu: 2010
Murzin, Kate: 4340
Musich, Madison: 3420, 7080
Musil, Carol: 2005, 3360, 7030, 7950
Muskin, Ryan: 9020
Mussallam, Noura: 7550
Musson, Nan: 7510
Mutambudzi, Miriam: 1180
Mutchie, Heather: 3550
Mutchler, Jan E.: 3350, 4435, 9000, 9510
Muyingo, Laura: 9000
Mwangi, Eunice Muthoni: 9110
Mwega, Elijah: 2010
Mwendwa, Denee: 9020
Mwinyogle, Aubrey: 7860
Myck, Michal: 1220
Myers, Alyson K.: 4268, 7680
Myers, Dennis R.: 7680, 9030
Myers, Jennifer: 7130
Myers, Marcella: 8900
Myrskyla, Mikko: 9030

**N**

Na, Annalisa: 4005, 9400
Na, Hunui: 1315
Na, Muzi: 1085
Naar, Sylvie: 3520
Nachtweih, Emily: 1150
Nadash, Pamela: 1175, 2245, 3165, 4295
Nadorff, Danielle K.: 1070, 2005, 7950
Naegele, Gerd: 4450
Naftolin, Frederick: 7100
Nagarajan, Niranjani: 2365, 9110
Nagarajan, Niranjani: 9110
Nagargoje, Varsha P.: 4385
Nagel, Corey: 2290, 2360, 7300, 7360, 7450, 7540, 7890, 7990
Nah, Suyoung: 4140
Naik, Aanand: 4060, 7630, 7840
Naik, Aishwarya: 4505
Nair, Malini: 9000
Nair, Neetu: 4490
Nair, Tripti: 9020
Nakagawa, Kazuma: 2380
Nakagawa, Takeshi: 4225
Nakamoto, Isuzu: 7610
Nakamura, Masahide: 9010
Nakamura, Misa: 9030
Nakashima, Taeko: 3210
Nakhai, Bita: 2270
Nalawade, Vinit: 3510
Nam, Seok In: 7200
Nam, Stephanie Jungah: 4265
Nanjaiah, , Shivamma Nanjaiah: 9010
Nanthakumar, Jarshini: 9330
Nardone, Michael: 9020
Narine, Ameesha: 9030
Narine, Donnette: 2375, 5110, 7040, 7380, 7600
Narusyte, Jurgita: 9030
Nasamran, Chanond: 7100
Nash, Veronika J.: 2280, 7410
Nashef-Hamuda, Reem: 9020
Nashiro, Kaoru: 1215
Näsman, Marina: 4330
Nasr, Lubna A.: 4240
Nasr, Samer: 1005
Nasser, Lucy: 9030
Nathalie, Veillette: 9000
Nathanielsz, Peter: 9000
Naumova, Elena N.: 1290
Navarro, Adria E.: 9000
Naveed, Haniya: 9000
Navia, R. Osvaldo: 9200
Navid, Sarah: 7600
Naylor, Mary D.: 4545, 7980, 9010, 9020
Nazario, Jaminette M.: 7950
Neal, Amy: 7180
Neal, Vanessa L.: 7510
Nearing, Kathryn A.: 2160
Nedelska, Zuzana: 7420
Neelamegam, Malinee: 2260
Neely, Stephen: 7300
Neff, Donna F.: 7810, 9010
Nehrkorn-Bailey, Abigail: 9010
Neiberg, Rebecca: 3505, 4355, 9010
Neiertz, Cynthia: 9210
Neil-Sztramko, Sarah E.: 5085
Neller, Sarah A.: 1150, 2075
Nelligan, Labrini I.: 2020
Nelson, Christi L.: 2310, 7450, 9010
Nelson, Ian Matt: 3190
Nelson, James F.: 4135, 4475
Nelson, Katie E.: 7770
Nelson, Matt M.: 3320, 9010
Nelson, Monica E.: 7420
Nelson, Niccole A.: 1070, 3245
Nelson, Sofie J.: 7260
Nemati, Donya: 5125, 7410
Nemec, Mary C.: 9320, 9500
Nemeth, Samuel R.: 4535
Nemmers, Natasha L.: 2075, 3360, 7070
Neppl, Tricia K.: 7550
Nerayo, Liwam: 9020
Neri, Mel: 9020
Neugebauer, Volker: 7830
Neugroschl, Judith: 2085
Neupert, Shevaun D.: 1255, 3195, 4180, 7550, 7590
Neville, Christine: 9000
Neville, Emily: 9000
Newbold, Bruce: 5085
Newman, Anne B.: 3475, 3500, 4260, 5085, 5090, 5210, 7630, 9300, 9330

Newman, David: 2180, 4132, 5225, 7480, 7580
Newman, John C.: 1380, 7110
Newman-Casey, Paula Anne: 7820
Newman-Norlund, Roger: 8900
Newman-Norlund, Sarah: 8900
Newmeyer, Natalie: 9410
Newsham, Tina M.: 2245, 3150, 3350
Newsom, Jason T.: 1305, 2360, 7300, 7450, 7890, 7990
Newton, Julia: 7160
Neylan, Thomas C.: 2280
Ng, Boon Peng: 2385, 7150, 7430, 9030
Ng, Crystal: 5035
Ng, Wai Chong: 7760
Ng, Yee To: 4145, 4230
Ngugi, Anthony: 9110
nguyen, Ann: 3005
Nguyen, Annie L.: 4275
Nguyen, Duy: 7230
Nguyen, Hoang T.: 4395
Nguyen, Khanh: 9530
Nguyen, Kim N.: 1165, 3050
Nguyen, Kimberly: 1290
Nguyen, Kristy B.: 4134, 7620
Nguyen, Uyen-Sa: 9530
Nhan, James: 9020
Nicholas, Marjorie L.: 1255
Nichols, Emma: 2365, 3160, 9110
Nichols, Jennifer A.: 4134, 7330
Nichols, Madeline J.: 4045, 7580
Nicholson, Meredith: 9530
Nicklas, Barb J.: 3475, 3505, 4350, 5210, 9010
Nicolay, Sarah: 5195, 9550
Nicosia, Francesca M.: 2035
Nie, Jing: 4240
Nie, Zuoting: 1260
Niedernhofer, Laura J.: 4200
Nielsen, Lisbeth: 3040
Nightingale, Ginah: 7500
Nikitin, Jana: 2105, 3100, 4055
Niknejad, Bahar: 9010
Nikzad-Terhune, Katherina A.: 2080, 3350
Nilsen, Charlotta: 3470, 7300
Nimrod, Galit: 7920
Ning, Hongting: 9300, 9320
Ningaiah, Nagalambika: 9010, 9020
Nishikitani, Mariko: 7940, 9000
Nix, Caitlyn A.: 2040
Niznik, Joshua: 7150, 7300
Nketsiah, Ebow T.: 7260
Nkimbeng, Manka E.: 1150, 3240, 3305
Nkwata, Allan K.: 7930, 9010
Noah, Naheem: 3075
Noblitt, Jessica: 4230, 7060, 7570
Noel, Megan: 7810
Noguchi, Kenneth S.: 5100
Noh, Hyunjin: 3005, 5125, 7260, 7620, 9000, 9020
Nolte, Julia: 1090, 7510
Nookaew, Intawat: 7880
Noone, John: 5210
Noppert, Grace A.: 3225, 9010
Noren Hooten, Nicole: 9000
Norgaard, Martin: 1120
Norris, Grace: 3270
Norris, Hayden S.: 7150
Northrup-Snyder, Kathlynn: 5205
Northwood, Melissa: 7840
Norton, Peter G.: 4408, 7720
Norton, Sally: 1335
Norton, Sara A.: 3520, 4320
Nothelle, Stephanie: 2185
Notthoff, Nanna: 5125
Noureddine, Sarah: 7110
Nouryan, Christian: 4268, 7260, 7680, 9010
Novak, Joshua R.: 4235
Novik, Nuelle: 4340
Nowacek, Nancy: 1080
Nowels, David: 4305
Nowels, Molly: 4305
Nowicki, Scott: 7940
Noyer, Emily: 9000
Ntailianis, Georgios: 7630
Núñez-Luna, Alejandra: 4395
Nunnery, Mary H.: 1160, 4408, 7720
Nunno-Evans, Michelle: 7950
Nwakanma-Akanno, Onyedikachi Chioma: 4130
Nwakasi, Candidus "Candi": 2010, 4130, 5055, 9000
Nweke, Chizobam: 2010
Nweke, Chizobam: 9000
Nyang'au, Getrude K.: 2055, 7950
Nygård, Mikael: 4330
Nyman, Fredrik: 5010
Nyquist, Linda: 9030
Nyqvist, Fredrica: 4330
Nyrup, Rune: 7190
Nyunt, Thedoe: 5060

**O**

Obbin, Samuel: 9020
Oberdier, Matt T.: 9020
Obregon, Alejandra Michaels: 3160
O'Brien, Catherine: 5125
O'Brien, Christopher T.: 7590
O'Brien, Erica: 7590
O'Brien, Jennifer L.: 7330, 9010
O'Brien, John: 7630
Ocampo-Chaparro, Jose M.: 7140, 7840
Ochiai, Gabriela S.: 9010, 9020
Ochoa Scussiatto, Henrique: 1230
O'Connell, Denise: 2020
O'Connell, Megan E.: 1200, 7650, 7660
O’Connor, Melissa: 9000, 9410
O'Connor, Melissa L.: 7300, 9000
O'Connor, Timothy D.: 7000, 7460
O'Conor, Rachel: 2355, 9030
Oczkowska, Monika: 1220
Odagami, Kishimori: 9030
Odden, Michelle C.: 1015, 4080, 4515, 5230, 7160
Odemuyiwa, Olumayowa A.: 9000
Odlum, Michelle: 1070, 1335, 3415, 7330
Odo, Obinna Casmir: 9010
Odom, J. Nicholas: 9010, 9020
ODonnell, Arden: 3410, 7670
O'Donnell, Amy J.: 4540, 9010
O'Donovan, Aoife S.: 2280
O'Driscoll, Leo: 5150
Odukoya, Tolu: 5025
Odzakovic, Elzana: 7300
Ofori, Edward: 7100, 9030
Ofotokun, Ighovwerha: 3565
Ogawa, Elisa F.: 9010
Ogidigo, Oloaigbe (Joyce) O.: 2125
Ogle, Destiny: 1050, 1255
Ogletree, Aaron M.: 3210
Ogliore, Anthony M.: 9020
O'Grady, Thomas: 3365, 7260
Ogungbe, Oluwabunmi: 7960
Ogunjesa, Babatope: 7610
Oh, Esther S.: 2185
Oh, Eunna: 7660
Oh, Gahee: 3565
Oh, Hans: 9020
Oh, Hayoung: 4145
Oh, In-Hwan: 7380
Oh, Jeewon: 9020
Oh, Kyeung Mi: 7700
Oh, Patricia: 4150
Oh, Seokmin: 7990
Oh, Seung-Eun: 5055
Oh, Suhee: 7810
Oh, Yun taek: 3085, 9000
O'Hanlon, Julia: 9020
Ohuabunwa, Ugochi: 4525
Ojo, Eunice Oladepe O.: 7080, 9030
Okada, Jamil: 9030
Okamura, Tsuyoshi: 7280
Okello, Samson: 9000
Okem akwiwu, Chika: 4130
Okereke, Olivia I.: 4460
Okeyo, Hilda: 9000
Okhravi, Hamid: 9010
Okine, Joana: 4275
Okoli, Chizimuzo: 2280
Okolie, Jonathan: 9010
Okoye, Safiyyah M.: 5130, 5160
Okpalefe, Okiemute: 2125
Okrah, Prince A.: 9030
Oktapodas Feiler, Marina: 7150
Okuyan, Canan: 4120
Okwaro, Lynnete: 7290
Oladeinde, Yewande: 3210
Oladejo, Dianne: 2355, 9030
Olanrewaju, Sherif: 9000
Olausson, Jill: 7850
Olazo-Cárdenas, Kamyla M.: 9010
O'Leary, Bryant J.: 5075, 7910
Olivari, Benjamin S.: 2070, 5015, 5070, 7700
Oliveira, Eduardo: 7290
Oliveira, Iara B.: 2375, 3365
Oliver, Joanna T.: 4265
Oliver, Michael D.: 7100

Oliver, Sloan: 7620
Olivieri-Mui, Brianne: 3565, 9000, 9010, 9430, 9540
Olokunlade, Temitope: 7430
Oloruntoba, Oluyomi: 7410
Olson, John D.: 7370
Olson, KayLoni: 7630
Oltmanns, Thomas F.: 3520, 4320, 9020
Olvera, Charles: 7460, 7520
O'Malley, Kelly A.: 1365
O'Malley, Terry: 3175
Omeje, Kingsley U.: 2125
Omura, John D.: 2070, 5015, 5070
O'Neil, Kaitlyn: 7700
Ong, Anthony D.: 1255
Ong, Lydia Q.: 4320
Ong, Michael: 7900
Oni, Julius: 9010
Ono, Keiko: 9010
Onsando, Wambui: 9010
Oosterhouse, Kimberly: 9000
Openo, Kyle P.: 4130
Opoku-Agyemang, Christfelly: 7330
Oppong, Richard F.: 5085
Oranchuk, Dustin J.: 9020
Orange, Joseph B.: 3270
Oravecz, Zita: 5165, 7510
Orewa, Gregory N.: 2350, 3365, 4190
Orkaby, Ariela: 4310
Orkaby, Ariela: 9410
Orlovsky, Irina: 9020
Ornstein, Katherine: 1375, 3415, 4165, 4365, 5160, 7650, 9030
O'Rourke, Tarryn: 7750
Orr, Miranda E.: 2335
Orsega-Smith, Elizabeth: 7460, 9020
Orsulic-Jeras, Silvia: 7060, 7460, 9550
Ortega, Alexandra: 9010
Ortega, Julia L.: 9550
Ortega, Marcus: 7100
Ortega Hernández, María: 1055
Orth, Jessica: 5015
Ortiz, Hector: 2025
Ortiz, Juliana: 9010
Ortiz Sarmiento, Erika: 4268, 7680
Ortmeyer, Heidi: 7840
Ortola, Rosario: 2320
Oruru, Daisha: 7460
Orwig, Denise: 3550, 5205, 7130
Orwoll, Eric: 4260, 5085
Ory, Marcia: 9030
Osakwe, Zainab Toteh: 1335, 3415
O'Súilleabháin, Páraic S.: 7990
Osei, Elizabeth: 7790
Osevala, Nicole J.: 5145
Osika, Jocie: 4300
Osokpo, Onome: 4545, 9010, 9020
Osso, Francesco S.: 2075, 7300
Østbye, Truls: 2265, 3180
Osterbur, Elaina: 9030
Ostrem, Nicholas: 7440
O'Sullivan, Julie L.: 9400
O'Sullivan, Kelly: 1365
O'Sullivan, Roger: 2135, 4160
Osuna, Margarita Maria M.: 3465
Oswald, Austin: 1250, 2310, 5150, 9440
Oswald, Catherine L.: 9030
Oswald, Frank: 2340
Otero, Marcela: 4300
Otis, Laurie: 9400, 9440
O'Toole, Mia S.: 3195
Ou, Alison X.T.: 7520
Ouchi, Kei: 3300
Oude Voshaar, Richard C.: 9000
Ouslander, Joseph: 7670
Oviawe, Elizabeth: 7500
Owen, Donna C.: 7160
Owino, Jonix: 3380
Owsiany, Montgomery T.: 7030, 7570
Oya, Akari: 5035
Oylu, Gulin: 4450
Ozawa, Tabasa: 3160

**P**

P. de Paula Couto, M. Clara: 3100
Paccione, Jane A.: 2160
Pace, Sarah: 3025
Pachana, Nancy A.: 7550
Pachur, Thorsten: 1090
Padakandla, Deepthi: 4190
Padden, Eleni: 9000
Padgett, Deborah: 5170
Padron, Dilianna: 2180, 5070
Pagan, Mary Theresa: 5190, 7000
Pagan, Michael: 2315
Page, Riley: 9410
Pai, Manacy: 3085
Pajewski, Nicholas M.: 4355
Pajonk, Frank: 8900
Pajuelo-Vasquez, Renzo: 9010, 9030
Pakhomov, Serguei: 7300
Pal, Subhamoy: 9020
Palavicini, Juan: 1325, 9000
Palella, Frank J.: 3390
Palen, Ted: 1240
Palermo, Francisco: 3080
Palgi, Yuval: 3275, 4180, 9420
Palimaru, Alina: 9010
Palleschi, Mary-Kate: 9010
Pallis, Maryssa: 1175
Palmer, Allyson K.: 1325
Palmer, Barton W.: 7610
Palmieri, Jim: 5175
Palms, Jordan D.: 1170, 4325
Palovics, Robert: 4065
Palta, Priya: 5085, 7340, 9200
Pan, Xi: 7830, 7960
Panday, Somi: 4132, 7480
Pandya, Naushira: 2297, 7000, 7500
Panellas, Isis O.: 9030
Pangle, Amanda: 4130, 9000, 9020
Pani, Jasmine: 9020
Panta, Pradip: 9030
Papaioannou, Alexandra: 4268, 5100, 7220, 7680, 7710, 9020
Pappa, Sara: 9010
Pappadis, Monique R.: 9010
Parada Jr., Humberto: 3390
Parast, Mana M.: 8900
Parchman, Michael: 7810
Parekh, Rupal: 7280, 9020
Parini, Angelo: 2320
Paris, Steve: 9020
Park, Chan Mi: 9540
Park, Chang: 4025
Park, Christina: 7830
Park, Crystal L.: 1390
Park, Elyse R.: 9030
Park, Esther: 9000
Park, Geunhye: 2055
Park, Grace: 7120
Park, Hayoung: 7040, 7080, 7610
Park, Hyejin: 9010
Park, Hyeran: 7400
Park, Hyeyoung K.: 9030
Park, Hyunju: 7300
Park, Isabella: 9010
Park, Ji Yeong: 7160
Park, Jihyun: 9020
Park, Joon-Hyuk: 2385, 5075, 7080, 7150, 7430, 7680, 7820, 9010, 9020, 9030
Park, Junmin: 9030
Park, JuYoung: 2125
Park, Juyoung: 7420
Park, JuYoung: 7670
Park, Juyoung: 9000
Park, JuYoung: 9010
Park, Juyoung: 9020
Park, Kyoung Shin: 9030
Park, Lindsay (Na Yeon): 7180
Park, Min Kyung: 7320
Park, Min-Kyoung: 9010
Park, Moonkyoung: 7660
Park, Nan S.: 7510
Park, Seho: 1040
Park, SeongHu: 7850
Park, Sojung: 4266, 7690, 9010, 9030
Park, Soobin: 4266, 7690, 9010, 9030
Park, Stella: 4095
Park, Suah: 7910, 9010
Park, Sujeong: 7330
Park, Sumin: 7780
Park, Sung S.: 3065
Park, Sung Kyun: 9010
Park, Sungjae: 2145
Park, Taeyoung: 7360
Park, Van Ta: 1165
Park, Yoonsei: 7780
Parker, Andrew M.: 1090
Parker, Daniel C.: 1370, 9000
Parker, Elizabeth A.: 7340, 7350, 7840, 9010
Parker, Kelly RB.: 1360, 7990
Parker, Lauren: 1265, 2130, 3240, 7550
Parker, Myra: 9100
Parker, Patricia A.: 7900
Parker, Robert: 2035
Parker Oliver, Debra: 4415, 9000
Parkinson, Luisa: 9020
Parkman, Suzanne E.: 7850
Parmelee, Patricia A.: 7460
Parris, James J.: 2145
Parrish, Elise: 1175
Parrish, Monique: 7700
Parsons, Nathan: 9010
Parsons, Pamela L.: 3025
Pascoal, Tharick A.: 9500
Pascoe, Tori: 2300
Pascual-Leone, Alvaro: 2155, 9030
Pascuzzi, Emma N.: 3385
Passegue, Emmanuelle: 3070
Passos, Lígia: 2215, 7300
Patano, Arienne: 9000
Patapoff, Molly A.: 7610
Patel, Charmi S.: 9010, 9030

Patel, Himalaya: 7080
Patel, Juhi: 3520
Patel, Kadam: 4130
Patel, Karishma: 9330
Patel, Khushbu S.: 7830, 7950
Patel, Manali: 3525
Patel, Mehul: 9020
Patel, Priya: 4030
Patel, Roshni: 2070, 5070, 9010
Patel, Snehal: 3345
Pathak, Deeya: 9010
Pathak, Parth: 7750
Patille, Rachelle: 1205
Patricia, Boyle: 1230
Patskanick, Taylor: 7090, 7910, 9010
Patt, Stephanie: 7010
Patterson, Logan: 3250
Patterson, Sarah: 1340, 4055
Patti, Gary J.: 5180, 9020
Patton, Rikki: 4406, 7500
Patyal, Pankaj: 7100, 7370, 7880
Paudel, Anju: 1300, 3020
Paul, Constanca: 3130, 4406, 7000, 7300
Paul, Deepak: 4495
Paul, Eli: 9000
Paul, Kimberly: 7370
Paulsen, Adam J.: 2240
Paulsen, Michelle: 9030
Paulson, Sally: 2015, 7810
Pauly, Mark: 4545
Pauly, Theresa: 3275, 4235, 4480, 9000
Paun, Olimpia: 3445, 7420, 9030
Pavlovic, Noelle: 5175
Pavon, Juliessa M.: 7150
Pawlicki, Matthew: 7130
Payen, Ameante: 1320
Payne, Laura: 7610, 7940
Pearman, Ann: 7330, 7510, 7550
Pearson, Thomas: 9000
Pebley, Anne: 3065
Pechin, Sue: 9020
Peckenpaugh, Jackson W.: 9410
Peckham, Allie: 5080, 7780
Pedersen, Dorthe A.: 4260
Pedersen, Megan: 9000
Peek, Monica: 9010
Peeples, Amanda D.: 4095, 8900
Peerman, Carey: 5170
Peet, J. Zak: 7080
Peeters, Geeske: 1370
Pei, Sen: 1110
Pei, Yaolin: 2090, 2265, 3540, 9010, 9020, 9320
Peiro-Milan, Inma: 4330
Peleg, Shira: 4480
Pelkmans, Jordan: 3535
Pelletier, Chelsea: 7300
Pena, Marie: 8900
Pena, Triana: 9000
Penconek, Tatiana: 4408, 7720
Pendergrast, Claire: 7280
Pendrey, Anna: 7130, 9000
Penfold, Robert B.: 5135
Peng, Changmin: 3165, 7510
Peng, Huamao: 5115, 7000, 7060, 7950
Peng, Li-Min: 4155
Peng, Siyun: 4500, 9020, 9300
Peng, Weisi: 9020, 9520, 9540
Peng, Wenting: 4310
Penichet, Eric N.: 7370
Penney, Lauren S.: 1005, 7130, 7180
Penning, Nehle: 4450
Pentecost, Claire: 4070
Perales-Puchalt, Jaime: 7330
Peralta, Eriana: 9000
Peralta, Sawyer: 7110
Perek-Białas, Jolanta: 4450
Perepezko, Kate: 5000, 7800
Perera, Rohini: 9010
Perez, Eliany: 9010
Perez, Gilberto: 4060
Perez, Hector: 5015
Perez, Jacqueline: 4400
Perez, Sasha: 1335, 3415, 4266, 7190, 7850
Perez Montes, Viviana: 4200
Periassamy, Mithran: 7060
Perion, Jennifer: 7540
Perissinotto, Carla: 4205, 7610
Periyakoil, Divya: 9560
Perkhounkova, Yelena: 2160, 3335, 5015, 7300
Perkins, Molly: 1010, 1375, 3435, 3565, 9030
Perkins, Rebekah: 4406, 7000, 9000
Perkins, Susan: 9400, 9440
Perlman, Saritte: 4255
Perls, Thomas T.: 1040, 5090, 5180, 7370
Perone, Angela K.: 1100, 3200, 7280
Perre, Taylor: 3365, 7260
Perreira, Krista M.: 3320
Perry, Brea: 4500, 9020, 9300
Perry, Kaley: 9000, 9020
Perry, Morgan: 9020
Perry, Tam E.: 1270, 4150, 7690
Perryman, Collin: 9000
Persad, Alyssa: 9000
Pervratil, Michael: 1315, 9000
Perweiler, Elyse: 1130, 7290
Perzynski, Adam T.: 1170
Perzynski, Joseph: 3015, 5000
Peskoe, Sarah: 4485
Pestine-Stevens, Althea R.: 1330, 2315
Peszka, Jennifer: 7550
Petereit, Daniel: 7600
Peters, Elizabeth: 1185, 7950
Peters, Ruth: 9110
Peterson, Colleen M.: 3570, 7080
Peterson, Daniel S.: 9030
Peterson, Jennifer: 9010
Peterson, Lindsay J.: 2295, 4435, 5170
Peterson, Matthew: 3150
Peterson, Neil E.: 7980
Peterson, Tina L.: 7980
Petri, Florence: 3015
Petrovic, Milenko T.: 9000
Petrovskaya, Olga: 3570
Petrovsky, Darina V.: 2110, 4005, 9000
Pettee Gabriel, Kelley: 7650, 9010
Pettigrew, Corinne: 3450, 7970
Pettis, Jennifer: 9000
Pflieger, Lance: 7110
Pfund, Gabrielle N.: 3245, 3520
Pham, Julie Q.: 9000
Phan, Dennis: 9030
Phan, Phillip: 5175
Phang, Howard J.: 7370
Phatak, Vaishali: 7510
Phelan, Elizabeth A.: 1280, 2385
Phifer, Lisa: 9020
Philips, Joanna: 9000
Phillips, Bethan: 4470
Phillips, Christine: 7080
Phillips, Karon: 1330
Phillips, Michael M.: 9000
Phillips, Mirelle: 7680
Phillips, Natalie A.: 7650
Phillips, Nicole: 3450
Phillips, Stuart: 5085, 5100
Phipps, Denham: 4110
Phongtankuel, Veerawat: 7360
Phyo, Aung Zaw Zaw: 7370
Piazza, Jennifer: 3230
Picard, Martin: 1110
Picchiello, Matthew C.: 4210, 4406, 7000
Piccinin, Andrea M.: 3520
Piccitto, Giorgio: 9030
Pichardo, Abel: 8900
Pichora-Fuller, Kathleen: 4110
Picken, Lauren: 9000
Pickering, Carolyn E.: 1075, 5140
Pickett, Andrew C.: 7720, 9020
Piechota, Amanda: 3360, 4010
Piedra, Lissette M.: 1230, 4205
Pieper, Carl F.: 4485, 9010
Pierce, Aimee: 4100, 7620
Pierce, Matthew J.: 7550
Pierre-Louis, Saamantha: 5055
Pierson, Katie R.: 3485
Pieruccini-Faria, Frederico: 4120
Pietruszka, Brittany L.: 2145
Pike, James R.: 3510
Pillemer, Karl: 1145, 2030, 2050, 2060, 2105, 2295, 3375
Pilonieta, Giovanna: 9020
Pimentel, Camilla B.: 3365
Pinheiro, Cristina: 9020
Pinho, Hianca: 3540
Pinto, Jayant M.: 1230, 1350
Pirolli, Peter: 4268, 7680
Pituch, Keenan A.: 5075
Pizzi, Laura T.: 2130
Plasencia, Rosa: 7810
Plassman, Brenda L.: 3540
Platts, Loretta G.: 3370
Player, Nathanael: 1155
Pleasants, Debbie: 4355
Pless Kaiser, Anica: 1160
Pletcher, Scott: 2335
Plotkin, Elana: 7500
Plotzke, Michael R.: 3230, 4195
Ploubidis, George: 4510
Plow, Matthew: 9030
Plummer, Narcissa: 9010
Plys, Evan: 3525, 4525, 7680
Podder, Avijit: 5180
Poe, Abigail: 3545, 4145, 7090

Pohl, Janet S.: 5075
Pohnert, Anne M.: 4525, 7590
Polenick, Courtney A.: 4235, 4425, 5035, 7390
Polhemus, Ashley: 3550
Poliakov, Oleksandr A.: 7820
Pollack, Craig E.: 5130
Pollak, Chava: 9020
Pollard, Chris: 5220
Pomeroy, Mary Louise: 5160, 9030
Pompeu, Jose Eduardo: 9010, 9020
Ponce Gonzalez, Kimberly: 1150
Pond, Brittney: 5150, 9020
Ponto, Kevin: 9030
Poon, Cecilia: 3250
Poonsap, Sukanya: 7860
Popat, Shena: 1065
Pope, Caitlin: 4340, 7060
Pope, Natalie E.: 1330, 4150
Pope, Natalie D.: 5020, 7060, 9000
Popejoy, Lori L.: 1310, 7140
Popelsky, Braden: 7570
Poplawski, Antonina: 7760
Porat-Dahlerbruch, Joshua: 1175, 4255
Port, Cynthia: 3205
Portacolone, Elena: 9020, 9550
Porter, Kristie A.: 2375, 3365
Porter, Michelle M.: 4515, 7590, 9430
Porter Starr, Kathryn N.: 1370, 4350, 9000
Portz, Jennifer D.: 1240
Poss, Jeff: 2170
Possin, Katherine L.: 9320
Potok, O Alison: 9500
Pottayil, Faheem: 9500
Potts, Daniel C.: 1365
Powell, Danielle: 2185
Powell-Versteeg, Amy N.: 4406, 7000
Power, Shannon R.: 9000, 9320
Powers, Becky: 7180
Powers, James: 3120
Powers, John David: 1240
Powers, Sara: 7060, 7460, 9550
Pradhan, Rohit: 2350
Prager Geller, Tal: 7720
Prajapati, Santosh Kumar: 4475, 7100
Prakash, Sreelekha: 7290
Praparpak, Werapattra: 7860
Prasad, Anyah: 4390
Prasad, Jitender: 7320
Prata, Ndola: 9560
Pratley, Richard: 7620
Prats, Madison: 9010
Pratt, Borianna: 3065
Preble, Edward: 3075
Precupetu, Iuliana: 1220
Preddie, Alaina K.: 7130, 7180
Preis, Sarah R.: 4310
Premovich, Alyssa M.: 7910
Prendergast-Boughrum, Alice: 7690
Prescott, Amy: 9430
Pretzer-Aboff, Ingrid A.: 9330
Price, Caitlin N.: 7150
Price, Elvin: 9010
Price, Nathan D.: 5180
Primomo, Janet: 7910
Primossi, Valentina: 3260
Prince, Devlin: 7770
Prioli, Katherine: 2130
Probst, Karen: 4408, 7230
Procter-Gray, Elizabeth: 1350, 2385, 3155
Proffitt, Rachel: 1310
Prosperi, Mattia: 7860
Proulx, Christine M.: 3080, 4235, 4370
Provencher, Véronique: 9000
Province, Michael A.: 9010, 9020
Pruchnicki, Alec D.: 7140
Prybutok, Gayle: 7280
Przkora, Rene: 1030
Pubien, Sheena: 9420
Puche, Gabriel Prieto: 4415
Pudelek, Kelly M.: 1230
Pudur, Rekha: 7280
Puga, Frank: 1075, 3545, 4145, 7090, 7680, 9010, 9020
Pugh, Mary Jo: 9010, 9030
Puhan, Milo A.: 3550
Pulido, Joanne M.: 2125
Pullen, Carly E.: 1365, 7760, 8900
Pumilio, Victoria L.: 7500
Punke, Elizabeth: 7610, 7840
Puterman, Eli: 3520
Putnam, Michelle M.: 7780
Putz, Leann: 9520
Pynoos, Jon: 4270

### Q

Qazi, Amna: 7700
Qazi, Saadia: 4310
Qi, Bingbing: 9010
Qi, Liangshu: 9000
Qi, Meiyuzhen: 4120
Qi, Mingyu: 1115
Qi, Xiang: 1045, 1320, 2090, 2265, 3540, 9010, 9020
Qian, Yong: 5085
Qian, Yuting: 1165
Qiao, Yujia (Susanna): 3475, 5210
Qin, Vicky Mengqi: 7090
Qin, Weidi: 3005
Qin, Yue: 3105
Qiu, Tian: 9030
Qiu, Xiao: 9030
Qreshi, Mariam: 7500
Qu, Chaoran: 9020, 9520, 9540
Qu, Shan: 1175, 9010
Qu, Xiaomin: 1045
Qu, Yang: 7920
Quach, Emma: 3415, 7770
Quach, Ha-Linh: 3435
Quach, Lien T.: 9530
Qualls, Sara H.: 7090
Quashie, Nekehia Tamara: 2115, 3050
Quatsoe, Alissondra: 9010
Quellhorst, Jennifer A.: 3110
Quiñones, Ana R.: 2360, 7300, 7360, 7450, 7540, 7890, 7990, 9020
Quillen, Ellen E.: 7370
Quinlan, Claire: 9000
Quinn, Catherine: 4070
Quinn, Deirdre A.: 7580
Quinn, Joseph F.: 3370
Quinn, Marielle: 2055

### R

R. Goodin, Burel: 7630
Rababa, Mohammad: 7610
Rabinovich, Alexander: 9020
Rabinowitz, Jill A.: 4265, 4490, 7870
Rachmuth, Lisa: 3090
Radpour, Siavash: 3370
Raetz, Jaqueline G.: 7830
Rafferty, Jane: 9020
Ragab, Ahmed A.Y.: 7640, 7830, 9000
Raghavachari, Nalini: 5090, 5180
Rahemi, Zahra: 4005, 9000
Rahman, Farzana: 9010
Raimundo, Nuno: 5060
Raina, Ketki: 7300
Raina, Kirandeep: 7440
Raina, Parminder: 5070, 7660
Rainville, Chuck: 2035
Raj, Hayley: 5000
Raj, Minakshi: 4415
Rajagopal, Shilpa: 3295
Rajan, Ashna: 9010
Raji, Mukaila: 3295, 9010
Rajman, Luis: 9020
Raju, Anish: 5180
Rajupet, Sritha: 9020
Rakel, Barbara: 9020
Ralston, James D.: 5135
Ram, Nilam: 9010
Ramachandran, Meena: 7620
Raman, Vaishnavi: 9410
Raman-Wilms, Lalitha: 9520
Ramírez-García, Daniel: 4395
Ramel, Alfons: 5230, 7350
Ramirez, Ana V.: 7440
Ramirez, Maggie: 5135
Ramirez, Mildred: 1145
Ramirez, Saul: 4420
Ramirez, Sofia: 9010
Ramirez-Garcia, Daniel: 3160
Ramirez-Zohfeld, Vanessa: 7440, 7460, 7520
Ramisetty-Mikler, Suhasini: 9010
Ramos, Diêgo Araújo de Moraes: 9020
Ramos, Mary Dioise: 7850, 9000
Ramos, Sofhia V.: 5210
Ramos de Lima, Luciano: 7150, 7340
Ramrattan, Anita: 7150
Ramsey, Jennifer: 7030
Ramulu, Pradeep: 2135
Randa, Cari: 2340
Randall, Kevin: 7050, 7960
Randazzo, Aliza: 3505
Rane, Manas: 4310
Rane Levendovszky, Swati: 9000, 9010, 9020
Rank, Jenny: 4315
Rao, Ruchira: 4415
Rao, Smitha: 3045
Raper, James L.: 4340
Rapoport, Andy: 9530
Rapowitz, Jennifer: 4415
Rapozo, Crystalinda: 7130, 7840
Rapp, Stephen R.: 4134, 4355, 7830
Rappaport, Noa: 5180, 7110, 7880
Rasekaba, Tshepo: 2260, 9010

Raszewski, Rebecca: 4025
Rataj, Alison C.: 5020
Ratangee, Brina A.: 4510
Ratcliff, Amy C.: 9020
Rath, Shavonnye: 8900
Rathbun, Alan: 4260, 7330
Ratliff, Kim: 9010, 9540
Ratnayake, Aneeka: 9000, 9010
Ratner, Edward R.: 7280, 7420, 7750
Raue, Patrick: 3560
Rauer, Amy: 1235, 3060, 4370
Raulston, Journie: 4100
Raval, Aasha: 7120
Raval, Minjaal: 7760
Ravel, Jacques: 4080
Ravindran, Malcolm S.: 2230
Rawoof, Anam: 8900
Rayha, Kara: 7420
Raymo, James: 7890
Razjouyan, Javad: 7180, 7630
Reader, Jonathan M.: 9020
Reading Turchioe, Meghan: 2070
Rebala, Sri: 4170
Rebok, George W.: 3335, 3510
Rebola, Claudia B.: 5105
Reboussin, David: 4134, 7830
Reckrey, Jennifer: 3335, 3415, 4165, 5160, 7650
Reczek, Rin: 4520
Redd, Tara: 3300
Redding-Raines, Audrey: 7990
Reddy, Ann: 7640
Reddy, Sweatha: 7420
Redline, Susan: 4355
Redmond, Leslie: 9010
Redwine, Pamela: 7590
Reed, Alexandra M.: 9030
Reed, David: 1210
Reed, Lai: 3235
Reed, Mickaela R.: 7920
Reed, Nicholas: 1295, 2020, 2135, 2150, 2185, 2240, 2365, 4110, 4265, 9010
Reed, Peter: 4075
Reeder, Blaine: 1310
Reel, Candice D.: 1365, 9010
Reese-Melancon, Celinda: 7510
Reeves, Alexis: 4080
Regan, Claire: 9010
Regan, Kayla: 7300
Regan, Michael A.: 3570
Regier, Natalie G.: 1075, 7400, 9020
Reich, Richie: 3420
Reichert, Monika: 4450
Reid, Colin: 7700
Reid, Kieran F.: 3480, 9000
Reid, M. Cary: 4268, 7360, 7680, 7900
Reider, Lisa: 3550
Reiff, Jenni Seale: 1035, 5160
Reilly, Elizabeth: 9000, 9010
Reinhard, Susan C.: 2200, 2395, 4295
Reinhardt, Joann p: 9030
Reiter, David A.: 3495
Reitlo, Line S.: 9020
Rejeski, W J.: 3505
Relerford, Raven: 7460, 7520
Remiker, Mark: 7400
Remillard, Elena T.: 1120
Ren, Dianxu: 4210, 9020
Ren, Haopu: 3570
Ren, Haoyu: 3550, 9000
Ren, Jinnan: 9010
Rendell, Peter: 7510
Renn, Brenna N.: 4134, 7330
Renn, Cynthia: 7170
Rerick, Peter: 7830
Research Group, PREVENT-AD.: 7100
Research Team, DREAM.: 7300
Resnick, Barbara: 1300, 2210, 2305, 3330, 3340, 4040, 7170, 7300, 9020
Resnick, Susan M.: 2155, 2195, 3390, 3510, 4035
Ressler, Deidra A.: 5090
Revenson, Tracey A.: 3470
Reyes, Andrew-Thomas: 4040
Reyes, Mariana: 7700
Reyes-Ortiz, Carlos A.: 7140, 7840
Reyes-Vega, Andrea: 9000
Reynolds, Addam S.: 4195, 4210
Reynolds, Caitlin M.: 1080
Reynolds, Chandra A.: 3080, 4185, 9000, 9010
Reynolds, Charles: 5175
Reynolds, Courtney: 2060, 2220, 4215
Reynolds, Lindsay M.: 5060
Rhee, Yeong: 7990
Rhodes, Ann: 3285, 7620, 9030
Rhodes, Shane: 5120
Riang'a, Roselyter M.: 9110
Ribeiro, Oscar: 2215, 3130, 3555, 4406, 7000, 7060, 7200
Rich, Katherine: 2055
Rich, Stacey: 2260, 9010
Richard, Shannon L.: 4134, 7330
Richards, Bradley: 1240
Richards, Emily: 1015, 9000
Richards, Kathy: 4290, 7540
Richards, Nicole E.: 7070
Richards-McCullough, Kerry: 9010
Richardson, Connor: 4455
Richardson, Joann: 9000
Richardson, Julie A.: 7170, 7180, 7340
Richardson, Katy: 7610, 7840
Richmond, Therese: 9030, 9540
Richter, David: 9010
Riffin, Catherine: 3535, 5000, 9020
Rifkin, Dena E.: 9500
Rijal, Rishikesh: 9000
Rijnhart, Judith J M.: 7360, 9010
Riley, Alicia: 1230
Riley, Julie: 5075
Riley III, Joseph L.: 7630
Ring, Lauren: 3530, 9030
Ring, lia: 9420
Ríos Rincón, Adriana: 9000, 9020
Ripat, Jacquie: 7940
Rippon, Isla R.: 4070, 9020
Riser, Tiffany J.: 3240
Ritchey, Katherine C.: 9030
Ritchie, Christine S.: 2035, 4460, 7680
Ritchie, Craig: 7630
Ritchie, John: 7060, 7190
Ritona, Michelle K.: 7620
Ritter, Ashley Z.: 3530, 9030
Rittereiser, Jennifer C.: 7620
Ritz, Beate: 7370
Rivera, Donovan: 9580
Rivera, Lucia: 7820, 8900
Rivera-Hernandez, Maricruz: 4420
Rivera-Leon, Adrianna M.: 3460
Rizzi, Ester L.: 4530
Rizzo, Matthew: 7510, 7620
Roa Diaz, Stephanie: 7110
Robbins, Ronna: 7350
Robbins, Sarah J.: 4080
Roberson, Tonya: 2355
Robert, Stephanie A.: 2230
Roberto, Karen A.: 1050, 4140
Roberts, Amy R.: 1100, 5175, 9010
Roberts, Angela: 7700
Roberts, Ashley: 1310
Roberts, Eric: 3220
Roberts, Jennifer D.: 7560
Roberts, Lisa: 4415
Roberts, Mitchell: 9000, 9010, 9020
Roberts, Penny: 9030
Roberts, Scott: 4210
Roberts, Shelby: 3055, 3300
Roberts, Tonya J.: 3175, 3180, 7770
Robertson, Caitlin: 9010
Robertson, Kurt: 9420
Robinson, D'Artagnan M.: 7700
Robinson, Erin: 1310, 2055, 3565
Robinson, Kristen: 7780
Robinson, Max: 7110, 7880
Robinson, Rena S.: 9020
Robinson, Sara R.: 9010
Robinson-Ector, Kaitlynn: 9020
Robinson-Lane, Sheria G.: 1140, 7680, 9020
Robison, Julie: 1240, 3165, 4140
Robison, Raele: 9020
Robitaille, Annie: 3260
Robles, Cindy: 9030
Robson, Karen: 2030
Roche-Dean, Maria: 3105, 7800
Rochester, Lynn: 3550
Rochon, Elizabeth: 4460, 7680
Rodakowski, Juleen: 7300
Roden, Elena: 7090
Rodniam, Jaroonrat: 2385
Rodrigue-Rouleau, Philippe: 1200
Rodrigues, Edna Darlene: 2215, 7320
Rodrigues, Isabel B.: 4268, 7220, 7680, 7710, 9020
Rodrigues, Jessica: 7320
Rodrigues, Nathaniel: 4495
Rodriguez, Adriana: 9000
Rodriguez, Bianca: 8900
Rodriguez, Juan: 3055
Rodriguez, Miriam J.: 2180, 5070, 7990
Rodriguez, Misha: 3530, 9030

Rodriguez, Rachel L.: 3250, 4095
Rodriguez, Rena M.: 7180
Rodriguez, Tania M.: 9030
Rodriguez-Artalejo, Fernando: 2320
Rodriguez-Fernandez, Imilce A.: 1380
Roe, Lauren S.: 7650
Roecke, Christina: 3115
Roeland, Eric J.: 7680
Roets, Lieve: 3340
Rogalski, Emily: 9030
Rogers, Callie: 4120
Rogers, Gail: 1290
Rogers, Geoff: 3375, 4215
Rogers, Priscilla A.: 7810
Rogers, Wendy A.: 1120, 1260, 1315, 4415, 7680, 9030, 9540, 9580
Rogina, Blanka: 1325, 2065, 5060
Rogus-Pulia, Nicole: 9020
Roh, Soonhee: 7600
Rojas, Julio: 1380
Rolland, Yves: 2320
Rollman, Bruce: 5175
Roman, Anna: 3250
Romanelli, Meghan: 2310
Romanescu, Laura: 9400
Romo, Enelda: 7180
Ronan, Ana B.: 1290
Rooks, Ronica N.: 7960
Rooney, Mary R.: 3390
Roosan, Don: 1095
Root, Eve Z.: 3250, 7590
Roque, Nelson A.: 3480
Roquet, Angélique: 7080
Rorai, Vanessa: 1270, 4150, 7930
Rorick, Leann: 2030
Rosa, William E.: 7900
Rosano, Caterina: 2155, 3475
Roscizewski, Alexa: 7770
Rose, Grace A.: 4095, 7340
Rose, Karen M.: 1300, 4255, 7090
Roseberry, Krissy: 9010
Roseman, Jeffrey: 3490
Rosen, Adam B.: 7020
Rosen, Francesca C.: 2355
Rosen, Greta: 7820
Rosen, Tony: 1145, 2060
Rosenberg, Ted: 9030
Rosenthal, Marsha: 5055
Rosich, Rosellen M.: 2245
Ross, Jeanette: 9010
Ross, Lesley: 3570, 5050, 7080
Ross, Leslie: 9020
Ross, Sarah E.: 4360
Rosselli, Monica: 4132, 5225, 7480
Rossler, Aleatha M.: 7150
Rosso, Andrea L.: 1015, 2155, 3405, 3475, 4050, 4145, 9010, 9020
Rosteius, Katharina: 2370
Rote, Sunshine: 7530, 9030
Rote, Wendy M.: 9010
Roth, Adam R.: 4500
Roth, David L.: 1035, 2130, 3305, 4170
Roth, Nicole: 1040
Rothermund, Klaus: 3100, 3195
Rothman, Alexander: 5100
Rouanet, Jeremy: 1215
Rouse, Sasha: 3435
Rowan, Julia Margaret: 2220
Rowell, Brigid: 9020
Rowland, Julia: 2260
Royse, Sarah K.: 9500
Royster, Leigh-Anne: 7290
Rozanova, Julia: 2055
Rozelle, Mary: 4266, 7190
Ruan, Lei: 9000
Rubin, Daniel: 9030
Rubinstein, Stephanie R.: 7380
Rubio, Enid: 3560
Rubio, Olivia: 4315
Rubsenson Wahlin, Rebecka: 9030
Rubtsova, Anna A.: 2055, 3565
Rudkin, Kristi: 9020
Rudolph, James: 4290
Rudy, Duane: 7950
Rudy, Ellen: 9000, 9020
Rudzianski, Nicholas: 7440
Rueda Ordoñez, Carlos Joaquin: 4266, 7190
Ruffalo, Leslie: 7910
Ruggiano, Nicole: 4415
Ruggiero, Laurie: 7460
Rughwani, Nisha: 7150
Ruiz, Diana I.: 9420
Rule, Payton: 1255, 3245, 7060, 9020
Rumfelt, Kalee: 2320
Runge, Katharina: 4130
Runzer-Colmenares, Fernando M.: 9010, 9030
Rupp, Hayley: 4268, 7680
Rupprecht, Fiona S.: 2105, 4055
Rusch, Amber V.: 1365, 7910
Rush, Ben: 9020
Rush, Carli: 7960
Rush, Jonathan: 3230
Russ, Tom: 4134, 7620, 9020
Russel, Wynfred: 3240
Russell, Accacia: 9000
Russell, Andrea: 9030
Russell, Beth S.: 1390
Russell, Daniel W.: 7610
Russell, David: 4365
Russell, Elizabeth: 4150
Rutberg, Jennifer L.: 7290
Rutherford, Delaney: 7880
Rutledge, Emily: 1165, 5015
Rutledge, Jocelyn: 7040
Ryan, Alice: 7330
Ryan, Calen: 2285
Ryan, Candace E M.: 9010
Ryan, Joanne: 7300, 7370
Ryan, Kathleen A.: 3550
Ryan, Lesa: 7370
Ryan, Lindsay H.: 5035
Ryan, Susan: 3175
Ryant, Adhe S.: 7830
Ryff, Carol D.: 9000
Ryou, Yeon Ji: 7610, 7930
Ryu, Byeongju: 9030
Ryu, Ju Hyun: 7030
Ryuno, Hirochika: 9010
Ryvicker, Miriam: 9010
Rzeppa, Chloe: 3420, 7080

**S**

Saab, Diane: 9560
Saad, Odelyah: 1005
Saavedra, Antonio: 9020
Sabangan, Jerome: 7180
Sabbath, Erika: 9010
Sabino-Laughlin, Eilleen: 4025
Sacco, Lawrence B.: 1125, 3265, 7170
Sacco, Paul: 2020, 9010, 9100
Sachs, Bonnie C.: 4355
Sachs-Ericsson, Natalie J.: 7030, 7510
Sadak, Tatiana: 2385
Sadarangani, Tina: 1095
Sadeq, Nasreen: 2245, 3430, 7330
Sadeqi, Farima: 4185, 9020
Sadowska, Karolina: 7900
sadowski, Cheryl: 9520
Saeidzadeh, Seyedehtanaz: 4408, 7720
Saenz, Joseph: 7320, 7540, 7600, 9000
Saenz, Samantha: 7290
Saffaran, Pouria: 1390
Saffel-Shrier, Susan: 9000
Safford, Monika M.: 2060
Sagara, Tomoya: 7550
Sagi, Doron: 4125
Sagong, Hae: 9010
Sagrak, Adriana: 3270
Saha, Troyee: 7730
Said, Adam: 9000, 9020
Saif, Nadia: 7340
Saint Onge, Jarron: 1185
Sainz, Mayra R.: 2180, 3485
Saito, Taeko: 7620
Saito, Yasuhiko: 3380
Saka, Suleiman: 7520
Sakai, Haru: 7080
Sakai, Keiko: 9030
Sakamoto, Shota: 9000
Sakata, Kei: 4385
Sakib, Nazmus: 9000
Salari, Sonia L.: 4500
Salas, Joanne: 7150
Saldarriaga, Gabriel: 9570
Saldarriaga-ruiz, gabriel: 9570
Salecker, Mariana C.: 3140
Saleem, Zainab: 1200
Salemi, Jason L.: 9010
Sales, Juarez Fayriston Coelho: 7130
Salgado, Glaucia: 9020
Salinas Navarro, Sheila A.: 4375, 7750
Salisbury, Dereck: 9200
Salmon, Adam: 9000
Salom-Bail, Shirley: 7460
Saltzman, Barbara: 7540
Salvo, Erin: 3375
Salwi, Shreya M.: 4425
Salzman, Michele: 4120
Samanapally, Harideep: 9000
Samant, Akanksha: 2145
Samper-Ternent, Rafael: 3525, 4010, 4060, 7840
Sample, Alanson P.: 7820
Sampson, Elizabeth l: 4440, 4470
Sampson, Laura: 2105, 7550
Sams, Richard: 9010, 9500
Samuel, Laura J.: 1345, 5130
San Roman, Irene: 9020, 9520
Sanchez, Claudia A.: 7600
Sanchez, Krystal: 7100
Sanchez, Shena: 7620, 9000, 9020
Sanchez, Victora: 1295, 4110, 7960
Sánchez-Castro, Paulina: 4395
Sanchez-Reilly, Sandra: 7180, 9000, 9010

Sánchez Sánchez, Juan Luis: 2320
Sandberg, Linn: 1250
Sandblom, Gabriel: 9030
Sanders, Edie C.: 7080
Sanders, Jeff: 7620
Sanders, Lisbeth: 7060, 7460, 7620
Sanders, Margaret B.: 7250
Sandesara, Bhanu: 9580
Sandoval, Moises H.: 3465
Sands, Laura: 5195, 7400
Sands, Talyah: 3055
Sanfilippo, Nina A.: 7420
Sanford, Jon a: 1120
Sanger, Stephanie: 7710
Sanon, Martine: 4266, 7190
Sansing, Ann: 7590
Santamaría-Ulloa, Carolina: 9010
Santanasto, Adam J.: 3475, 4260, 5090, 5210
Santarossa, Melanie: 9030
Santilli, Josie N.: 4375
Santora, Kenneth E.: 3040 3185
Santoro, Nanette: 5155
Santos-Lozada, Alexis R.: 4500
Sapkota, Krishna P.: 3380, 7140
Sappington, Erica: 9000, 9010, 9020
Sarango Cancel, Mariana: 4195
Saraswathula, Varun: 7860
Sardina, Angie L.: 2275, 9020
Sargent, Lana: 5030, 9010
Sarid, Orly: 2345
Sarif, Nawaj: 7200, 7940
Sarkar, Nilanjan: 9010, 9030
Sarmiento, Cassandra Vanessa: 4240
Sarquis-Adamson, Yanina: 4120
Sasdi, Olivia: 9000
Sason, Adi: 7720
Sato, Shogo: 4335
Satz, Skye A.: 5135
Saucedo, Jennifer: 7450, 7890, 7990
Sauer, Jacquelin: 4510
Sauer, Jennifer: 5040
Sauerteig-Rolston, Madison R.: 3230, 4535, 9010
Saunders, Gabrielle H.: 4110
Saunders, Michele J.: 3540
Saunders, Pamela: 3200
Saunders, Stephanie: 7170
Sauter, Julia: 1220
Savage, Blake: 7080
Savettiere, Adriana: 9010
Savitz, Sean: 9010
Savla, Tina: 1050, 3445, 4140, 7510
Savundranayagam, Marie Y.: 3270
Sawyer, Linda M.: 7160, 7180, 9000
Sayed, Shaheen: 9110
Saylor, Anna K.: 7620
Scala-Foley, Marisa: 3045
Scales, Kezia: 3285, 3320, 4165, 9530
Scambray, Kiana: 1170
Scerpella, Daniel L.: 1035, 4170, 5160
Schaaf, George W.: 7370
Schadt, Lili: 7860
Schaefer, Sydney: 9030
Schafer, Markus: 1170, 4020, 4500
Schaie, Warner: 4225
Schechter, Gail: 2355
Schechter, Steven: 9010
Scheffer, Julian A.: 1305, 4495, 9320
Schell, Brent R.: 9020
Schepens Niemiec, Stacey L.: 2275
Schepisi, Ila: 9000
Scher, Clara: 2315, 4210
Scherrer, Jeffrey: 7960
Schicker, Teresa: 9030
Schierer, Allie: 7440, 7460, 7520
Schiloski, Kylie: 7510
Schiltz, Nicholas K.: 4525
Schindler, Nicole: 7150
Schlag, Karen: 9010
Schlesinger, Mark: 2300
Schlueter, Amy: 7640
Schlundt, David G.: 9020
Schmedding, Nicholas: 9010
Schmid, Laura: 7780
Schmidt, Annika: 2370
Schmidt, Brad: 7030, 7510
Schmidt, Laura Katharina: 7830
Schmidt, Seigfried O.: 1030
Schmidt, Steven M.: 2340, 7380
Schmidt-Trucksäss, Arno: 3115
Schmitt, Ana Carolina Basso: 9020
Schmitt, Frederick A.: 9020
Schmitz, Alina: 1220
Schmitz, Kathryn H.: 9010, 9020
Schmitz, Lauren L.: 2285
Schneider, Augusto: 4475, 7110
Schneider, Edward: 3000
Schneider, Haley: 3445, 3540, 7470
Schoellig, Shannon: 7950
Schoen, Julie R.: 1285
Schoenfeld, Alix: 4408, 7230
Schoenfeld, Elinor R.: 5030, 7120, 7680, 9020
Schoenthaler, Antoinette: 7640
Schoepflin, Benedicte: 9030
Scholz, Urte: 4235
Schonfeld, Lawrence: 3560, 9020
Schoonhoven, Lisette: 2280, 4125, 7350
Schork, Nicholas: 5180
Schröder, Valerie E.: 9020
Schrack, Jennifer A.: 2150, 3390, 3475, 3510, 5085, 7110, 7650
Schram, Miranda T.: 1225
Schrauf, Robert W.: 3530
Schriner, Samuel E.: 7370
Schroeder, Isaac: 7080
Schroeder, Matthew W.: 5115
Schubert, Carla R.: 2240
Schubert, Cathy C.: 7130, 7180
Schubert, Frederick: 7510
Schuez, Benjamin: 2370
Schulman-Green, Dena: 2235
Schultz, Hanna: 7590
Schultz, Leslie: 2030
Schultz, Michael: 9020
Schultz, Savannah: 7590
Schulz, Richard: 7530
Schumacher, Benjamin: 3390, 3475
Schumacher, John G.: 5185
Schumann, Annette: 3270
Schumm, L Philip: 1230
Schurr, Stephanie: 3570
Schuster, Amy M.: 3025, 9000
Schuster, Johanna: 9400
Schutte, Debra L.: 9010
Schuurmans, Marieke J.: 4125, 7350
Schwaiger-Haber, Michaela: 5180
Schwartz, Andrea: 7860, 9410
Schwartz, Anna: 7640
Schwartzkopf, Ashley L.: 7130, 7180
Schwasinger-Schmidt, Tiffany: 7330
Schweitzer, Amy: 7350
Schwingel, Andiara: 7300, 7610
Sciamanna, Christopher N.: 9010, 9020
Sconiers, Charlie Kira-Nolan: 8900
Scott, Matthew: 9030
Scott, Rachel K.: 2005
Scott, Robynn: 7180
Scott, Stacey B.: 2230, 4045, 4425, 5165
Scott-Poe, Deneisha: 7950
Scrivano, Rachel: 4085, 7590
Scroggins, Laila: 9000
Seago, Elayna: 7020
Seaman, Aaron T.: 2260, 9020
Seaman, Kendra L.: 1090
Sebastiani, Paola: 1040, 1290, 5090, 5180, 7370
Sebu, Joshua: 4145
Seckin, Gul: 7410
Seetharaman, Kishore: 1335, 2340
Sefcik, Justine S.: 3395, 4005, 9000, 9030, 9400, 9540
Segal, Daniel L.: 7910
Segel-Karpas, Dikla: 3275, 7070
Segelman, Micah: 3365
Segerstrom, Suzanne: 1160, 7510, 9020
Seidler, Rachael: 9000
Seidmann, Vered: 3295
Seitz, Dallas: 7080
Sekher, T V.: 7320
Seligman, Benjamin: 4310, 7940
Selovin, Fabio: 4055
Selzler, Katherine J.: 7620
Semelka, Charles: 9030
Sen, Payel: 2065
Sengupta, Manisha: 1095
Senior, Josh: 7510
Seo, Chang Hyun: 4420
Seo, Jin Young: 5230, 7360
Seo, Minjung: 5190, 7000
Seong, Young Ah: 9030
Sepehri, Katayoun: 7120
Ser, Sarah E.: 7860
Serafica, Reimund: 4040
Serdaly, Christine: 7160
Sereika, Susan M.: 9010
Serpas, Dylan G.: 7070
Serpico, Anthony: 7620
Serra, Monica: 7350, 7910

Serrano-Rubio, Luis H.: 4100
Serrat, Rodrigo: 4330
Serre, Marc L.: 2320
Sesek, Greg: 5025
Seshadri, Sudra: 4100
Sesker, Amanda A.: 7990
Sestito, Samuel F.: 7500
Setlow, Barry: 1030
Setoguchi, Soko: 4105, 7800
Setto, Madison: 9410
Severance, Jennifer J.: 4220, 4360, 5190
Sexton, Andrew: 7830
Shaaban, C.: 9500
Shacham, Mayan: 3275
Shade, Marcia: 4268, 7680
Shadmi, Efrat: 7050
Shah, Avani A.: 7210
Shah, Malvi: 4440
Shah, Raj C.: 7300
Shah, Ravi V.: 7630, 9300, 9330
Shaid, Elizabeth C.: 4545, 9010
Shalev, Daniel: 7900
Shamim, Asma: 1390
Shanahan, Angela: 4134, 7620
Shanahan, Mabel: 9020
Shang, Yanan: 7620
Shanks, Josh: 7660
Shanmuganathan, Vasuki: 1145
Shannon, Jackilen: 7680
Shannon, Shen: 3105
Shapira, Stav: 2345
Sharafkhaneh, Amir: 7180
Sharareh, Nasser: 1155
Shardell, Michelle: 3550, 4080, 4260, 7330, 9000, 9020
Sharit, Joseph: 4268, 7680
Sharma, Kumar: 7660
Sharma, Rachita: 7390
Sharma, Shakshi: 4130, 7370, 7880
Sharma, Shubam: 3360, 4010, 9030
Sharninghausen, Jody: 7540
Sharp, Julia L.: 4010
Sharrett, A. Richey: 2365, 3510, 5085, 7340
Shavit, Yochai Z.: 7020, 7070
Shaw, Benjamin: 3370
Shaw, Clarissa: 2160, 3335, 7300, 7860, 9000
Shay, Kimberly: 4300
Shayya, Ashley: 3365, 7260
Shdo, Suzanne M.: 9320
Shea, Kimberly: 4380
Shea, Kyla: 1290
Shealy, E. Carlisle: 7770
Shean, John: 3055, 5015, 7700
Sheehan, Connor: 2275
Sheets, Debra: 7420, 7470
Sheets, Kerry: 3460
Sheffler, Julia L.: 3520, 7030, 9010
Sheftel, Mara: 3065, 4500
Sheikh, Sophia: 9010
Sheldon, Marisa: 1330, 3045
Shelley, Mack: 7590, 9020
Shelton, Kyle: 3460
Shen, Biyi: 9000
Shen, Huei-wern: 7710, 7760
Shen, Jay: 4075, 5185
Shen, Karen: 5160
Shen, Yan: 7010
Shen, Yuqi: 3500, 4490
Shende, Shraddha: 1315, 9020, 9030
Shenoi, Sheela: 2055
Shepherd, Gayle: 7620
Shepherd, Jewel: 7210
Shepherd-Banigan, Megan: 7620
Sheridan-Johnson, Jackie: 3075
Sherman, Aurora M.: 5035
Sherman, Carey Wexler: 3235
Sherman, Ledric D.: 7410, 7430, 7840
Sherwood, Paula R.: 9010
Shi, Kewei: 9000, 9020
Shi, Sandra: 3565, 9010, 9540
Shi, Tianyu: 7190
Shi, Xingming: 7370
Shichijo, Yuka: 7000, 7150
Shidhaye, Rahul: 9020
Shield, Renee: 9000
Shields, Christopher B.: 9000
Shields, Pamela: 4255
Shields, Rachael: 9030
Shih, Cheng-Yu: 9010
Shih, Regina A.: 1265
Shih, Yao-Chi: 1185
Shilbayeh, Mariam: 4300
Shim, Soyeon: 4040
Shimabukuro, Michio: 2380
Shimizu, Yosuke: 9030
Shimon-Raz, Ortal: 9420
Shin, Dongsoon: 7810
Shin, Esther: 7550
Shin, Hye Ri: 9540
Shin, Hye-Won: 2330, 4420
Shin, Hyung Eun: 7380, 7630, 9020
Shin, Isaac: 9030
Shin, Jeongwon: 7420
Shin, Jinhee: 7720
Shin, Jiyoung: 7080, 9020
Shin, Juh Hyun: 7160, 7300, 7800, 9020, 9030
Shin, Jung-hye: 9030
Shin, Marlena: 2145
Shin, Myoung Hwan: 7850
Shin, Oejin: 9010, 9030
Shin, Yeonsoo: 7510, 7560
Shin, Yun Chan: 7170
Shippee, Tetyana: 3010, 3175, 3240, 3305, 3460, 4140, 5025, 5170
Shireman, Theresa: 4000
Shively, Carol: 4160
Shivers, Stephani: 7300
Shnall, Adriana: 1145
Shnayder, Oksana: 7000, 7500
Shochat, Tamar: 4265
Shoemaker-Hunt, Sarah: 7810
Shoenfelt, Alayna: 9010
Shorr, Ronald I.: 3500
Shovali, Tamar E.: 3150
Shreeniwas, Sudha: 5110
Shrestha, Aman: 3380, 5220
Shrestha, Sabita: 9010
Shrestha, Shovana: 4280
Shrestha, Srishti: 2240
Shrestha, Sumina: 4545
Shrira, Amit: 1140, 4180
Shropshire, Jeremey: 3210
Shu, Ruixin: 9000
Shugaev, Inna: 7720
Shugrue, Noreen: 1240
Shultz, Ken: 1095
Shulyaev, Ksenya: 4520, 7050
Shum, Eric Ngai Yin: 9030, 9510
Shumaker, Morgan D.: 9000
Shuman, Stephen K.: 3540
Si, Yafei: 4090
Siaton, Bernadette C.: 7800
Sibai, Abla: 9500
Sibille, Kimberly T.: 1030
Sicinski, Kamil: 1000
Siddiqi, Shadab: 4475
Sieber, Frederick: 1370, 9010
Siedner, Mark: 9000
Siegel, Elena O. O.: 9030
Siegler, Eugenia: 5115
Sielert, Charles A.: 7080
Sikes, Kendra: 7370
Sikora, Nadine: 9020
Sikorskii, Alla: 7800
Silberbogen, Amy: 4095
Silberman, Susan: 2315
Silbermann, Rebecca: 7680
Silbersack, Johanna: 9020
Sillito, Carrie: 4500
Sillner, Andrea: 7830
Silverman, Michael A.: 3110, 7840
Silverman-Martin, Wendie: 7110
Silverstein, Hannah I.: 7550, 7900, 9020
Silverstein, Merril: 4085, 4400, 7950, 9030
Silverstein, Nina M.: 2250, 4266, 4435, 7230, 7830
Silvis, Matthew: 9020
Simmons, Alison: 4460
Simmons, Jabrina: 1010
Simms, Evan: 8900
Simon, Corey: 7410
Simon, Darby M.: 7090, 7420, 9010
Simon, Kennedy: 7550
Simonsen, Sara: 7480
Simonsick, Eleanor M.: 2155, 2195, 2240, 3390, 3510, 4040, 4260, 4265, 4490, 5085, 7150, 9020
Simonson, Grace: 9000
Simpson, Darcia L.: 4406, 7500
Simpson, Deborah: 7310
Simpson, Elizabeth: 3165
Simpson, Felicia: 4355, 7630
Simpson, Luke: 7550
simpson, Ryan B.: 1290
Sin, Mo-kyung: 3005, 3490
Sin, Nancy L.: 3135, 3520, 3545, 4320
Sinclair, David: 4455
Sinclair, David A.: 9020
Sindi, Shireen: 3470
Singer, Adam: 9020
Singer, Jonathan: 7830
Singer, Katelyn J.: 7830, 7990
Singh, Maya: 2085
Singh, Mriganka: 9010
Singh, Priyanka: 1095
Singh, Rajeev Ranjan: 7960
Singh, Revika: 2355
Singh, Tanya: 9020
Singleton, Jenny: 9030
Singleton, Meki: 7260
Singsank, Katie T.: 9580
Sirdich, Brianna: 7300
Sirey, Jo Anne: 3560, 7760
Sirohi, Vijay K.: 1380, 7370

Sison, Maria: 1370
Sison, Stephanie DM.: 9410
Sivananthan, Saskia: 1280
Siverskog, Anna: 1250, 7450
Sixsmith, Andrew: 3255, 4245
Sixsmith, Judith: 3255, 4245
Skalla, Emily: 2130
Skemp, Eleanor: 9020
Skemp, Lisa: 9000
Skidmore, Elizabeth: 7360
Skinner, Kelly: 5105, 7300
Skipper, Antonius: 2115, 7290
Skirbekk, Vegard: 9020
Skoblow, Hanamori F.: 3080, 4370
Skow, Laura: 5085, 7340
Skowronski, Jordan: 4465, 7340
Skrajner, Michael J.: 9400, 9550
Skrove, Zachary: 9030
Skrozic, Amna: 9020
Skubic, Marjorie: 9030, 9540
Slater, Craig: 4125
Slattum, Patricia W.: 4360
Slaug, Björn: 2340
Slavich, George: 7960
Slayday, Riki E.: 5165, 7050
Sliwinski, Martin: 1180, 3080, 3245, 3480, 4055, 4185, 4425, 5165, 7050, 7080, 7510, 9010
Sloan, Danetta L.: 4170, 7360
Sloan, Stef: 4195
Sloane, Philip D.: 1210, 3120, 3320, 9020
Sloane, Richard: 1370, 4485, 9010
Slogeris, Becky: 9420
Slolane, Philip: 1210
Slon, Renee E.: 7150
Smagula, Stephen: 5175
Small, Brent: 1085, 7420
Smeekens, Bridget A.: 3505
Smetcoren, An-Sofie: 1385
Smith, Ambrym S.: 1035
Smith, Andre: 7420, 7470
Smith, Andrea B.: 3060, 4400
Smith, Brian N.: 1160
Smith, Brian N.: 9020
Smith, Brigid: 7440
Smith, Caleb: 7210
Smith, Christina: 2160, 9030
Smith, Claire E.: 4490, 7410, 7890
Smith, Dylan: 2230
Smith, Gregory C.: 3170, 7950
Smith, Jacqui: 1170, 3290, 4325, 7610, 9010, 9030
Smith, Jamie M.: 1375, 3415, 9020
Smith, Jason R.: 2240, 2365, 3335, 3510
Smith, Jennifer A.: 2320
Smith, Jennifer L.: 5125
Smith, Jennifer A.: 7630
Smith, Jennifer L.: 9020
Smith, Jessica J.: 7370
Smith, Kasey N.: 9320
Smith, Kate: 7540
Smith, Kathrine A.: 2030
Smith, Kelly M.: 4170
Smith, Ken R.: 4365
Smith, Kristen: 4030
Smith, Lindsey: 1155, 1210, 3320, 9030
Smith, Louisa H.: 9430
Smith, Matthew Lee: 7410, 7430, 7760, 9000
Smith, Michael: 7670
Smith, Nathan: 7360
Smith, Patsy R.: 9020
Smith, Ryan: 3080
Smith, Shelby M.: 7030
Smith, Sherri L.: 9020
Smith, Steven R.: 7620
Smith, Tawny L.: 5235
Smith, Tiffany: 9010
Smith, Trey: 2325
Smith-ray, Renae L.: 9020
Smithwick, Megan S.: 7510
Smolen, Andrew: 3080, 9000
SM-Rahman, Atiqur: 4190
Smyth, Joshua: 7590
Snedeker, Lauren R.: 9030
Sneed, Rodlescia: 1270, 3080, 4520, 4545, 5215
Snider, Joy: 9550
Snigurska, Urszula A.: 7860
Snitz, Beth E.: 9500
Snow, A Lynn: 3365
Snow, A Lynn: 4290, 7540
Snyder, Jason S.: 1105
Snyder, Joel S.: 3485, 9010
Snyder, Rani E.: 2200, 4295
So, Christine: 1235
So, Hon Yiu: 5070
So, Mardon E.: 7020
Soares, Erica Paci: 7920
Soares, Felipe Paci: 7920
Sofios, Alexandros: 3115
Sog, Zeyuan: 5180
Sol, Ketlyne: 1170
Solberg, Laurence M.: 7860
Solch-Ottaiano, Rebecca: 9010
Soliman, Elsayed Z.: 3390
solimeo, Samantha: 9020
Solway, Erica: 1285
Somerville, Ceara: 4150
Sommerlad, Andrew: 4515
Sommers, Maruska: 3090
Som-Pimpong, Hadassah: 9030
Son, Jung Yoen: 7510
Son, Surim: 4120
Song, Eunjeong: 7810
Song, Jiyoun: 9010
Song, Kijun: 7080
Song, Lixin: 4380, 5055, 5120, 7660
Song, Mi-Kyung: 7900, 7960
Song, Qian: 7270, 9030
Song, Rhayun: 7660, 7810
Song, Richard W.: 1215
Song, Ruopu: 5080
Song, Shijie: 1245
Song, Si Young: 7040, 7080, 7610
Song, Woojin: 4465
Song, Xiao: 7930
Song, Xiaokang: 1245
Song, Xiaowei: 7120, 7160
Song, Yimeng: 3115
Song, Youqiang: 7610
Song, Yuting: 4515
Song, Zeyuan: 5090, 5180, 7370
Soni, Suha: 7840
Sonnega, Amanda: 2045, 3065, 3095
Sonnenfeld, Mandi: 7850
Soong, Daniel: 1010, 9020
Sorkin, John: 7800
Sorra, Joann: 2375
Sosa Cifuentes, Daniela Belen: 4406, 7000
Sosa-Sarmiento, Paola V.: 7840
Sotka, Susan: 9010
Sotos Prieto, Mercedes: 2320
Souders, Dustin J.: 5050
Soulie, Brianna: 4230, 7060, 7570, 7590
Sousa, Karen H.: 9020, 9030
Sousa, Sarah: 5105
Sousa, Susana: 7300
Sousa, Vivian Cintra: 9020
Sousa Barbalho, Yuri Barbalho G.: 7340
Souza, Kristin F.: 3385
Spangler, Hillary B.: 4268, 7360, 7680
Sparks, Cathi: 1150
Sparks, Diane: 7160
Sparks, Lauren M.: 3475
Spataro, Sebastián: 8900
Speaks, Hannah R.: 9020
Spears, Essence: 9010
Spears, Isaiah: 3520, 4320
Spencer, Rebecca: 9010
Spiers, Gemma F.: 7160
Spigner, Clarence: 4408, 7700
Spilsbury, Karen: 2375, 7720
Spira, Adam P.: 2135, 2240, 3500, 4265, 4355, 4490, 7870
Spiro, Avron: 1160, 3155
Spiteri, Raymond J.: 1200
Splain, Ashley: 4130
Spradley, Laura: 7150, 7540, 7550
Sprecher, Bryce: 9030
Spreng, Nathan: 7100
Springgate, Benjamin: 1020
Springstein, Tabea: 1105
Squillace, Marie: 2375
Squires, Allison: 9000
Sreenivas, Kiran: 9020
Srejon, Refat: 7980
Srinivas, Vijaya: 9020
St. John, Philip: 5220
St. Louis, Renee M.: 9030
Staben, Omar: 9010
Stahl, Sarah T.: 5175
Staley, Mark: 7000
Stallard, Eric: 5110, 7620
Stanik, Christine: 9020
Stanley, Jennifer: 7040, 7520
Stansbury, Kim L.: 7230
Stapleton, Tadhg: 7690
Stark, Susan L.: 7780
Stark, Susy: 4406, 7000
Starkweather, Angela: 7460
Staudacher, Sandra: 3180
Stavrinos, Despina: 5050, 7060
Stawski, Robert S.: 4425, 7580
Stearns, Melanie: 3420
Stebbins, Rebecca: 3225
Stechmiller, Joyce: 9030
Steele, Joel S.: 3535
Steele, Maritza V.: 3460
Steele, Tatum P.: 7080
Steer, Christopher: 2260, 9010
Stefancic, Ana: 1335
Steffens, David C.: 4140, 7030
Stegemoller, Elizabeth: 7830
Stegen, Hannelore: 1065, 1385, 7280

Stehouwer, Coen DA.: 1225
Stein, Benjamin: 9030
Steiner, Victoria: 7540
Steinhauser, Karen E.: 4165
Steiniche, Tessa: 9020
Steinman, Bernard A.: 7500
Steinman, Erika: 9540
Steinman, Michael: 5230
Steinmann, Gijs: 2370
Stelle, Charlie: 4390
Stellman, Jeanne M.: 1160
Stellman, Steven D.: 1160
Stenholm, Sari: 7170
Stennett, Christina A.: 4080
Stenvold, Dorthe: 9020
Stephan, Abigail T.: 3060, 7080
Stephen, Alexander J.: 7160
Stephens, Caroline: 4365
Stephens, Jacquelyn E.: 9020
Stephenson, Jane: 9000
Stepick, Lina: 3285
Stepniak, Catherine: 4275
Steptoe, Andrew: 1255, 2015
Stern, Yaakov: 9020
Sterns, Harvey L.: 3000, 3325
Stessman, Jochanan: 2015, 7300
Stessman-Lande, Irit: 2015
Stetten, Nichole: 1030
Stevens, Alan B.: 2060, 7400, 7850
Stevenson, Heather L.: 7860
Steward, Andrew: 4195, 7590
Stewart, Meagan: 1235
Stiefler, Susanne: 2370
Still, Catherine: 2070, 2170, 7800
Stinchcombe, Arne: 3570, 7060, 7450
Stine, Jonathan G.: 9010
Stirland, Lucy E.: 5220
Stites, Shana D.: 3005, 7650
Stival, Marina Morato: 7130
Stock, Braden: 7060
Stockwell, Ian: 9000
Stodola, Allyson: 9010
Stoeckle, Rebecca J.: 2030
Stokes, Jeffrey E.: 2245, 3080, 4055, 4510
Stolic, Snezana: 9000
Stone, Jamie: 9030
Stone, Katie L.: 3500, 7650
Stone, Robyn: 3120, 9020, 9550
Stone-Bury, Lisa E.: 4445, 7910
St-Onge, Marie-Pierre: 4355
Storm, Sierra: 9010
Stout, Jeffery: 9010, 9020
Stout, Jeffrey: 5075, 7080, 7150, 7430, 7680, 7820, 9030
Stoye, George: 2255
Strader, Sydney: 7110
Strand, Bjorn Heine: 9020
Strassheim, Quinn: 7880
Strath, Larissa: 9010
Stratton, Lauren: 1085, 7540, 9020
Strayer, Andrea: 2170, 2180
Streb, Carolyn: 9010
Stringer, Wilma: 4150
Stringfellow, Mary K.: 9010
Strong, Jessica: 7380, 7590, 9020
Strong, Joe: 9000
Strotmeyer, Elsa S.: 5155, 7650
Stroud, Jared: 9020
Strough, JoNell: 1090, 7920
Struckmeyer, Linda R.: 9020
Stuart, Nicole S.: 3520, 4320
Stubbs, Brianna: 7110
Stuckenschneider, Tim: 7830
Stuhr, Nicole: 9020
Stump, Timothy E.: 5015, 9000
Sturdevant, Diana: 9020
Sturla, Shana J.: 9020
Su, Chin-Yi: 7030, 7960
Su, Jingjing: 5185
Su, Kelly: 8900
Su, Mingzhu: 9000
Su, Wei-Hsuan: 7370
Su, Yan: 4505
Su, Yan-Jhu: 3385, 4266, 7230, 7610, 7830
Su, Yun: 7370
Suarez, Jethro Raphael M.: 9030
Suarez-Rodriguez, Luis: 7860
Subedi, Janardan: 5220
Sublett, Jennifer: 7520
Subramanian, Sandeep: 9030
Subramanian, Tejas: 7900
Sucic, Joseph F.: 7150
Sudore, Rebecca: 2035, 4035
Suemoto, Claudia Kimie: 3160, 7320
Sugiyama, Mika: 7280
Suh, Heather: 5070, 9020
Suh, Yousin: 3215
Suitor, J Jill: 1050, 4275, 7920
Suizzo, Marie-Anne P.: 7470
Sukumar, Kavitha: 9030
Sullivan, Dennis H.: 7180, 9000
Sullivan, Jennifer L.: 2145
Sullivan, Kevin J.: 9200
Sullivan, Suzanne S.: 2110, 7260, 7620
Sultana, Munira: 4120
Sultana, Samia: 9000
Sumikawa, Yuka: 7160, 9520
Summerhayes, Emily: 8900
Sun, Chuxuan B.: 1115
Sun, Fei: 1095, 2085, 2330, 4155, 7010, 7560, 9000, 9010, 9030, 9300, 9530
Sun, Haosen: 4020
Sun, Juanjuan: 4155
Sun, Minjung,: 7330
Sun, Peter C.: 4085
Sun, Qi: 1290
Sun, Sicong: 4305
Sun, Winnie: 9010, 9520
Sun, Xin: 4132, 7500
Sun, Yang: 7030
Sun, Yixuan: 7390
Sundaresan, Lakshmi: 2225
Sunderman, Erin E.: 4340
Sung, Mira: 9020
Sung, Pildoo: 3435, 4020, 4025, 4175
Sunil, Surya: 9000
Sun-Mitchell, Shan: 9030
Suntai, Zainab: 3005, 4275, 4510, 7900
Suorsa, Kristin: 7170
Supiano, Katherine P.: 1025, 1150, 2035, 4406, 7000, 9020
Surachman, Agus: 5130, 9440
Suri, Sachin: 9020
Suriaga, Armiel A.: 7580
Surood, Shireen: 7520
Sussman, Tamara: 4440
Sutherland, Laura: 9000
Sutin, Angelina R.: 3245, 4035, 4055, 7990
Sutphin, George L.: 7870
Suwanpasu, Sunee: 7860
Suzuki, Hiroyuki: 7550, 7760
Suzuki, Makoto: 2380
Svartengren, Magnus: 7170
Svec, Joseph: 1055, 2075, 3360, 4390, 7610
Svedberg, Pia: 9030
Swagger, Phildra: 7810
Swain, Joseph: 7210
Swanepoel, Lizette: 7690
Swanson, Helena L.: 7250
Swanson, Leslie: 5155
Swartz, Ann: 4268, 7680
Swartz, Kristine: 7500
Swartzell, Kristen: 4210, 5105
Swenor, Bonnielin K.: 1345, 2365
Swift, Hannah J.: 1200
Swift, Henry O.: 4150
Sy, Maimouna: 7680
Syed, Ayesha: 9020
Sylvester, Hunter: 5085, 7340
Syme, Maggie L.: 9030
Symes, Bobbi: 5215, 7730
Szanton, Sarah L.: 1375
Szanton, Sarah L. L.: 1345, 2290, 3240, 4310, 5130
Szymkowski, Amanda: 7310

T

T R, Dilip: 4385
Taani, Murad H.: 1190, 7610, 7640
Tabata, Hiroki: 9000
Tabrizi, Maryam: 4075, 7310
Tadel, Pat M.: 7290
Tadesse, Ruth S.: 7440, 7470, 7540
Taeckens-Seabaugh, Ashley: 5200
Taffet, George E.: 4160
Taha, Hash Brown: 9000, 9010
Tahashilder, Md Ibrahim: 4395
Tai, Chiu-Mei: 4155
Takahashi, Tomoya: 7550, 7760
Takaoka, Manami: 9520
Takase, Mai: 7610
Takase, Mai: 9030
Takeda, Masatoshi: 9030
Tal, Noa: 1125
Talbot, H. Keipp: 4130
Talegawkar, Sameera: 9020
Talley, Kristine M.: 9030
Talwar, Sonia: 8900
Tamayo, Valeria: 9000
Tambor, Ellen: 2030
Tamburri, Nicholas: 7420, 7470
Tamres, Lisa: 9020
Tamura, Yoshifumi: 9000
Tan, Alai: 3345
Tan, Chuen Seng: 4385
Tan, Erwin J.: 5175
Tanaka, Clarice: 9010
Tanaka, Katsuya: 2105, 3220

Tanaka, Toshiko: 4490
Tanase, Alexis D.: 5230
Tang, Ada: 5100, 7180, 7340
Tang, Dan: 4155
Tang, Esther Y. K.: 9010
Tang, Fei: 9030
Tang, Fengyan: 2090, 4050, 7600, 9020
Tang, Huameng: 9030
Tang, Jennifer Yee-man: 7330, 7760
Tang, Junhan: 4090
Tang, Lu: 5195
Tang, Paul: 4035
Tang, Vivien Foong Yee: 1065
Tang, Xueting: 9010, 9020, 9300, 9550
Taniguchi, Yu: 7280
Tanilli, Rachel: 4408, 7230
Tappen, Ruth M.: 2180, 4132, 5225, 7480, 7580
Tarabochia, Dawn: 5075, 7910
Tarbert, Rebecca J.: 9020
Tariq, Suleman: 4268, 7220, 7680
Tarraf, Wassim: 7960, 7970, 9020, 9210
Tasyurek, Ezgi: 1200
Tate, Channing E.: 4408, 7260
Tate, Judith: 4255, 9010, 9030
Tate, Kaitlyn: 4408, 7720
Tate, Robert: 7940
Tauseef, Mahruhk: 9010
Tavares, João: 7300
Tavares, Karina L.: 1350
Tay, Djin L.: 4365
Tay, Laura Bee Gek: 7760
Tayag, Paolo: 5065
Taylor, Ashley: 9000
Taylor, Christopher A.: 4130
Taylor, Courtney: 4195
Taylor, Harry: 4030
Taylor, Janelle S.: 7690
Taylor, Janie: 7460
Taylor, Janiece L.: 2290, 3240
Taylor, Kate: 4360
Taylor, Philip: 3285, 4345
Taylor, Simeon: 4080
Taylor, Tonya N.: 3565
Taylor, Zachary E.: 3545
Tazaki, Fumie: 9030
Teaster, Pamela B.: 7770
Teerawattananon, Yot: 3440, 4385
Teerawichitchainan, Bussarawan: 2050
Teixeira, Laetitia: 3130, 7060, 7200, 7300, 7320
Teixeira, Samantha: 3095
Tejeda, Carlos: 9000
Teles, Soraia: 4406, 7000, 7300
Tell, Eileen J.: 1175, 4295
Telonidis, Jacqueline: 5190
ten Cate, Debbie: 4125, 7000, 7350
Tennyson, Robert L.: 9030
Teo, Wai Choo: 7760
Teply, Abby L.: 7590, 7610, 7840
Teran, Dianne: 2160
Teresi, Jeanne A.: 1145, 2030, 2060
Terhune, Elizabeth: 9010
Teri, Linda: 5135
Terracciano, Antonio: 3245, 4035, 4055, 7990
Terrill, Alexandra L.: 1150, 7700
Terry, Melita: 9020
Teshale, Salom M.: 4295
Teshome, Abraham: 7190, 7200
Tessler Lindau, Stacy: 7230
Tetlow, Hilary: 9000
Tewarson, Hemi: 2200
Tewary, Sweta: 7000, 7500
Thabane, Lehana: 7180, 7340
Thaitrong, Phatt: 7620, 9000, 9020
Thakkar, Nirali: 7430
Thakkar, Priyanka: 9330
Thangwaritorn, Pilar: 4134, 7330, 7830
Tharrington, Shafer: 9030
Thayer, Erin K.: 2220
Thayer, Julian F.: 1215
Thayer, Rachel E.: 7410
Theeke, Laurie A.: 1070, 5225, 7330, 9200
Theng, Yin-Leng: 3085, 3295, 3470
Theos, Gregory: 9020
Thiamwong, Ladda: 2385, 5075, 5100, 7080, 7150, 7430, 7620, 7680, 7820, 9000, 9010, 9020, 9030
Thielke, Stephen: 7300
Thierry, Amy: 2115
Thomas, Kali S.: 1210, 3320, 4030, 4290, 5215, 7160, 7210, 9030
Thomas, Karuna S.: 7550
Thomas, Kelsey: 8900
Thomas, Michael: 7980
Thomas, Michael J.: 9430
Thomas, Patricia A.: 4535
Thomas, Phyllis: 7380
Thomas, Shanice: 4110
Thomas Hebdon, Megan C.: 7980
Thomasson, Whitney: 7540
Thomeer, Mieke Beth: 4520
Thompson, Alisha R.: 3455, 7280, 9020
Thompson, Amber: 1150, 9010
Thompson, Atalie: 5230
Thompson, Carl Anthony: 7720
Thompson, Genevieve: 4470
Thompson, Hilaire: 7150, 7280, 7460, 7480
Thompson, Katherine: 7230, 9010
Thompson, LaDora V.: 3150
Thompson, Lena E.: 2120, 3015, 3445, 7470
Thompson, Louisa: 7100
Thompson, Mika D.: 9000
Thompson, Roy A.: 7140, 9000, 9020
Thompson, Sheila M.: 3050
Thompson, Tess: 1255
Thomson, Cynthia A.: 2260
Thomson, Katie: 7160
Thorpe, Anna M.: 3505
Thorpe, Carolyn T.: 7300
Thorpe, Lorna: 7230
Thorpe, Roland J.: 1345, 3240, 3450, 7560, 7970, 9000
Thunell, Johanna: 1175
Thurston, Rebecca C.: 5155, 5225
Thyagarajan, Bharat: 2325, 9020
Tian, Chong: 3490
Tian, Jiayin: 4155
Tian, Junyan: 9020
Tian, Qu: 2155, 2195, 3390, 3510, 5085, 9020, 9300
Tibbitts, Deanne C.: 7680
Tighe, Caitlan: 7580
Tillmann, Jacob J.: 7180
Tilsen, Rachel: 9000
Tinetti, Mary: 4010, 7840
Ting, Peng-sheng: 7390
Tingle, Sharna R.: 9030
Tinney, Emma: 7100
To, Louis: 9010
To, My Ngoc: 2090, 2330, 9010
Tobey, Leah R.: 7390
Tocchi, Christine: 9020
Toda, Eiji: 2105, 3220
Todd, Adam: 4455
Todd, Chris: 4455
Todd, Christopher: 4110
Todorova, Valentina K.: 1085
Toh, Wei Xing: 9000
Tolea, Magdalena I.: 2125, 3490
Toledo, Frederico G.: 3475, 5210
Toles, Mark: 4380
Toman, Julia: 4110, 7960
Tomarevska, Olena S.: 7820
Tomaz, Simone: 7060, 7190
Tomlin, Nichelle: 8900
Tomlinson, Sam: 9020
Tompkins, Catherine J.: 1335, 3310, 7750, 7760
Tong, Yao: 9000
Tongsiri, Sirinart: 2385, 7430
Tongu, Yoshiyasu: 7370, 9010
Tonkikh, Orly: 9030
Tonkin, Andrew: 7300
Tooth, Leigh: 1370
Topaz, Maxim: 9010
Topchishvili, Davit: 2315
Toprakkiran, Selin: 9020
Torigoe, Trevor: 2380
Torres, Anderson: 9000
Torres, Haydee: 8900
Torres, Sandra: 3165, 3530, 4285, 4330
Tosato, Matteo: 9020
Toshiko, Tanaka: 9020
Tosi, Dominique: 9030
Tosi, Marco: 4400
Toto, Pamela E.: 5000, 7800
Totten, Keliane: 4460
Townsend, Aloen: 7940
Towsley, Gail L.: 7700, 9020, 9030
Toyama, Itsuko K.: 3210
Toyokawa, Noriko: 7950
Trabut, Loïc: 7730
Tracy, Karen: 9000
Traeger, Lara: 9030
Trahan, Evette: 9000, 9010
Trahan, Julia: 9020
Tran, Diep Q.: 7210
Tran, Dieu-My T.: 4040, 4134, 7330
Tran, Karenna: 9010
Tran, Nathaniel M.: 1180, 4000
Tran, Tracy S.: 9000
Trautman, Lana: 1180
Traver, Anthony C.: 1205, 3410, 4305
Travers, Jasmine: 7120, 9020

Traywick, LaVona S.: 5190, 7680
Treat-Jaconson, Diane J.: 9410
Tredinnick, Ross: 9030
Trenary, Tanner T.: 7820
Triantos, Lisa: 7860
Trimble, Shawn: 2355
Trimboli, Tracy: 5030, 7120
Trinh, Mimi: 9580
Trinkaus, Eva-Maria: 1005
Triolo, Federico: 2280
Trivedi, Ranak: 3030, 4095, 5000, 9000, 9010, 9030
Troutman, Brooke: 2390, 3305
Troxel, Wendy M.: 3405, 9010
Trubits, Em F.: 7450, 7890, 7990
Trueblood, Amy: 7140
Trujillo, Miguel: 4195
Truskinovsky, Yulya: 1340
Tsai, Alexander C.: 9000
Tsai, Jenny Hsin-Chun: 4408, 7700
Tsai, Pao-Feng: 9010
Tsai, Tsung-Yu: 9010
Tsai, Yi-Hua: 7610
Tsang, Chung-Kin: 9030, 9510
Tsang, Sze Pui: 1110
Tsay, Pei-Kwei: 4155
Tsay, Ruey-Ming: 9010
Tschoe, Marianne: 7520
Tse, Dwight: 1245, 4180
Tseng, Heidi: 3060
Tseng, Huai-Hsuan: 9010
Tseng, Tung-Sung: 7390, 7410
Tsimane Health and Life History Project, The: 9030
Tsitsipatis, Dimitrios: 5060, 7880
Tsotsoros, Cindy E.: 1350, 1355, 4375
Tsotsoros, Jessica D.: 1350
Tsui, Emma: 4165
Tsui, Ronald: 9010
Tsukada, Noriko: 3310, 7790
Tsuker, Sophia: 1340, 9030
Tu, Wanzhu: 5015, 9000
Tu, Yufang: 7300
Tucker, Gretchen G.: 7540, 7940
Tucker, Julia E.: 7020
Tucker, Katherine: 9020
Tucker-Roghi, Gina C.: 9020
Tufa, Yeabsira: 3500, 7070
Tufă, Laura: 1220
Tuft, Samantha E.: 2060, 2220, 4215
Tulloh, Anna: 9000
Tumar, Alisha: 3270
Tunalilar, Ozcan: 3320, 7550
Tung, Yi-Hsuan: 1185
Tunney, Orlaith C.: 9030
Tuntland, Hanne K.: 7940
Tupler, Larry A.: 3470, 7620
Tupper, Emily: 4100
Turaani, Mohammad: 9020
Turiano, Nicholas A.: 3135, 9030
Turkelson, Angela E.: 5035, 7390, 9000
Turkheimer, Eric: 7370
Turner, Ani: 9020
Turner, Arlener D.: 7460
Turner, Jennifer R.: 4425
Turner, Natalie: 7230
Turner, R. Scott: 9550
Turner, Rachael: 7510
Turner, Sidney C.: 3220, 7210
Turner, Skyla: 5235, 7030, 9010
Turo, Patricia: 4035
Tur-Sinai, Aviad: 9030
Twardzik, Erica: 2150
Two, Wung Wai: 5225
Ty, Diane: 3055
Tyagi, Pranjal: 1005, 9010, 9030
Tyler, Denise A.: 2375
Tyshkovskiy, Alexander: 7370
Tysinger, Bryan C.: 4385
Tzemah-Shahar, Roy: 3125, 4265
Tzeng, Huey-Ming: 4395
Tziarli, Dimitra: 9000

U

Ubaida-Mohien, Ceereena: 2195
Uccheddu, Damiano: 4400, 4530
Uchida, Hayato: 7330
Udah, Grace: 8900
Uddeen, Aleeyah: 9010, 9020
Udeh, Kingsly C.: 9010
Udoh, Idorenyin I.: 7420
Ugwu, Ejike s: 5110
Uittenhove, Kim: 3130
Ukraintseva, Svetlana: 2015, 7110, 7370, 7620, 7800
Ullal, Akshith: 9010
Ulmer, Cameron: 9020
Ulrey, Page B.: 3375
Ulrich, Connie: 9020, 9030
Ulset, Vilde: 9420
Umar, Tooba: 1120
Umberson, Debra J.: 4370
Undem, Teri: 7300
Unroe, Kathleen: 4470, 5015, 9000
Unruh, Mark A.: 5215
Unsworth, Carolyn A.: 7690
Uphold, Constance: 2170
Upreti, Mona: 3270
Upshaw, Jenica N.: 3535
Ura, Chiaki: 7280
Urbaniak, Anna: 4450
Urbanski, Dana P.: 3460, 7380
Urbanski, Paul: 7450
Urbina, Ray: 2015
Ureña, Stephanie: 2050
Urick, Benjamin: 7150
Urlings, Judith: 7720
Urrunaga-Pastor, Diego: 9010
Ustach, Andrew: 7480
Utter, Kierstin: 7330
Utz, Rebecca: 1150, 2035, 4365, 9020

V

Vaca Angus, Carlos A.: 7510
Vacek, James L.: 7990
Vacek, Sarah: 7420
Vahedi, Vanessa: 1165
Valdes, Elise: 7290
Valdes-Hernandez, Pedro: 9010
Valdez, Gregorio: 2335
Valdez, Rupa: 2070
Vale, Michael T.: 4320
Valeika, Sarah: 2055
Valente, Kayla: 7800
Valente, Matthew: 7360
Valera, Juhi R.: 4425
Van Vleet, Samuel C.: 5065, 5190, 7080
van Achterberg, Theo: 1175, 4255
Van Bogart, Karina: 3545, 9010
van Boxtel, Martin PJ.: 1225
Van den Boomgaard, Iris: 7000
van den Bosch, Arne: 9000
van den Heuvel, Edwin: 5220, 7660
van den Hoed, Monique W.: 9010
Van der Heijden, Beatrice: 7290
van der Steen, Jenny T.: 4440, 4470
van Es, Cat: 3260
Van Fossen, Jenna A.: 9000
van Hout, Hein: 7420
Van Houtven, Courtney: 1115, 4165, 7620
Van Laethem, Lauren: 4350
van Nimwegen, Dagmar JJ.: 2280
Van Ogtrop, Katlynn: 7260
Van Orden, Kimberly A.: 1335
Van Scoy, Laruen J.: 5145
van Solinge, Hanna: 9030
Van Vleet, Bryce A.: 1235, 7710, 7810
van Zon, Sander KR.: 4130
Vance, David Eugene: 3545, 3565, 4145, 4340
Vance, Jarod C.: 9030
Vandekerckhove, Joachim: 5165
Vander Wyck, Brent: 1305
Vander Wyk, Brent: 2360, 7360
Vanderlaan, Courtney: 7640
VanderWerf, Jessica: 2095, 4406, 7000
VandeVrede, Lawren: 1380
VandeWeerd, Carla: 9000, 9010, 9020
Vang, Casper: 3450
VanHaitsma, Kimberly: 3205, 7160, 9020, 9300
Vanhoose, Kimberly: 7730
VanSciver, Angela: 7990
Varadaraj, Varshini: 1345, 2135
Varadhan, Ravi: 1370, 4485, 9010
Varlamova, Maria: 4450
Varricchio, Marcia: 7920
Varu, Ava: 9020
Varzino, Michael: 9540
Vasarhelyi, Krisztina: 9000
Vasquez, Elizabeth: 5195
Vasquez, Felix A.: 7540
Vassar, Matt: 7210, 9000
Vasterling, Jennifer J.: 9020
Vaughan-Sarrazin, Mary S.: 2180
Vaughn, Ivana A.: 2225
Veal, Britney: 7990
Vega, Irving E.: 3105
Velazquez, Janice: 4345
Velazquez, Nathan: 3570
Vellani, Shirin: 4440
Vellas, Bruno: 2320
Vélez Landazuri, Maria Augusta: 7290

Vélez Ortiz, Daniel: 9000
Venditti, Elizabeth M.: 9020
Venegas, Maria D.: 2085, 7850
Venkatraman, Tishya: 2255
Verçosa, Neide: 7920
Verbeek, Hilde: 1260, 2370, 3180, 7120, 7720, 7800
Vercauteren, Toon: 4330
Verderber, Stephen: 9550
Verdery, Ashton: 4500
Vereijken, Beatrix: 3550
Verghese, Joe: 3145, 3160, 3480, 9020
Verma, Ambika: 7100, 7370, 7880
Verma, Mansi: 7940
Vest, Amanda R.: 3535
Vetrano, Davide: 2280
Vetter, Michael: 4430
Victor, Christina R.: 4030, 4070, 9010, 9020, 9430
Victoria-Sanchez, Leslie: 8900
Videla, Emma: 4500
Viggars, Mark: 7370
Vignoli, Michela: 7290
Vikram, Kriti: 7960
Villalba, Madeline: 9000
Villatoro, Alice P.: 5195
Villemagne, Victor L.: 9500
Vincent, Aaron D.: 7590
Vincent, Heather K.: 4134, 7330
Vincenti, Virginia B.: 3075
Visaria, Abhijit: 1055, 7090
Vishnepolskiy, Igor: 7620
Vishwanath, Swarna: 7300
Visovsky, Constance: 3420
Visser-Meily, Johanna: 2280
Vitolins, Mara Z.: 5060
Vivoda, Jonathon: 5105, 9010
Vizuere, William: 2320
Vlaeyen, Ellen: 7120
Vlock, Elizabeth: 7510
Vluggen, Stan: 3340
Vo, Anh: 7590
Vo, Tina T.: 3080, 4185
Vogel, Carlyn E.: 5170
Vogel, Rosi: 2260
Vogelsmeier, Amy A.: 1310, 7140
Voils, Corrine I.: 5000
Volic, Milla: 9010
Volpe, Cris Renata Grou: 7130, 7340
von Bonsdorff, Mikaela B.: 9010
von Gunten, Armin: 3130
Voss, Andreas: 3195
Vranceanu, Ana-Maria: 2035, 3525, 4460, 7680
Vrees, Matthew: 9010
Vu, Nguyen C.: 3380
Vu, Thi: 2055, 4010
Vue, Hope N.: 8900
Vullings, Isabelle: 9010
Vyhnalek, Martin: 7420

W

Wachholz, Patrick Alexander: 3180
Wada, Mineko: 7780
Wadely, Virgina G.: 4340
Wadsworth, Sally J.: 3080, 4185
Waetjen, L Elaine: 5155
Wagenknecht, Lynne E.: 4355, 5060, 7630
Wagg, Adrian S.: 9550
Wagner, Amy: 7650
Wagner, Gert G.: 9010
Wagner, Jennifer: 9000, 9020
Wagner, Laura M.: 3285, 9020
Wagoner, Aaron: 9030
Wagstaff, Ruth: 9000
Wahl, Hans-Werner PD.: 4225, 4235
Wahlheim, Christopher N.: 9030
Waite, Linda J.: 1230, 4205
Wakefield, Dorothy: 1240, 4140
Walbaum, Magdalena: 4455
Walden, Allison: 4268, 7680
Walden, Justin: 9000
Waldestein, Shari R.: 4130
Waldman, Zoe: 7070
Waldron, Danielle A.: 4510
Walke, Lisa M.: 7860
Walker, Alan: 4450
Walker, Drew: 9400
Walker, Jenny: 9010
Walker, Kathryn A.: 4170
Walker, Kealie J.: 9020
Walker, Keenan: 2195, 2240, 3495, 4035, 7110, 7630, 9200
Walkner, Tammy: 9020
Wall, Jaclyn: 9020
Wallace, Andrea: 1155
Wallace, Cara L.: 3395
Wallace, Laura: 9010
Waller, Dylan E.: 2085, 7850
Wallhagen, Margaret I.: 2135, 2280
Wallimann, Miriam: 4480
Wallner, Lauren P.: 1085
Wallsworth, Christine: 3270
Walmsley, Sharon: 4340
Walsemann, Katrina M.: 1000, 2050, 5050
Walsh, Christine A.: 1205, 9010
Walston, Jeremy D.: 1370, 7110, 9000, 9010, 9020
Walters, Anna: 3535
Walton, Surrey: 4465
Wammes, Joost: 7120, 7420, 9010
Wan, Debby: 9010
Wan, Emily Y.: 5065
Wan, Qiaoqin: 7100, 7620, 7830
Wan, Shaowei: 1240
Wang, Anni: 9020
Wang, Binsheng: 9030
Wang, Claire: 7070
Wang, Cuiling: 5165
Wang, Dahua: 7070
Wang, Danny: 3545, 4145, 7090
Wang, Donna: 9030
Wang, Ellen: 7710
Wang, Ethan B.: 4266, 7690
Wang, Fang: 7080
Wang, Fei: 3005
Wang, Feng: 9010
Wang, Gavin Y.: 9020, 9200
Wang, Grace Hsin-Min: 3500
Wang, Hang: 3390
Wang, Haowei: 1290
Wang, Heng: 4540, 7090
Wang, Janet: 2050
Wang, Jing: 2090, 2265, 3180
Wang, Jing: 7330
Wang, Jing: 7660
Wang, Jing: 9000
Wang, Jing: 9010, 9020, 9300, 9320, 9550
Wang, Julian: 7160
Wang, Jun: 4535
Wang, Junqiao: 9020, 9300
Wang, Kaipeng: 2090, 2330, 9010
Wang, Katherine: 2090
Wang, Kesheng: 9200
Wang, Kun: 7080, 7590
Wang, Lichao: 9030
Wang, Melanie: 9020
Wang, Meng: 3355
Wang, Mengya: 1175, 7930
Wang, Molin: 1290
Wang, Nan: 2265, 9000
Wang, Pengcheng: 2280, 7330
Wang, Qi: 5100
Wang, Qin: 2265, 7330
Wang, Qingwei: 9010
Wang, Rosalie H.: 9330
Wang, Shanshan: 7150
Wang, Shaohua: 7100
Wang, Shiyu: 1215
Wang, Shuang: 2285
Wang, Shuangshuang: 3295, 9030
Wang, Sijiu: 4140
Wang, Silas: 9200
Wang, Siqi: 4540
Wang, Siyu: 9020
Wang, Tzu-Yun: 9010
Wang, Weijun: 7330, 9020
Wang, Wenjin: 9010
Wang, Xia: 9010
Wang, Xiaochuan: 9020
Wang, Xinhui: 2320
Wang, Xiujuan: 9520
Wang, Xueqing: 9440
Wang, Yan: 1030
Wang, Yan: 1110
Wang, Yan: 4210
Wang, Yi: 3210
Wang, Yi: 3450
Wang, Yi: 4050, 7710
Wang, Yi-Hsin: 9010
Wang, Yingying: 9000
Wang, Yixuan: 2330
Wang, Yueting: 5080
Wang, Zequan: 7460
Wang, Zhendong: 5235
Wangwun, Patcharee: 1080, 5200
Wanigatunga, Amal A.: 2150, 3390, 3510, 4265, 5085, 7650, 9580
Wanka, Anna: 2340
Wanninger, Ellen: 9020
Ward, Caitlin: 7860
Ward, R. Scott: 4406, 7000
Ward, Rachel: 9010
Ward, Shaelyn: 7210, 9000
Waring, Stephen: 3460
Warner, Lisa M.: 7610
Warner, Mildred: 7250
Warren, Allison: 4300
Warren, Lorrie: 7480
Warren, Teresa S.: 1260
Warthen, Terra: 7770
Wasenius, Niko S.: 9010
Washington, Faith-Christina I.: 7590
Washington, Karla T.: 1240, 1310, 4415, 9000
Washington, Tiffany R.: 2115, 4270, 4415
Wasserman, Michael: 2295
Wasserman, Michael: 9020
Waszczuk, Daniel: 4310
Watanabe, Kengo: 5180, 7110, 7880
Watanabe, Yuichi: 7780

Watanabe-Galloway, Shinobu: 3535
Waterhouse, Jodi: 2160
Waters, Leland: 3285, 3300, 4360, 9030
Waters, Mary: 4420
Watkins, Alexandra: 9010
Watman, Deborah: 3415, 4165, 7850
Watson, Wendy: 4390
Watts, Amber: 4134, 7330, 7830, 7990
Waugh, Christian E.: 1105
Waye, Mary MY.: 5225
Wazinki, Karla: 2340
Waziry, Reem: 4120, 7630
Weathers, Brandy: 9020
Weaver, Bruce: 3570
Weaver, Michael: 9030
Weaver, Natalie: 7020
Weaver, Raven H.: 1240, 1365, 3250
Webb, Fern: 7810, 9010
Webb, Jason A.: 7680
Webb, Nicole: 2260, 9010
Webber, Sandra: 7940
Webster, Britney A.: 7950
Webster, Noah J.: 1170, 1355, 2100
Webster-Dekker, Katelyn E.: 9440
Wec, Aleksandra: 2185, 3415
Wedderburn, Raymond: 7860
Weech-Maldonado, Robert: 2350, 3365, 4190
Weems, Jacy A.: 1210, 3320, 4190
Wehry, Susan: 9000
Wei, Andrea: 4525
Wei, Duo Helen: 4132, 7480
Wei, Jeanne: 1085, 4130, 7100, 7370, 7880, 9000, 9020
Wei, Jingkai: 1085, 3490
Wei, Kai: 7650
Wei, Linjiang: 9560
Wei, Wenxing: 7940
Wei, Ying: 1110
Wei, Yudan: 9000
Wei, Zhikui: 4490
Weibel, Robert: 3115
Weidenbacher, Hollis J.: 4165
Weil, Joyce: 4405
Weimar, Lisa: 7860, 9000
Weinberg, Gil: 1120
Weinberg, Samuel Jesse: 2225
Weiner, Anthony: 4460
Weinstein, Andrea M.: 3405
Weinstein, Elliott R.: 4340
Weintraub, Susan: 1325
Weiskittle, Rachel E.: 4445
Weiss, Carleara: 7830
Weiss, Charlotte R.: 9020, 9030
Weiss, David: 1245, 3100
Weiss, Erica F.: 1125
Weiss, Joan: 2297
Weiss, Ursula: 9010
Weissberger, Gali: 1140, 4275
Weisse, Carol S.: 3200
Weldrick, Rachel: 1205
Wellens, Nathalie IH.: 7160
Wellenstein, Kerry: 1290
Welling, Anna: 7980
Wells, Jenna L.: 1305
Wells, Jenna L.: 9320
Wells, Katie: 7630
Welsh, Kimberly: 4350
Welsh-Bohmer, Kathleen: 9030
Wen, Chiung-Jung: 4134, 7830, 7930
Wen, Jin: 3520, 3545, 4320
Wen, Ming: 9020
Wen, Wan: 5195, 7030, 7590
Wen, Xinyue: 2330
Wennberg, Alexandra: 5205
Wente, Karla: 5155
Werner, Nicole E.: 2070, 2170, 5000, 9020
Wessel, Leslie: 7390
West, Jessica S.: 2265, 9020
West Greenlee, M. Heather: 7550
Westbrook, Reyhan M.: 9000, 9020
Westendorf, Jennifer: 8900
Westerlund, Hugo: 1125, 3265, 7170
Westmore, Megan R.: 2390, 9000
Westra, Jordan: 9010
Westrick, Ashly C.: 1085, 2280, 7960
Wethington, Elaine: 7460
Wetle, Terrie: 7620
Wheeler, Jason: 9020
Wheeler, Katie: 7060
Wheeler, Victor: 9020
Wherry, Sarah J.: 7350
While, Christine: 4545
Whitaker, Janice: 4315, 5145
Whitbourne, Susan Krauss: 2250
White, C.J.: 1285
White, Elizabeth L.: 3320
White, Katie: 4255
White, Kina: 4266, 7230, 7830
White, Laura: 4406, 7480
White, Lon: 7100
White, Quinn: 5135
White, Rebecca: 4245
White, Thomas: 1325
White Whilby, Kellee: 9020
Whitfield, Keith: 7970, 9000
Whitlatch, Carol J.: 3535
Whitley, Deborah M.: 3060, 4400
Whitman, Cassidy: 7140
Whitmire, Meredith: 1290
Whitmore, Carly: 5220
Whitney, Sarah: 4150
Whitson, Heather: 1370, 2240, 4485, 9030
Whittaker, Anna C.: 7190
Whitten, Justin M.: 5075, 7910
Wi, Dahee: 7480
Wickersham, Karen E.: 2260
Wickson-Griffiths, Abigail: 4440, 4470
Widener, Michael: 9000
Wiegelmann, Henrik: 2370
Wielgosz, Joseph: 9000
Wiemers, Emily: 1340
Wierman, Heidi: 7150
Wiersma Strauss, Anna: 1340
Wiese, Lisa Ann Kirk: 2125
Wijkander, Maria: 9030
Wilber, Kathleen H.: 2220, 3365, 4215, 4375, 4435, 5120, 7720, 7750, 9020
Wilchesky, Machelle: 7080, 7790
Wilcox, D. Craig: 5060
Wild, Tess: 1090
Wilder, Jocelyn: 1230, 4205
Wilkie, Diana: 7380
Wilkinson, Joel: 9020
Wilkinson, Lindsay R.: 7550
Wilks, Scott E.: 7280, 9020
Will, Celia E.: 3345
Willard, Meredith A.: 9030
Willcox, Bradley: 2380, 5060, 7110
Willcox, D. Craig: 2380, 7110
Willett, Walter: 1290
Williams, Aelwyn: 9420
Williams, Alicia R.: 5040
Williams, Alisha: 3480, 9000
Williams, Cedric J.: 9020
Williams, Christine L.: 7620
Williams, Ginger: 9000
Williams, Isaiah: 1325
Williams, Ishan C.: 3305, 9020, 9030
Williams, Kristine: 2160, 3020, 3205, 3270, 3335, 3445, 7300, 9020
Williams, Lilcelia A.: 7360
Williams, Madison S.: 5215
Williams, Rachel: 7680
Williams, Rebecca: 7620
Williams, Rhiannon: 9030
Williams, Shellie: 9010
Williams, Valerie: 9000
Williams, Victoria: 1000
Williams-Farrelly, Monica M.: 5115, 9020
Williamson, Heather: 7400
Williamson, Jeff D.: 4134, 5230, 7830
Willis, Angelique B.: 9010, 9310
Willis, Deanna R.: 4210, 5105
Willis, Sherry L.: 3510, 4225
Willroth, Emily: 1255, 3230, 3245, 3520, 9020
Wilmanski, Tomasz: 5180, 7880
Wilmes, Paul: 1225
Wilmoth, Janet M.: 1160, 7970
Wilmoth, Kristin: 7760
Wilson, Fernando: 1155
Wilson, Haley: 3540
Wilson, Jason: 3560
Wilson, Jess: 7480
Wilson, Kamryn: 7070
Wilson, Lisa: 7160
Wilson, Megan E.: 4320, 9020
Wilson, Paige N.: 3205
Wilson, Sarah: 8900
Wilson, Stephanie J.: 4235, 4370, 7070
Wilson, Traci L.: 3045
Wilson- Menzfeld, Gemma: 9420
Windham, B. Gwen: 3510, 5085, 7340, 9200
Windham, William A.: 5085, 7340
Windsor, Liliane C.: 5115
Windsor, Tracy: 9000
Wing, Ian Sue: 2105
Wing, Rena R.: 4355, 7630
Wingood, Gina: 3565
Wingood, Mariana: 7340, 7990
Wininger, Michael: 7210
Winship, Jodi: 3025

Winslow, Lauren: 7660
Winslow, Victoria: 7230
Winstead, Vicki: 5140
Winters, Emily D.: 4408, 7700
Winters-Stone, Kerri M.: 7680
Winton, Rebecca: 9400, 9440
Winton, Tish: 4415
Wipke-Tevis, Deidre: 9000
Wiranto, Yumiko: 4134, 7830
Wirth, Maria: 3100, 3195
Wisch, Julie: 9030
Wister, Andrew: 2230
Withers, Mellissa: 4266, 7190
Wittenberg, Grace: 4190
Wittich, Walter: 7650
Wittink, Marsha N.: 1335
Wittke, Michael: 3030, 4295
Witzel, Dakota D.: 1160, 3545, 4425, 5080, 7510, 9010
Wöhrmann, Anne M.: 7290
Wojczynski, Mary K.: 4260, 5090, 9010
Wolf, Michael S.: 9030
Wolfe, Alex: 9030
Wolfe, Hannah: 1105, 3195
Wolfe, Megan: 1330
Wolfe, Robert R.: 4130, 7880
Wolfe, Warren: 1025, 7470
Wolff, Jennifer: 1035, 2185, 3415, 4170, 5130, 5160, 9000, 9020
Wolf-Ostermann, Karin: 2370
Wolfson, Julian: 9410
Won, Chang Won: 7630, 9020
Won, Sarah: 7400, 9020
Wong, Bob: 1150, 1250
Wong, Chek Hooi: 3470
Wong, Chelsea N.: 9010, 9430
Wong, Gloria Hoi-Yan: 5235, 7330, 7420, 7760, 7920, 9420
Wong, Ian C.K.: 7330
Wong, Jennifer L.: 7000
Wong, Jennifer A.: 7230
Wong, Joey: 2340, 3270, 9010, 9020
Wong, Karen Lok Yi: 9000, 9010
Wong, Kayla: 5235
Wong, Rebeca: 3160, 7320
Wong, Rita: 9010
Wong, Roger: 1010, 9020
Wong, Stella Lai-Kuen: 9420
Wongvibul, Thanchanok: 7090
Woo, Misuk: 7780
Woo, Seoyoon: 9020
Wood, Kristie A.: 7470
Wood, Leila: 9010
Wood, Mallory L.: 7090
Wood, Rachel E.: 3025
Wood, Shenell T.: 7840
Wood, Sinai: 3300
Woodard, John: 7510
Woodrell, Christopher D.: 7850
Woods, Ashley N.: 7140
Woods, Robyn L.: 7300, 7370
Woodsmall, Katelyn: 9030
Woodward, Amanda T.: 4155
Worthington, Amber K.: 9010, 9020
Wouters, Eveline JM.: 9210
Wouterse, Bram: 7120, 9010
Wretman, Christopher: 1210
Wrigglesworth, Jo: 7370
Wright, Alexi A.: 7680
Wright, Brittany: 7760
Wright, Cynthia F.: 9020
Wright, Drew: 7900
Wright, Kathy D.: 4255
Wright, Molly: 9030
Wright, Regina S.: 7410
Wright, Rhonda: 1345
Wright, Rollin: 4315
Wroblewski, Kristen: 1230
Wrzus, Cornelia: 7030
Wu, Bei: 1045, 1240, 1320, 2090, 2265, 2330, 3180, 3540, 4105, 4380, 5055, 5170, 7330, 9010, 9020, 9110, 9300, 9310, 9320
Wu, Chenkai: 4090, 7830
Wu, Deqing: 2015, 7110, 7370, 7800
Wu, Haorui: 4530
Wu, Hau-Tieng: 3390
Wu, Heng: 3370
Wu, Jianli: 7670
Wu, Jingchuan: 7610
Wu, Jingxuan: 5115, 7060, 7950
Wu, Kuan-Ching: 4505, 7300, 7910, 9010, 9020
Wu, Meng-Chen: 4134, 7830
Wu, Mingche MJ.: 2185, 5160
Wu, MJ Mingche: 2185, 5160
Wu, Qiao: 2325
Wu, Rachel: 7860
Wu, Rachel: 9030
Wu, Ruiqian: 7620
Wu, Shengyong: 7330
Wu, Shinyi: 1185, 3005, 9020
Wu, Xingxing: 7940
Wu, Yafei: 9110
Wu, Yanjuan: 2255
Wu, Yiyi: 3565, 7230
Wu, Yunfan: 9000
Wu, Yuqiong: 9000, 9020
Wu, Yuxia: 4240
Wu-Chung, Lydia: 9110
Wunderlich, Tiffany: 7440
Wyatt, Riley: 7510
Wyles, Christina L.: 4380
Wyman, Jean F.: 5100, 9030
Wyman, Mary F.: 3015, 5000
Wyndham-West, Michelle: 9000
Wynn, Matthew J.: 4210
Wyte-Lake, Tamar: 2295

**X**

Xi, Wanyu: 1245
Xi, Yitao: 9020
Xia, Qing: 9530
Xian, Hong: 7960
Xian, Ying: 2265
Xiang, Lucille: 9020
Xiang, Qiao: 7350
Xiang, Xiaoling: 5235, 7030, 9010
Xiao, Chunhong: 1075
Xiao, Danqing: 9200
Xiao, Mingzhao: 4535
Xie, Alice: 4460
Xie, Bo: 5235
Xie, Dawei: 7650
Xie, Rui: 2385, 5075, 5100, 7080, 7150, 7430, 7620, 7680, 7820, 9010, 9020
Xie, Zongxing: 7680
Xin, Meiqi: 4266, 7190
Xiong, Lile: 9020
Xiong, Mai: 3450
Xiong, Yuan: 9010, 9530
Xiong, Zhiyao: 9020
Xu, Bei-bei: 1125, 4310, 7930
Xu, Bill Tong: 9010
Xu, Bowen: 1110
Xu, Chun: 9200
Xu, Hanzhang: 2265, 3180, 9000, 9110
Xu, Huiwen: 1115, 9310
Xu, Hui-wen: 1125, 4310, 7930
Xu, Ling: 2390, 3305, 4240, 7760, 9030
Xu, Ming: 3575, 9030
Xu, Shu: 2135, 2250, 7830
Xu, Shuo: 2330
Xu, Tianwei: 9030
Xu, Ting: 2330
Xu, Ting: 9000
Xu, Wenqian: 9000
Xu, Ying: 7950
Xu, Yingzhi: 7070
Xue, Qianli: 1370, 4090, 4485, 5230
Xue, Tingzhong (Michelle): 9110
Xue, Wei- Lin: 4215, 7790

**Y**

Yadav, Hariom: 4475, 7100, 7870
Yaffe, Kristine: 2240
Yahr, Andrea: 2170
Yam, Hawking: 3020, 3460
Yamada, Yuji: 7150, 7860
Yamaguchi, Yuko: 9010
Yamakawa, Miyae: 9000
Yamamoto-Mitani, Noriko: 7160, 7720, 9520
Yamashita, Mariko: 7760
Yamashita, Takashi: 2375, 3310, 4345, 5080, 5110, 7040, 7380, 7600, 7950
Yamazaki, Sachiko: 7580
Yan, Changmin: 4268, 7680
Yan, Elsie: 1145, 7050, 9010
Yan, Mengzhao: 3365, 4375
Yan, Zi: 4132, 7500, 7780
Yancura, Loriena: 2005
Yang, Amy: 1290
Yang, Bing: 4535
Yang, Frances M.: 2160, 3335, 7300
Yang, Huiyu: 9010
Yang, Hwajin: 9000
Yang, Jie: 7450
Yang, Jinzhu: 9580
Yang, Jisun: 7850
Yang, KwangSoo: 5225
Yang, Mayla: 7380
Yang, Mia: 4355
Yang, Minhee: 7420
Yang, Peilin: 4205
Yang, Peisen: 9010, 9030
Yang, Qi: 2195
Yang, Rumei: 1260, 7120
Yang, Ruoqi: 1215
Yang, Wei: 1045
Yang, Xiufen: 9020, 9520, 9540
Yang, Yang Claire: 1320
Yang, Yang: 4240

Yang, Yulin: 4115, 4250, 7540
Yang, Zhongfang: 7140, 7330
Yang, Zhongfang: 9010
Yang, Zhou: 1125, 4310, 7930
Yankeelov, Pamela: 4040
Yao, Jingxin: 9440
Yao, Shanshan: 5090, 7630, 9300, 9330
Yao, Yao: 9020
Yaphe, Sean R.: 2225
Yasaka, Taisuke: 9520
Yasar, Sevil: 9400
Yashin, Anatoliy: 2015, 5015, 5110, 7110, 7370, 7620, 7800
Yashkin, Arseniy P.: 2015, 2180, 3510, 5015, 5110, 7100, 7370, 7620
Yau, Jessie Ho-Yin: 7760, 9420
Yauk, Jessica: 3345, 9030
Ye, Fan: 5030, 7120, 7680
Ye, Justin: 3230
Ye, Minzhi: 2005, 2260
Ye, Nina Ni: 4185
Yeatts, Dale E.: 5185
Yee, Claire I.: 1305, 9320
Yee, Joanne: 9020
Yefimova, Maria: 5140
Yeh, Jarmin: 9020
Yelland, Erin: 7590
Yen, Dorothy: 9010
Yeo, Hyesu: 7220, 7290, 9000
Yeoman, Carl J.: 4080
Yeshua-Katz, Daphna: 2345
Yetman, Linda: 9010
Yeung, Dannii Y.: 3280, 7610, 9010
Yeung, Ellen: 5005
Yi, Stella: 7230
Yildiz, Mustafa: 5140
Yin, Hongyu: 9030
Yin, Lijuan: 4465
Yin, Na: 3080
Yin, Yueheng: 7150
Ylitalo, Kelly: 7570
Yokota, Fumihiko: 7940
Yolken, Robert: 7870
Yonashiro-Cho, Jeanine: 2220
Yoneda, Tomiko: 1255, 3520
Yong, Roseline: 7580
Yong, Ting: 1055
Yoo, Hyun Joo: 1215
Yoo, Jee-Hye: 7400
Yoo, Ji: 4075, 5185
Yoo, Subin: 7400
Yoo-Jeong, Moka: 9000
Yook, Soonhyun: 4265
Yoon, Caroline: 9530
Yoon, Dokyung: 7540
Yoon, Hee Jeong: 9540
Yoon, Jong Sung: 7960
Yoon, Seolah: 9000
Yoon, Sukyung: 7760
Yoon, Yeojin: 9000, 9030
Yorgason, Jeremy B.: 4235, 4370, 7410
Yoshida, Hiroko: 4505, 9000
Yoshikawa, Aya: 7430
You, Suha: 7960
Youn, Joy: 1380
Younan, Diana: 2320
Young, Amy: 9030
Young, Bethany: 8900
Young, Heather M.: 9030
Young, Jessica: 1160
Young, Kashyah S.: 9010
Young, Lufei: 9010, 9500
Young, Vanessa: 4100
Young, Yuchi: 3365, 7260
Young, Yvette: 2050
Yount, Naomi: 2375
Yous, Marie-Lee: 7840
Yousefi Nooraie, Reza: 2145
Yousefzadeh, Matt: 4475
Yow, W. Quin: 4185
Yu, Doris S.F. SF.: 1140, 5225, 7300, 7520
Yu, Fang: 9030, 9200
Yu, Guo: 7530
Yu, Heshuo: 7900
Yu, Junwen: 7140, 7330, 9010
Yu, Kexin: 1155, 4100, 4160
Yu, Miao: 2320
Yu, Wenshan: 9030
Yu, Wing-Lam: 4520
Yu, Xinfang: 4535
Yu, Xuexin: 2280
Yuan, Dianqi: 9030
Yuan, Rong: 9020
Yuan, Yiyang: 3105
Yue, Courtney Sen Yee: 7060
Yue, Jirong: 7350, 7660, 7830
Yue, Yiwei: 4265, 4490, 7870
Yun, Hyunkyung: 2035, 5215
Yun, Seon Young: 7810
Yun, Stacy W.: 7090
Yung, Raymond: 9030
Yunus, Raudah Mohd: 4266, 7190

### Z

Zachmeyer, Morgan L.: 2160
Zadok, Erez: 5030, 7120
Zafar, Anum: 1240, 4105, 7800, 9000
Zagorski, William A.: 1095
Zahedikia, Negin: 2280
Zahir, Zohaib: 9010
Zahodne, Laura B.: 1170, 1355, 2100, 3440, 4325
Zakak, Nicole: 7410
Zakrajsek, Jennifer S.: 9030
Zakrzewski, Chris: 3300
Zalyesov, Eduard: 7720
Zambrano, Priscilla L.: 9020
Zambrano Garza, Elizabeth: 3275, 7550
Zamora, Alejandra: 5080
Zampino, Marta: 9020
Zamrini, Edward: 3490
Zanaj, Skerdi: 1225
Zanasi, Francesca: 9030
Zang, Emma: 1305, 9440
Zanier, Nicole: 9030
Zaninotto, Paola: 3265, 4015
Zanjani, Faika: 9000
Zanoni, Joseph P.: 4465
Zaragoza, Gabriela: 7180
Zarling, Amie: 3445
Zaslavsky, Oleg: 4505, 7300, 9020
Zauszniewski, Jaclene A.: 2005, 5120, 7030, 9020
Zavala, Daisy: 1255, 4045
Zavala, Patrick: 7830
Zebrak, Katarzyna: 2375
Zeiner, Tracey: 5190, 7680
Zeisel, John: 9400
Zeki Al Hazzouri, Adina: 9110, 9500
Zelinski, Elizabeth: 7380
Zell, Angela: 4155
Zeller, Andreas: 3115
Zemon, Kevin A.: 7080
Zeng, Derong: 9010
Zenobia-Moore, Ann: 2195
Zepel, Lindsay: 7150
Zequeira, Sabrina: 1030
Zernial, Carol: 2160
Zettel-Watson, Laura: 7070
Zeziulin, Oleksandr: 2055
Zhan, Rong-Fang: 9000
Zhan, Yan: 9030
Zhang, Bohan: 5060, 7370
Zhang, Chen: 2330
Zhang, Cuntai: 9000
Zhang, Jiaan: 7560
Zhang, Liangwen: 9560
Zhang, Lingling: 2225, 7960
Zhang, Meina: 7990
Zhang, Meng: 2385
Zhang, Ming: 7930
Zhang, Mutian: 7380
Zhang, Nan: 7030, 7950
Zhang, Ning Jackie: 4240
Zhang, Peiyuan: 1035, 2330, 5005, 5015, 5025, 7260
Zhang, Qing: 9000
Zhang, Qiuyang: 2260
Zhang, Ruoying: 7380
Zhang, Shenghao: 1040, 3025, 7590, 9030
Zhang, Shifang: 7100, 7620
Zhang, Shiyang: 1255, 7020, 7070
Zhang, Talan L.: 1035, 2150, 4170
Zhang, Wei: 9010
Zhang, Weiwei: 9540
Zhang, Wen: 7920
Zhang, Wencheng: 4400, 7950
Zhang, Wenhan: 7620
Zhang, Wuyang: 2150, 9010
Zhang, Xiaomin: 7370, 7880
Zhang, Xin: 1245
Zhang, Xinyu: 3555, 9000
Zhang, Xinyu: 9030
Zhang, Xiping: 7370
Zhang, Xuehan: 7010, 9010, 9030
Zhang, Xuezhixing: 9440
Zhang, Yalu: 3220
Zhang, Yan: 3105
Zhang, Yao: 2225
Zhang, Yiming: 7870
Zhang, Ying: 7620
Zhang, Yingyang: 2280
Zhang, Yuan: 3225, 3440, 7380
Zhang, Yuankai: 9030
Zhang, Yucong: 9000
Zhang, Yue: 9000
Zhang, Yun: 2230, 9320
Zhang, Zhenmei: 4230, 7530, 9010
Zhao, Dandan: 7960
Zhao, Erfei: 1125
Zhao, Heng: 5115, 7060, 7950
Zhao, Hui: 2260, 3485, 9010
Zhao, Liang: 7150
Zhao, Lingya: 4240

Zhao, Qianhua: 9020
Zhao, Shangang: 4405
Zhao, Wei: 2320, 7630
Zhao, Yinan: 7640
Zhao, Yong: 9010
Zhao, Yuanchang: 7030, 7410
Zhao, Yuxiang: 1245
Zheng, Anqing: 3080, 4185
Zheng, Chuxuan: 5235, 7030
Zheng, D. Diane: 9010
Zheng, Rong: 9020
Zheng, Yaguang: 1320, 2265, 3540
Zhong, Chuwen: 3080, 5010
Zhong, Lily: 9410
Zhong, Selena: 1230
Zhou, Chuankai (Kai): 3355
Zhou, Huiquan: 3510
Zhou, Jiajia: 1130, 2225
Zhou, Jiayi: 2285
Zhou, Jiayi: 3510
Zhou, Junhong: 1260, 2155, 3510
Zhou, Leyi: 7280
Zhou, Nan: 4305, 7030
Zhou, Shuai: 2225, 2230, 7250
Zhou, Yuanjin: 1280, 2385, 7430
Zhou, Yunshu: 2135
Zhou, Zexi: 5045, 7070, 7920
Zhu, Alex Yue Feng: 7930
Zhu, Change: 9010
Zhu, Enya: 7020
Zhu, Haidong: 9010, 9500
Zhu, Jiafeng: 4485
Zhu, Jiangyuan: 9020
Zhu, Jianmin: 9000
Zhu, Jiayi: 9010
Zhu, Jingmin: 4015
Zhu, Shijun: 4005, 9020
Zhu, Xiang: 4475
Zhu, Xiaonan: 1015
Zhu, Yating: 4195, 7590
Zhu, Yujun: 3365
Zhu, Yun: 4475, 9020
Zhu, Yuyang: 7600, 9020
Zhu, Zheng: 2090, 2265, 3540, 7140, 7330, 9010, 9020
Zieschang, Tania: 7830
Ziglor, Candace: 4300
Zimami, Sumiyyah: 9000
Zimmer, Zachary: 2050
Zimmerman, Kristin: 9030
Zimmerman, Lindsey: 2145
Zimmerman, Sheryl: 1210, 3205, 3320, 4135, 7420, 9020, 9300
Zimmermann, Jaroslava: 4530
Zingmark, Magnus: 2340
Zipunnikov, Vadim: 2150
Zisberg, Anna: 7050
Ziyalan, Elvan: 9100
Ziylan, Canan: 7000
Zmuda, Joseph M.: 5090, 7830, 9010, 9020
Zoaktafi, Mojgan: 7960
Zolnoori, Maryam: 9010
Zomer, Helena D.: 1380, 7370
Zonderman, Alan B.: 4130, 7560, 9000
Zotcheva, Ekaterina: 9020
Zou, Ning: 5235
Zubatsky, Max: 4160, 9000, 9030
Zucchelli, Alberto: 2280
Zucchero, Renee' A.: 7460
Zuluaga, Maria Isabel: 9570
Zuniga, Franziska: 3180, 7160
Zuo, Xi: 7650
Zvonkovic, Anisa: 3060
Zwakhalen, Sandra: 3340, 7120, 7940
Zweibaum, David: 5085
Zweig, Richard: 5080
Zwerling, Jessica: 2265, 9000
